# Photo-clenbuterol: Optical Control of β_2_‑Adrenergic Receptor Signaling by Photoswitchable Ligand Efficacy

**DOI:** 10.1021/acs.jmedchem.5c00792

**Published:** 2025-06-10

**Authors:** Yangzhi Cao, Shuang Shi, Simone A. H. Does, Christian M. L. Buzink, Meichun Gao, Iwan J. P. de Esch, Henry F. Vischer, Maikel Wijtmans, Rob Leurs

**Affiliations:** Division of Medicinal Chemistry, Amsterdam Institute of Molecular and Life Sciences (AIMMS), 1190 Vrije Universiteit Amsterdam, 1081 HZ Amsterdam, The Netherlands

## Abstract

Clenbuterol is a potent partial agonist on the human β_2_-adrenergic receptor (β_2_-AR) and available for veterinary use to treat respiratory diseases. We executed an “*azoextension*” strategy to generate a small library of photoresponsive azobenzene derivatives of clenbuterol. Illumination with two complementary wavelengths allowed interconversion between isomeric *trans* and *cis* forms, as proven by UV/vis, NMR and LC–MS studies. The photoswitchable clenbuterol analogs were pharmacologically characterized using β_2_-AR radioligand binding and cAMP assays. Key compound **12b** (VUF26034) has suitable photochemical properties and good thermal stability of the *cis* isomer (*t*_1/2_ ∼ 4 months), switching from a partial agonist to a >25-fold higher affinity antagonist upon illumination with 360 nm.

## Graphical abstract

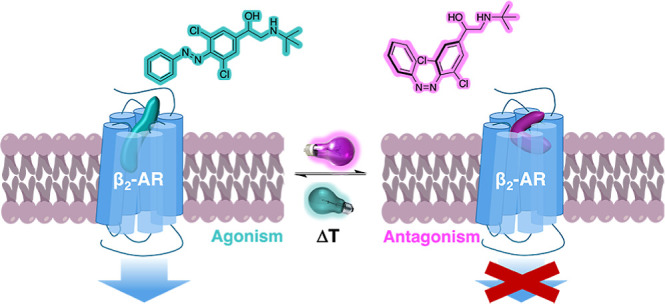

## Introduction

In photopharmacology, photoresponsive small-molecule ligands are used to modulate protein targets such as G protein-coupled receptors (GPCRs), enzymes, ion channels, and transporters. This is achieved by using light as an external trigger to enable high spatiotemporal modulation accuracy.^1–4^ There are two main chemical strategies in photopharmacology. In photocaging, a biologically active ligand is rendered inactive by introducing a photolabile protecting group. The active compound can be released locally with light in an irreversible process.^5,6^ In contrast, photoswitching is a reversible strategy in photopharmacology and involves introducing a photochromic moiety in a ligand structure with which ligand activity can be modulated reversibly using light. There are several classes of photochromic moieties, such as azobenzene, hemithioindigos and stilbenes.^2^ Azobenzene stands out as the most versatile photochromic moiety, as it can undergo light-induced reversible *trans*–*cis* photoisomerization, leading to a change in its configuration and in the biological properties of the ligand in which the azobenzene is embedded.^7,8^

The application of photopharmacology to GPCRs has attracted considerable interest.^9–12^ GPCRs are seven- transmembrane-domain proteins and constitute one of the most preferred families of drug targets, as evidenced by ∼ 34% of the currently used drugs acting on GPCRs.^13^ Adrenergic receptors (ARs) are Class A GPCRs that recognize the endogenous signaling molecules adrenaline and noradrenaline. Among the subtypes of ARs, the β_2_-adrenergic receptor (β_2_-AR) is an important target that mediates physiologic responses such as smooth muscle relaxation and bronchodilation.^14^ It is also among the best-studied GPCR members and as such provides an excellent case study for GPCR photopharmacology. To date, several photoswitchable β_2_ antagonists based on propranolol have been reported.^15,16^ At the onset of our work, no photoswitchable β_2_ agonists were reported but during the course of our work, a series of photoswitchable analogs of adrenaline was reported.^17^ Specifically, an arylazopyrazole-based adrenaline derivative was shown to harbor a *trans*-on affinity photoswitch (Δp*K*_i_ = 0.63), with both the *trans* and *cis* isomers being full agonists (ΔpEC_50_ = 0.33).

We focused our efforts on clenbuterol, a selective potent β_2_ agonist that is approved as its racemate for veterinary use as a therapeutic drug to treat respiratory diseases.^18,19^ Interestingly, an azobenzene derivative of clenbuterol and bisphenol A has been reported in pursuit of derivatization techniques for sensitive analysis of clenbuterol and bisphenol A in meat and water samples.^20,21^ However, no photoswitchable properties of the adduct were reported and it is likely that the presence of the *o*-OH group in the adduct prevents any practical utility as a photoswitchable unit because of very fast thermal relaxation of the *cis* to *trans* isomer.^22,23^ Here, we report five azobenzene derivatives, of which **12b** (dubbed “photo-clenbuterol”) shows acceptable photochemical properties, good thermal stability of the *cis* isomer (*t*_1/2_ ∼ 4 months), >25-fold gain in binding affinity upon switching from *trans* to *cis* and, interestingly, different efficacies for the *trans* (partial agonist) and *cis* (antagonist) isomers. This photoinduced efficacy switch makes photo-clenbuterol complementary to existing photoswitchable tools for β_2_-AR.

## Results and Discussion

### Design

Inspired by the general feasibility of a clenbuterol-azobenzene scaffold, we selected azoextension^7^ of clenbuterol as a starting point for our work (Figure 1A). To support the azoextension hypothesis, molecular superposition analyses were conducted for clenbuterol and its direct azoextension derivatives *trans*-**12b** and *cis*-**12b** (Figure 1B). Previous studies have demonstrated that racemic clenbuterol exhibits significantly higher binding affinity than the S-(+)-enantiomer,^24^ establishing R-(−)-clenbuterol as the pharmacologically active enantiomer. Consequently, we selected R-(−)-clenbuterol for molecular superposition studies. The cocrystallized ligand BI-167107 (PDB: 3P0G) in its bioactive conformation was used as a structural reference. The superposition highlights conformational similarity among BI-167107, clenbuterol, *trans*-**12b**, and *cis*-**12b** (Figure 1C,D). This suggests that both isomers of **12b** can adopt binding modes within the binding pocket of β_2_-AR similar to those of BI-167107 and clenbuterol. However, in contrast to *trans*-**12b**, *cis*-**12b** exhibits a nonplanar geometry. Thus, the distinct geometrical features of the two isomers were postulated to lead to different binding interactions within the receptor pocket, allowing one isomer to exhibit improved binding to β_2_-AR.

**Figure 1:**
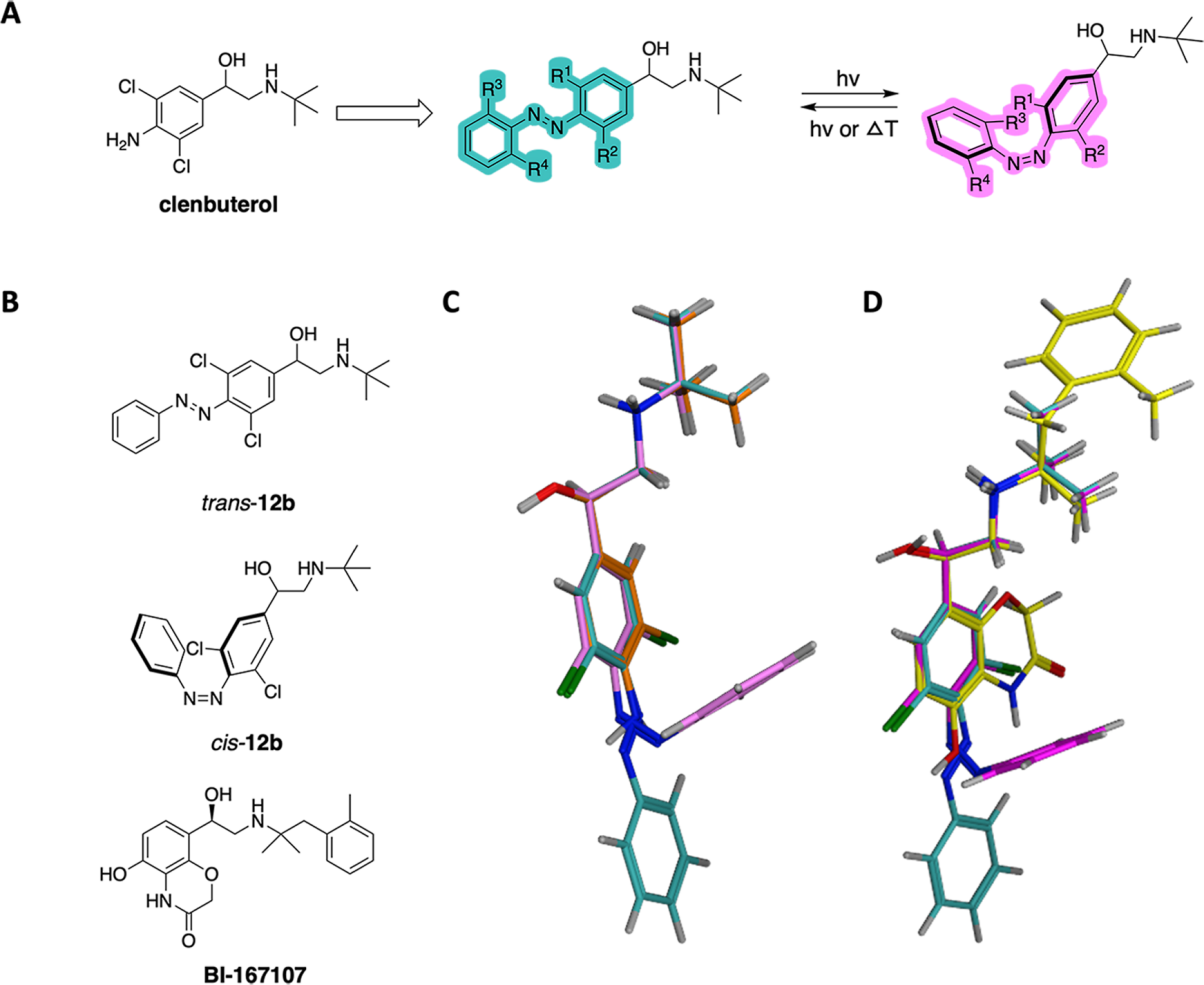
(A) General design and concept of photoswitchable β_2_-AR agonists based on clenbuterol. (B) Structures of azoextension-derivatives *trans*-**12b** (cyan), *cis*-**12b** (magenta), and reference ligand BI-167107. (C) Superposition of clenbuterol (orange carbon atoms), *trans*-**12b** (cyan carbon atoms), and *cis*-**12b** (magenta carbon atoms) using the reference ligand BI-167107 in its bioactive conformation. (D) Superposition of *trans*-**12b** (cyan carbon atoms), *cis*-**12b** (magenta carbon atoms) and BI-167107 (yellow carbon atoms). BI-167107 was fixed in its bioactive conformation obtained from the published ligand–protein structure (PDB: 3P0G). The stereochemistry at the chiral carbon atom of *trans*-**12b** and *cis*-**12b** was kept the same (i.e., *R*), in line with that of BI-167107 and of the more active enantiomer of clenbuterol.

### Synthesis

Figure 1 illustrates the general design strategy, which involved an azoextension of clenbuterol. Azobenzene analogs **12a-d** contain zero, one, two (as in clenbuterol itself) or four^25^ chlorine atoms. In addition, based on the published structure–activity relationship of clenbuterol,^26^ we also designed the chlorocyano-substituted compound **12e**. In 1984, Engelhardt et al.^26^ reported that substituents on the benzene ring of clenbuterol increased the bronchodilation ability of guinea pigs compared to clenbuterol. The strongest effect was reported for, among others, clenbuterol with a chloro-cyano substitution pattern instead of a dichloro-substitution pattern. We synthesized chloro-cyano clenbuterol (**18**, Scheme 3) as a control compound. Since clenbuterol itself is marketed as its racemate, we pursued racemic mixtures of **12a-e** and **18**.

Various synthesis routes were utilized toward **12a-e**. Scheme 1 shows the synthesis of **12a**, **b**. The synthesis of **12a** commenced with the reduction and ring closure of commercially available bromoketone **1** to afford epoxide **2**. This was followed by epoxide opening with *t*-BuNH_2_ and reduction of the NO_2_ group using Pd/C under H_2_ atmosphere to afford aniline **4**. In related work we had observed amide formation between the secondary alkylamine of β_2_ ligands and the common additive AcOH during the Mills reaction. Therefore, since prolonged heating was anticipated for the Mills reaction with **4**, sterically more hindered acids were explored. Gratifyingly, use of pivalic acid and 1-adamantanecarboxylic acid provided **12a** in modest 21% and 33% yield, respectively. Clenbuterol was oxidized to nitrosobenzene **5** using Oxone. After workup, it was directly used in a Mills reaction with PhNH_2_ to yield **12b**.

**Scheme 1:**
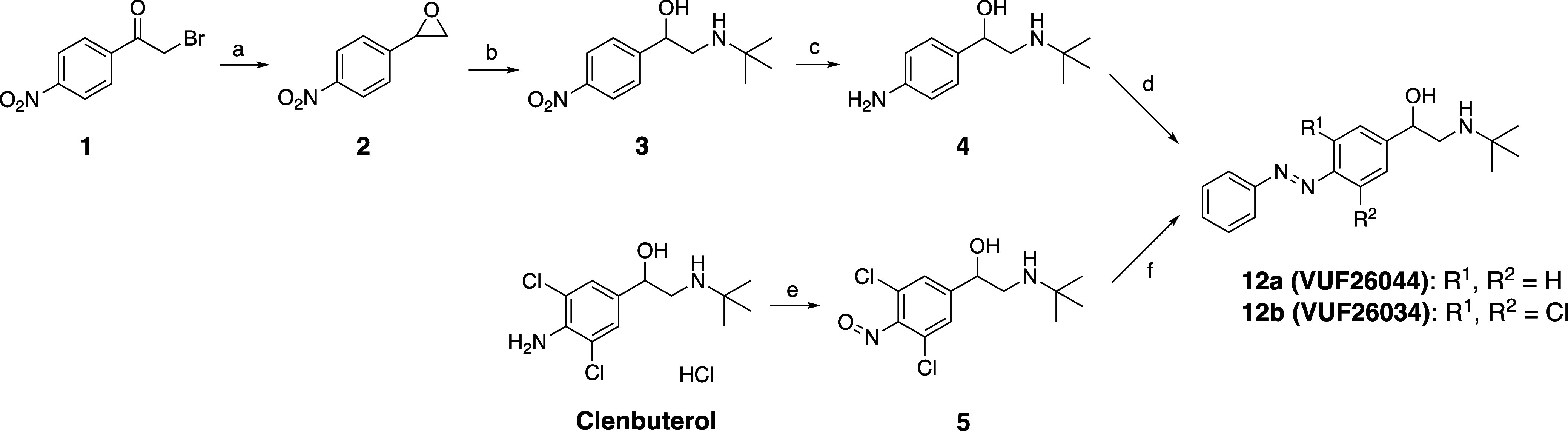
Synthesis of Compounds **12a** and **12b** ^a^

a Reagents and conditions: (a) [1] NaBH_4_, MeOH, 0 °C, 2 h; [2] K_2_CO_3_, 0 °C to rt, overnight; (b) *t*-BuNH_2_, EtOH, 75 °C, overnight, 80% for two steps from **1**; (c) Pd/C, H_2_, 1.0 M HCl, EtOH, rt, overnight, 66%; (d) PhNO, 1-adamantanecarboxylic acid, DCM, 40 °C, 3 d, 33%; (e) Oxone, DCM/H_2_O 1/4, rt, 2 h; (f) PhNH_2_, 1-adamantanecarboxylic acid, DCM, 40 °C, overnight, 24% for two steps from clenbuterol.

Scheme 2 shows the synthesis of **12c–e**. Aniline **6c** was treated with NBS in PhMe to obtain **6f**. Anilines **6a**, **6c**, and **6f** were oxidized to the corresponding nitrosobenzenes **7a**, **7c**, and **7f** using Oxone or *m*-CPBA. After workup, these were directly used in a Mills reaction with PhNH_2_ to afford azobenzenes **8a**, **8c**, and **8f**. Reduction by DIBAL-H yielded the alcohols **9a**, **9c** and **9f** in generally good yields. Pd-mediated cross-coupling using **9f** and Zn­(CN)_2_ furnished chlorocyano-substituted compound **9e**.^27,28^ Alcohols **9a**, **9c,** and **9e** were oxidized with Dess-Martin periodinane to benzaldehydes **10a**, **10c** and **10e**. A direct Pd-catalyzed C–H halogenation with **10a** led to tetra-*ortho*-chloro azobenzene **10d**.^29,30^ Corey–Chaykovsky reaction on aldehydes **10c–e** afforded epoxides **11c–e**. Epoxide opening with *t*-BuNH_2_ yielded the desired compounds **12c–e**.

**Scheme 2:**
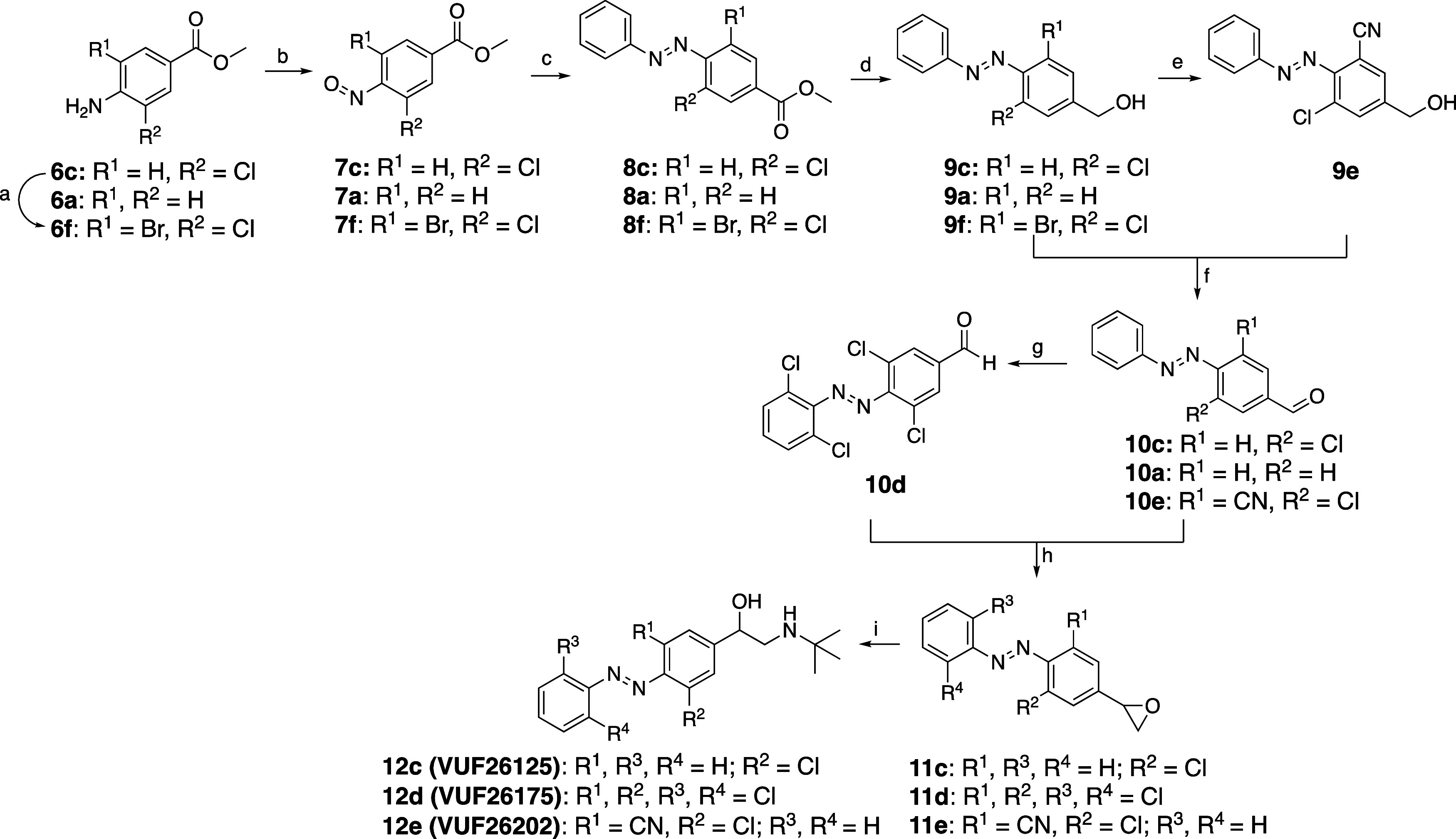
Synthesis of Compounds **12c–e** ^a^

a Reagents and conditions: (a) with **6c**: NBS, PhMe, 35 °C, 10 min, 89%; (b) **6a** or **6c**: Oxone, DCM/H_2_O 1/4, rt, 24–36 h; **6f**: *m*-CPBA, DCM, 0 °C to rt, 36 h; (c) PhNH_2_, AcOH, DCM, rt, 2–24 h, 12–55% for two steps from **6**; (d) DIBAL-H, THF, 0 °C to rt, 2 h, 86–88%; (e) With **9f**: Zn­(CN)_2_, Pd­(PPh_3_)_4_, DMF, 100 °C, 3 h, 47%; (f) Dess–Martin reagent, DCM, 0 °C to rt, 1 h, 85–90%; (g) With **10a**: NCS, Pd­(OAc)_2_, AcOH, 140 °C, 13 h, 49%; (h) Trimethylsulfonium iodide, NaH, DMSO/THF, 0 °C to rt, 1 h, 8–91%. (i) *t*-BuNH_2_, EtOH, 75 °C, overnight, 45–58%.

The synthetic route for **18** is illustrated in Scheme 3 and was based on a reported one.^26^ The synthesis started with aniline **13**, which was brominated using NBS to yield compound **14**. This underwent a reaction with CuCN in DMF to produce **15**, which was treated with NCS to afford **16**. Subsequent bromination of **16** using CuBr_2_ resulted in compound **17**, which underwent nucleophilic substitution and reduction to obtain compound **18**.

**Scheme 3:**
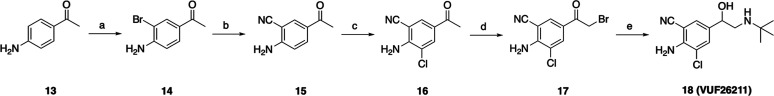
Synthesis of Compound **18** ^a^

a Reagents and conditions: (a) NBS, PhMe, 35 °C, 10 min, 75%; (b) CuCN, DMF, 160 °C, 6 h, 56%; (c) NCS, MeCN, 60 °C, 3 h, 41%; (d) CuBr_2_, THF, 70 °C, overnight, 55%; (e) [1] *t*-BuNH_2_, EtOH, 0 °C to rt, overnight; [2] NaBH_4_, 0 °C, 1 h, 25%.

### Photochemistry

The photochemical properties of **12a**-**e** were evaluated by recording the UV/vis absorption spectra before and after illumination (Figures 2A and S1B–S4B). All compounds have λ_max_ values for the π–π* transition of the *trans* isomer between 293 and 331 nm (Table 1). Similarly, λ_max_ values for the n−π* transition of the *cis* isomer (as approximated from λ_max_ values of the PSS_*cis*_ composition) differ only moderately, ranging from 417 to 441 nm (Table 1). After illumination with 360 ± 20 nm (except **12d**, for which 520 ± 12 nm was used), the values for the photostationary states (PSS) derived from LC–MS analysis range between 38.5 and 97.6% *cis* (Table 1, Figures 2B, S1C–S4C and S5). Subsequent illumination of these PSS_*cis*_ states with 434 ± 9 nm light affected *cis*–*trans* isomerization, affording PSS_*trans*_ states containing 65.5–94.1% *trans* isomer (Table 1, Figures 2B, S1C–S4C and S5). ^1^H NMR experiments (*vide infra*) conducted on **12a–e** reveal satisfactory agreement between PSS values derived from LC–MS and ^1^H NMR analyses (Figures 2C and S6–S10). All compounds demonstrate slow thermal relaxation (*t*_1/2_ >10 days) at 25 °C (Table 1, Figures S1D–S4D and S11).

**Figure 2:**
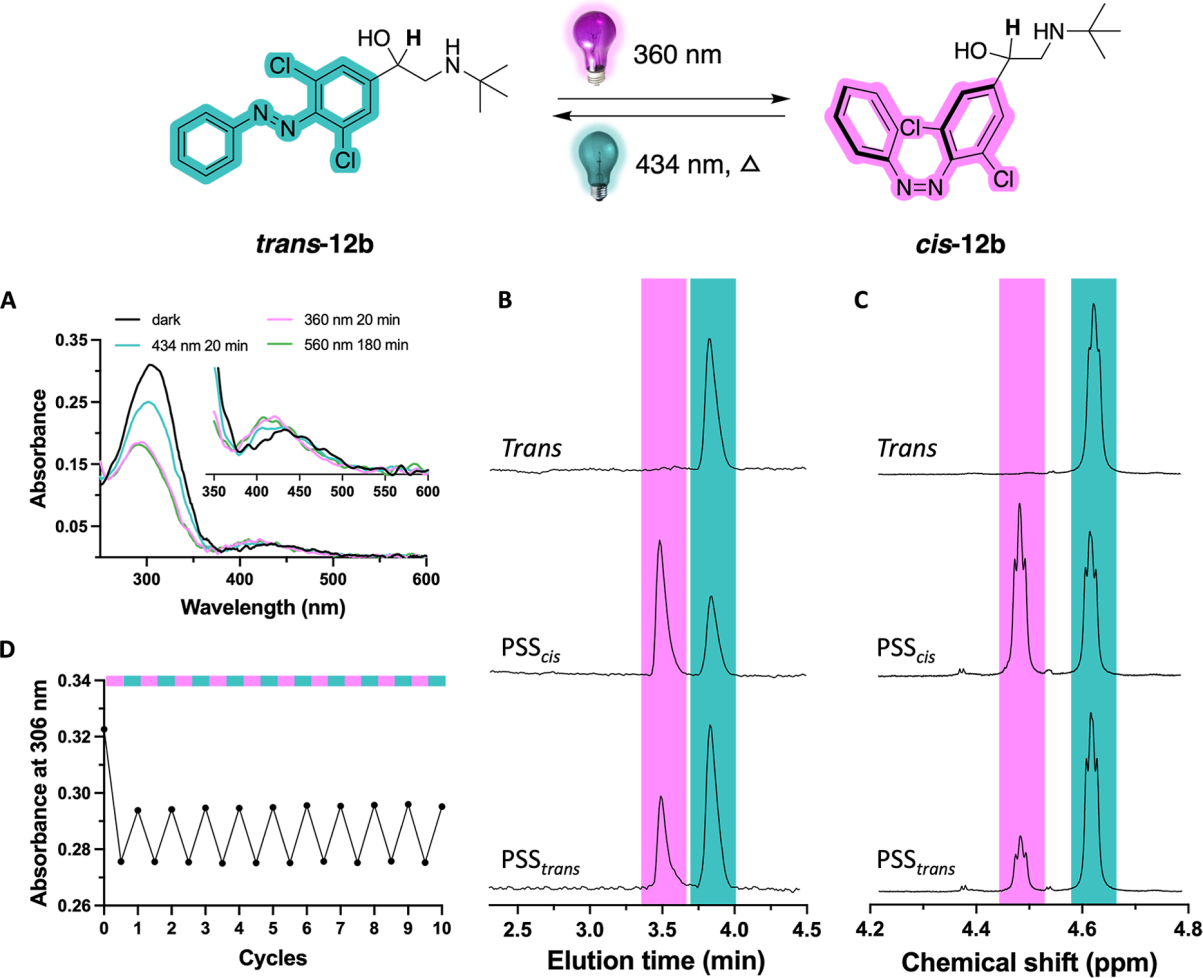
Photochemical characterization of key compound **12b**. (A) UV–vis spectra of 25 μM of **12b** in HBSS buffer containing 1% DMSO as the *trans* isomer (black), after illumination with 360 ± 20 nm (magenta) for 20 min to PSS_*cis*_ and after subsequent illumination with 434 ± 9 nm (cyan) for 20 min to PSS_*trans*_. The effect of illuminating with 560 ± 5 nm (green) for 180 min to PSS_*cis*_ is also included. (B) Representative part of LC–MS chromatograms of **12b** belonging to the illuminated ^1^H NMR sample shown in Figure 2C. Full LC–MS chromatograms are available in Figure S5. (C) Representative part of ^1^H NMR spectra of 10 mM **12b** in DMSO-*d*_6_ as *trans* isomer, after illumination with 360 ± 20 nm for 1200 s to PSS_*cis*_ and after subsequent illumination with 434 ± 9 nm for 1200 s to PSS_*trans*_. The presented peaks belong to the hydrogen atoms drawn in bold. Full ^1^H NMR spectra are available in Figure S7. (D) Reversible isomerization cycles of 25 μM of **12b** in HBSS buffer containing 1% DMSO with 365 ± 11 nm to reach PSS_*cis*_ and 440 ± 25 nm to reach PSS_*trans*_.

**Table 1:**
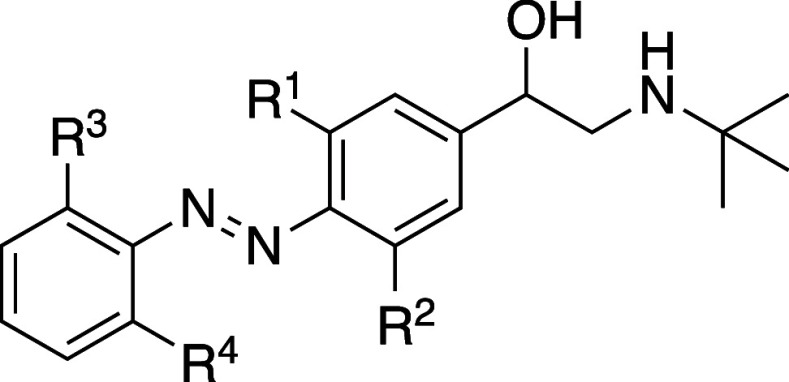
Photochemical Properties and β_2_-AR Binding Affinities of Photoswitchable Clenbuterol Analogs

Compound	**R** ^**1**^	**R** ^**2**^	**R** ^**3**^	**R** ^**4**^	λ_max_ (nm) *trans* ^a^	λ_max_ (nm) PSS_*cis*_ ^a^	*t*_1/2_ (days)^b^	PSS_*cis*_ (% *cis*)^c^	PSS_*trans*_ (% *trans*)^d^	p*K*_i_ *trans* ^e^	p*K*_i_ PSS*_cis_* e	p*K*_i_ shift^f^
**12a**	H	H	H	H	323	425	29	94.6 ± 0.5	71.8 ± 0.2	<5.0	6.0 ± 0.1	>1.0
**12b**	Cl	Cl	H	H	306	417	112	66.3 ± 0.9	72.5 ± 0.1	<5.0	6.4 ± 0.2	>1.4
**12c**	H	Cl	H	H	328	428	38	97.6 ± 0.1	65.5 ± 0.2	<5.0	6.8 ± 0.1	>1.8
**12d**	Cl	Cl	Cl	Cl	293	441	26	38.5 ± 0.5	94.1 ± 0.3	6.0 ± 0.1	7.0 ± 0.1	1.0 ± 0.1
**12e**	CN	Cl	H	H	331	426	11	88.7 ± 0.8	82.0 ± 0.4	<5.0	<5.0	N/D
**18**					N/A	N/A	N/A	N/A	N/A	7.7 ± 0.1	N/A	N/A
**clenbuterol**					N/A	N/A	N/A	N/A	N/A	7.8 ± 0.2	N/A	N/A

a Determined at 25 μM in HBSS buffer containing 1% DMSO.

b Approximate thermal relaxation half-life of the PSS_*cis*_ state at room temperature (25 °C) estimated with an Arrhenius approach. Measured in 25 μM HBSS buffer containing 1% DMSO.

c Measured in DMSO (10 mM) after illumination with 360 ± 20 nm (except **12d**: 520 ± 12 nm) to PSS_*cis*_ at room temperature and defined as the percentage of the area of the *cis*-isomer compared with combined areas of *cis*- and *trans*-isomers as detected by LC–MS analysis with the corresponding isosbestic points wavelength: 390, 376, 393, 370 and 396 nm for **12a–e** respectively (*n* = 3, SD given).

d Measured in DMSO (10 mM) after illumination with 434 ± 9 nm from PSS_*cis*_ to PSS_*trans*_ at room temperature and defined as the percentage of the area of the *trans*-isomer compared with combined areas of *cis*- and *trans*-isomers as detected by LC–MS analysis with the corresponding isosbestic points wavelength: 390, 376, 393, 370 and 396 nm for **12a-e** respectively (*n* = 3, SD given).

e Affinity for *trans* state (without preillumination) and PSS_*cis*_ state (preilluminated to reach PSS_*cis*_ with 360 ± 20 mm or 520 ± 12 nm for **12d**). p*K*_i_ values were obtained from competition radioligand binding assays and calculated using the Cheng–Prusoff equation with a p*K*_d_ value of 0.1 nM for [^3^H]­dihydroalprenolol. For **18** and clenbuterol, the p*K*_i (*trans*)_ values are for the thermostationary state.

f p*K*_i_ shifts are determined as p*K*_i (PSS,*cis*)_ – p*K*_i (*trans*)_. Data are shown as mean ± SD for three independent experiments in duplicate. N/A, not applicable; N/D, not detectable. Ligand **18** and clenbuterol are shown as nonphotoswitchable reference ligands.

Based on the favorable photopharmacology results (*vide infra*), compound **12b** underwent additional photochemical characterization. It is of interest to point out that, to the best of our knowledge, the parent molecule (*E*)-1-(2,6-dichlorophenyl)-2-phenyldiazene, which is an implicit feature of the design of **12b**, has been studied in a synthetic context,^31^ but not experimentally as a photoswitchable unit. However, it has been computationally studied by Konrad et al.^32^ who calculated the n−π* band to be 444.5 nm for the *cis* isomer of the parent compound in the gas phase. We find an approximated experimental value of 417 nm (Table 1) for *para*-substituted *cis-* **12b** in buffer + 1% DMSO. The difference between calculated and experimental value could conceivably be the result of the buffer solvent not having been included in the calculations and/or the suboptimal *trans* to *cis* conversion at PSS_*cis*_ (PSS_*cis*_ = 66.3%). The work by Konrad et al.^32^ suggests that the *o*,*o*-dichloroazobenzene unit may provide benefits in terms of red-shifting the irradiation wavelengths. This is evident in the UV–vis spectrum of **12b** (Figure 2A), where in the n−π* area the PSS_*cis*_ and *trans* bands do not completely overlap as is typical for an unsubstituted azobenzene (inset Figure 2A). The PSS_*cis*_ curve is slightly blue-shifted, potentially enabling *trans*–*cis* isomerization at longer wavelengths. Indeed, isomerization of **12b** to PSS_*cis*_ can in principle be achieved using visible light (560 nm) with an increase in the PSS_*cis*_ value from 66.3% (360 nm, Figure S5) to 74.9% (560 nm, Figure S12). However, not unexpectedly given the low absorption at 560 nm, the illumination time required is much longer (hours versus 20 min with 360 nm illumination). Such longer illumination times would only be acceptable if the pharmacological differences between the isomers were much more pronounced, not requiring full conversions to PSS_*cis*_. Therefore, 360 nm was selected as the more suitable wavelength for illuminating **12b** for photopharmacology assays. We photochemically investigated the dark, PSS_*cis*_ (360 nm) and PSS_*trans*_ (434 nm) states of **12b** using LC–MS and ^1^H NMR analysis. The PSS_*cis*_ and PSS_*trans*_ values by LC–MS analysis (Figures 2B and S5) at the isosbestic point (376 nm) are 66.3% and 72.5%, respectively. These are similar to the 62.4% and 78.7% values obtained from ^1^H NMR analysis, in which the well-resolved signal of the benzylic CH group allowed quantification of the PSS values (Figures 2C and S7). Compound **12b** shows good resistance to photobleaching during at least 10 dynamic isomerization cycles under alternating illumination (Figure 2D). According to an Arrhenius fit (Figure S11), the approximate half-life of **12b** is ∼3 weeks at 37 °C or ∼4 months at 25 °C.

### Pharmacology

Due to the sufficiently long thermal relaxation half-lives, pharmacological assays could be conducted on both isomeric states of the compounds. The compounds were either kept in the dark to ensure over 99% *trans*-isomer or irradiated at 360 ± 20 nm (**12d**: 520 ± 12 nm) to reach PSS_*cis*_. Binding affinities of the photoswitchable ligands for β_2_-AR were determined by [^3^H]­DHA competition binding to human β_2_-AR, transiently expressed in HEK293-T cells. In this assay the control compounds clenbuterol and its cyano-analog **18** are nanomolar ligands for the β_2_-AR with similar p*K*_i_ values (Table 1 and Figure S13A). The PSS_*cis*_ states of photoswitchable ligands **12a–d** exhibit a higher binding affinity for β_2_-AR as compared to their *trans* isomers (Table 1 and Figure S14). Substitution of the azobenzene ring(s) with chlorine atoms results in higher binding affinity for PSS_*cis*_ (compare **12b–d** to **12a)**. Tetra-chloro-analogue **12d** exhibits the highest p*K*_i_ value of 7.0 ± 0.1 (Table 1 and Figure S14C). The *trans* isomers of **12a–d** display at least 10-fold lower binding affinities compared to the corresponding PSS_*cis*_ states, revealing a significant photoinduced *cis*-on affinity shift (Table 1). However, substitution with a cyano group (**12e**) impaired the binding of both *trans* and the PSS_*cis*_ state to β_2_-AR compared to **12b** (Table 1, Figures 3A and S14D).

**Figure 3:**
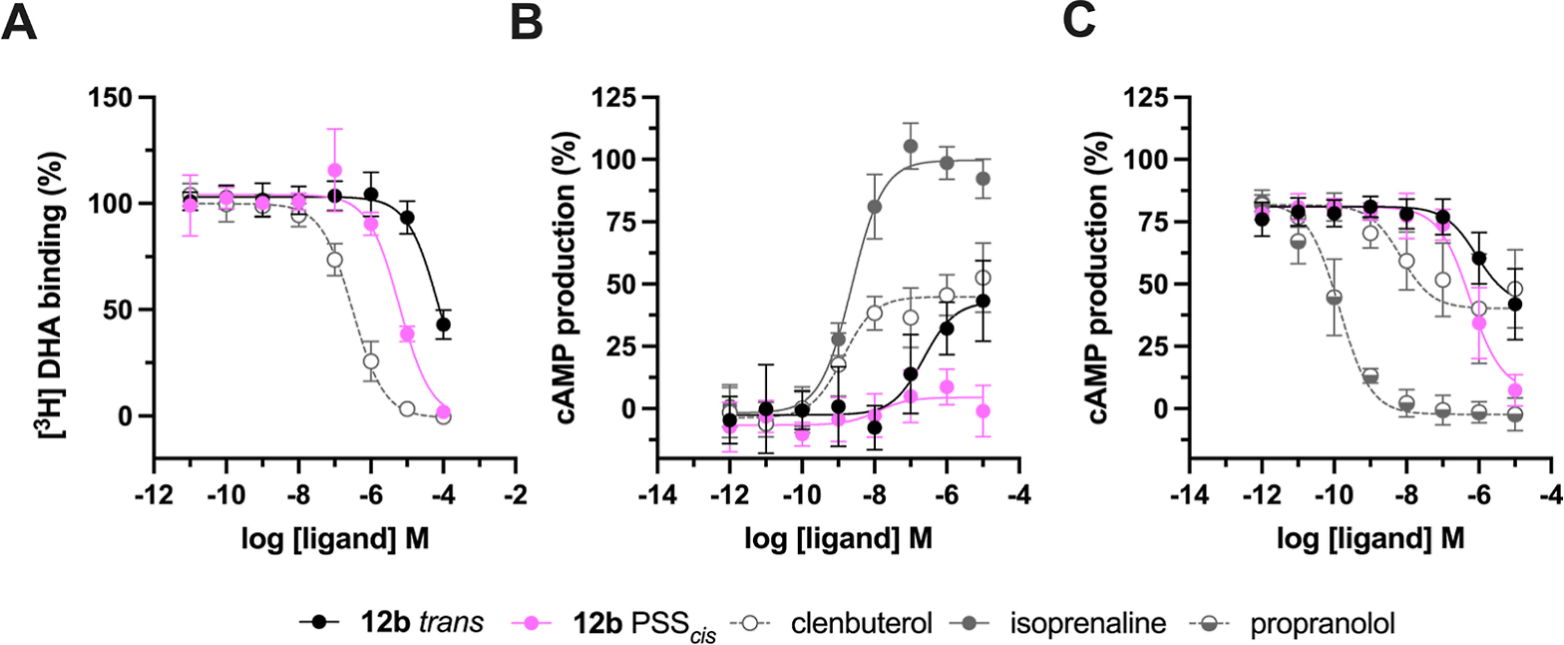
Pharmacological characterization of key compound **12b**. (A) Competition binding of 1.8–3 nM [^3^H]­DHA and increasing concentration unlabeled (photoswitchable) ligands to HEK293T membranes expressing human β_2_-AR. Endogenous β_2_-AR-mediated cAMP production in HEK293 cells expressing the FRET-based EPAC cAMP sensor fused with mCerulean and mCitrine on each side of Epac in response to (B) ligand stimulation and (C) antagonism of 13 nM isoprenaline (EC_80_ concentration) by indicated ligands. Clenbuterol, isoprenaline and propranolol are shown as β_2_-AR reference partial agonist, full agonist, and antagonist, respectively. Data are shown as mean ± SD for three independent experiments in duplicate.

The efficacy of ligands on β_2_-AR was evaluated using a Förster resonance energy transfer (FRET)-based EPAC cAMP sensor in HEK293 cells. In these cells endogenously expressing β_2_-AR, receptor activation with agonists isoprenaline, clenbuterol or **18** leads to the expected full (isoprenaline) or partial (clenbuterol, **18**) cAMP production at nanomolar concentrations (Table 2). Interestingly, photoswitchable clenbuterol analogs (*trans* or PSS_*cis*_) do not display partial agonism except for the *trans* isomer of **12b** which exhibits an intrinsic activity of 0.5 and a pEC_50_ of 6.7 ± 0.3 (Table 2, Figures 3 and S15). Thus, the azo-extension of clenbuterol results in a reduced affinity (and potency) of *trans-* **12b**, but does not affect the intrinsic activity for β_2_-AR activation.

**Table 2:**
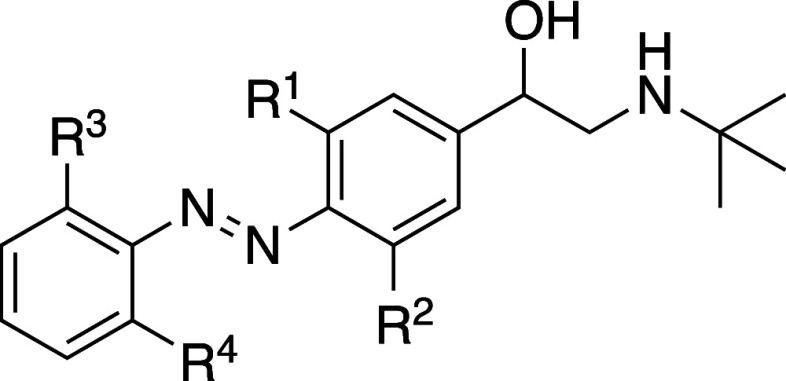
Efficacy of Photoswitchable Ligands on β_2_-AR-Mediated cAMP Production

Compound	R^1^	R^2^	R^3^	R^4^	pEC_50_ *trans* ^a^	pEC_50_ PSS_*cis*_ ^a^	pEC_50_ shift^b^	α^c^ *trans*	α PSS_*cis*_	p*K*_b_ *trans* ^d^	p*K*_b_ PSS*_cis_* ^d^	p*K*_b_ shift^e^
**12a**	H	H	H	H	N/D	N/D	N/D	0.0 ± 0.0	0.0 ± 0.0	5.3 ± 0.2	6.9 ± 0.0	1.6 ± 0.2
**12b**	Cl	Cl	H	H	6.7 ± 0.3	N/D	N/D	0.5 ± 0.2	0.1 ± 0.0	6.5 ± 0.5	6.9 ± 0.2	0.4 ± 0.4
**12c**	H	Cl	H	H	N/D	N/D	N/D	0.0 ± 0.0	0.0 ± 0.0	<5.0	7.4 ± 0.3	>2.4
**12d**	Cl	Cl	Cl	Cl	N/D	N/D	N/D	0.0 ± 0.0	0.0 ± 0.0	<5.0	7.0 ± 0.2	>2.0
**12e**	CN	Cl	H	H	N/D	N/D	N/D	0.0 ± 0.0	0.0 ± 0.0	<5.0	6.8 ± 0.2	>1.8
**18**					9.0 ± 0.0	N/A	N/A	0.8 ± 0.0	N/A	N/A	N/A	N/A
**clenbuterol**					8.9 ± 0.2	N/A	N/A	0.5 ± 0.1	N/A	8.9 ± 0.2	N/A	N/A

a Potency for *trans* state (without preillumination) and PSS_*cis*_ state (preillumined to reach PSS_*cis*_ with 360 ± 20 nm or 520 ± 12 nm for **12d**) of photoswitchable β_2_-AR ligands were determined using a FRET-based EPAC cAMP assay.

b pEC_50_ shift was defined as pEC_50 (PSS,*cis*)_ – pEC_50 (*trans*)_.

c Intrinsic activity (α) was determined by *E*_max (ligand)_/*E*_max (isoprenaline)_.

d Inhibitory potency (IC_50_) was acquired using FRET-based EPAC cAMP assay in the presence of 13–20 nM (EC_80_ value) isoprenaline to stimulate β_2_-AR and *K*_b_ values were calculated using the Cheng–Prusoff equation.

e p*K*_b_ shift was calculated by p*K*_b (PSS*,cis*)_ – p*K*_b (*trans*)_. Data are shown as mean ± SD for three independent experiments in duplicate. N/A, not applicable; N/D, not detectable. Isoprenaline was used as a reference full agonist with a pEC_50_ value of 8.6 ± 0.2 and α value of 1.0. Propranolol was used as a reference antagonist with a p*K*_b_ value of 10.6 ± 0.4. Ligand **18** and clenbuterol are shown as nonphotoswitchable reference ligands.

Next, the ability of the photoswitchable clenbuterol analogs to block isoprenaline-induced cAMP generation via β_2_-AR was assessed. The PSS_*cis*_ states of **12a**, **c**, **d**, **e** more potently antagonize the isoprenaline-induced cAMP response than the *trans* states do (Table 2 and Figure S16). Consequently, the calculated p*K*_b_ values using the Cheng–Prusoff equation exhibit higher values for PSS_*cis*_ compared to the *trans* state with a photoinduced shift of more than 10-fold (Table 2). This is comparable to the shift of p*K*_i_ value shown in Table 1. Interestingly, though, the PSS_*cis*_ of **12b** acts as an antagonist on β_2_-AR with a p*K*_b_ value of 6.9 ± 0.2, whereas its *trans* isomer acts as a partial agonist resulting in only a ∼50% inhibition of the isoprenaline-induced cAMP response, which is in line with the partial inhibition observed for clenbuterol (Figure 3). Thus, **12b** shows a photoinduced efficacy shift from partial agonist (*trans*) to antagonist (PSS_*cis*_) (Figure 3B,C).

Our studies reveal several noteworthy observations. First, it is striking that removing one chlorine atom (**12c**) or adding two more (**12d**) abolishes the switch in efficacy. Second, compared to **12b**, chloro-cyano substituted **12e** shows decreased binding affinity of both isomers and only a comparable antagonism for its PSS_*cis*_ state. We attribute this seemingly incoherent trend to the notion that adding an azobenzene to a β_2_-AR ligand has the potential to change SAR properties compared to those of the parent molecule (**18** in this case). Third, the molecular superposition (Figure 1C) reveals that the NH_2_ moiety of clenbuterol occupies a comparable position to the *para*-OH moiety of BI-167107, the latter of which is known to form H-bond interactions with Ser203 and Ser207 in the β_2_-AR binding pocket and to be key for agonism.^33^ Our azoextension strategy at this position necessarily removes the NH_2_ moiety, abolishing any interactions the NH_2_ group will have with the receptor. Indeed, the p*K*_i_ values for **12a**–**e** are all lower compared to that of clenbuterol. However, **12b** retains sufficient interaction with the receptor to partially activate β_2_-AR in its *trans* state, although the molecular mechanism of this agonism is unclear.

In recent years, the toolbox of photoswitchable β_2_-AR ligands has grown to encompass various antagonist and agonist series.^34^ A comparative analysis of reported photoswitchable β_2_-AR agonists reveals a complementary character of our series. Compound **12b** demonstrates an appreciable >25-fold increase in binding affinity upon *trans*–*cis* photoisomerization (Δp*K*_i_ > 1.4), accompanied by an efficacy switch from partial agonism to antagonism. “Photo-adrenaline”, in contrast, retains agonism in both its isomers.^17^ Moreover, following submission of the current work, the first photoswitchable covalent β_2_-AR agonist was disclosed.^35^ It exhibits a modest functional switch from antagonism to agonism upon illumination. In the current work, we report **12b** as the first noncovalent photoswitchable β_2_ ligand harboring an efficacy switch, i.e. switching from a partial agonist to *a* >25-fold higher affinity antagonist upon illumination. Ligand **12b** is a valuable addition to the growing toolbox of efficacy switches, the advantages of which were recently discussed in detail.^36^

## Conclusion

In all, we have synthesized and characterized five photoswitchable β_2_-AR ligands based on clenbuterol, indicating a successful azoextension strategy. All compounds can be photochemically interconverted between their isomeric forms, with sufficiently long thermal relaxation half-lives at 25 °C. Our set of compounds includes the *o*,*o*-dichloroazobenzene moiety, which has hitherto not frequently been studied as a photoswitchable unit. All compounds display a *cis*-on change in affinity and inhibitory potency upon illumination. Compound **12b** (VUF26034) was selected as a key compound due to its straightforward synthesis, suitable photochemical properties, and intriguing pharmacological properties. Upon *trans*–*cis* photoisomerization, **12b** displays *a* >25-fold increased binding affinity. Moreover, **12b** is the first reported noncovalent photoswitchable β_2_ agonist capable of achieving a switch in efficacy, from partial agonism in the dark to antagonism of the PSS_*cis*_ upon illumination. The dynamic isomerization of **12b** indicates its potential for studying the signaling and downstream signaling of β_2_-AR with high spatiotemporal precision.

## Experimental Section

All reagents have been purchased from commercial suppliers (primarily Sigma-Aldrich and Combi-Blocks) and used without further purification. Clenbuterol hydrochloride was purchased from Bosche Scientific (CAS: 21898-19-1, purity: 98%, Lot No.: BS2007087718). Propranolol had been synthesized in-house previously (VUF6102). Anhydrous THF, DCM, and DMF were dried by passing through an activated alumina column before use. All other solvents used were used as received unless otherwise stated. TLC analyses were performed using Screening Devices or Merck F254 aluminum-backed silica plates and visualized with 254 nm UV light. All reactions were carried out under N_2_ atmosphere (unless mentioned otherwise) and all reactions with photoresponsive compounds were carried out under dimmed or red light. LC–MS analysis was carried out on a Shimadzu LC-20AD liquid chromatograph pump system with a Shimadzu M20A photodiode array detector, a Shimadzu LCMS2010EV mass spectrometer and Xbridge C18 column (5 μm, 4.6 × 50 mm) at 40 °C using ESI in positive ion mode. For acidic runs, 0.1% formic acid in H_2_O and 0.1% formic acid in MeCN were used as eluent A and B, respectively. The gradient for acidic and basic runs was 5:90:90:5:5% B at *t* = 0:4.5:6:6.5:8 min. The purity of a compound was determined by calculating the peak area percentage of UV detection at 254 nm. Unless mentioned otherwise, all compounds are >95% pure by the HPLC-MS analysis listed above. HRMS spectra were determined with a Bruker micrOTOF mass spectrometer using ESI in positive ion mode. Reverse phase column chromatography was performed on Teledyne ISCO CombiFlash Rf 200 equipment with the same solvent systems used for LC–MS measurements. Normal phase flash chromatography was performed on Biotage Isolera. Prepacked columns were purchased from Screening Devices (C18 and UltraPure irregular silica) or BUCHI (FlashPure EcoFlex irregular silica). Nuclear magnetic resonance (NMR) spectra were determined with a Bruker Avance II 500 MHz or a Bruker Avance III HD 600 MHz spectrometer. Chemical shifts are reported in parts per million (ppm) against the reference compound using the signal of the residual nondeuterated solvent (CDCl_3_ δ = 7.26 ppm (^1^H), δ = 77.16 ppm (^13^C); DMSO-*d*_6_ δ = 2.50 ppm (^1^H), δ = 39.52 ppm (^13^C); CD_3_OD δ = 3.31 ppm (^1^H), δ = 49.00 ppm (^13^C)). NMR spectra were processed using MestReNova 14.0 software. The peak multiplicities are defined as follows: s, singlet; d, doublet; t, triplet; q, quartet; dd, doublet of doublets; ddd, doublet of doublets of doublets; dt, doublet of triplets; dq, doublet of quartets; td, triplet of doublets; tt, triplet of triplets; qd, quartet of doublets; p, pentet; dp, doublet of pentets; br, broad signal; m, multiplet. For NMR listings, in addition to specific instructions that are given by the journal in the guidelines for authors the following additional procedures were used: (1) Multiplicity is not solely reported based on peak shapes, but also distinguishes the coupling to all nonequivalent protons that have similar *J* values; (2) If additional smaller couplings are observed but are too small for accurate quantitation because the precision is smaller than the digital resolution, a symbol ^Δ^ will be used; (3) The notation “m” is used in case of obscured accurate interpretation as a result of (i) overlapping signals for different protons, or (ii) a result of overlapping signal lines within the same proton signal; (4) For any rotamers or diastereomers, signals will be listed separately if resolved; (5) NMR signals that could only be detected with HSQC analysis are denoted with a # symbol; (6) NMR signals that could only be detected with HMBC analysis are denoted with a * symbol; (7) If one or more signals remain undetected after extensive 1D and 2D NMR analyses, this will be mentioned. (8) Signals for exchangeable proton atoms (such as NH and OH groups) are only listed if clearly visible (e.g., excluding the use of D_2_O or CD_3_OD) and if confirmed by a D_2_O shake and/or HSQC.

### 2-(4-Nitrophenyl)­oxirane (**2**)

To an ice-cooled solution of 2-bromo-1-(4-nitrophenyl) ethan-1-one (**1**, 10.00 g, 40.98 mmol) in MeOH (250 mL), NaBH_4_ (1.55 g, 40.98 mmol) was added slowly. The solution was stirred for 2 h in the ice bath. K_2_CO_3_ (5.66 g, 40.98 mmol) was added, after which the ice bath was removed and stirring was continued at rt overnight. The solution was concentrated under reduced pressure. EtOAc (50 mL) and water (50 mL) were added. The layers were separated, and the aqueous layer was extracted with EtOAc (2 × 50 mL). The combined organic phases were washed once with brine (100 mL), dried over Na_2_SO_4,_ and concentrated *in vacuo*. This afforded a yellow oil (6.77 g), which was used in the next step without further purification.

### 2-(*tert*-Butylamino)-1-(4-nitrophenyl)­ethan-1-ol (**3**)

*tert*-Butylamine (21.5 mL, 205 mmol) was added to a solution of oxirane **2** (used directly from the previous step, 6.77 g) in EtOH (150 mL). The solution was stirred overnight at 75 °C. The reaction mixture was concentrated *in vacuo*. The residue was stirred with Et_2_O (20 mL) and the suspension was filtered. This process was repeated with the residue two more times. Drying of the residue under high vacuum afforded the title compound as a white solid (7.78 g, 80% for two steps from **1**). ^1^H NMR (500 MHz, DMSO-*d*_6_): δ 8.21–8.15 (m, 2H), 7.66–7.60 (m, 2H), 5.55 (s, 1H), 4.66 (dd, *J* = 7.7, 4.5 Hz, 1H), 2.65 (dd, *J* = 11.3, 4.5 Hz, 1H), 2.60 (dd, *J* = 11.3, 7.7 Hz, 1H), 1.42 (s, 1H), 0.99 (s, 9H). ^13^C NMR (151 MHz, DMSO-*d*_6_): δ 152.8, 146.4, 127.2, 123.1, 71.9, 50.3, 49.6, 28.9. LC–MS: *t*_R_ = 2.47 min, purity: 99.8%, *m*/*z* [M + H]^+^: 239.

### 1-(4-Aminophenyl)-2-(*tert*-butylamino)­ethan-1-ol (**4**)

A stirred solution of nitro-compound **3** (4.0 g, 16.8 mmol) in EtOH (80 mL) and 1.0 M HCl (10 mL) was purged with N_2_ for 5 min, after which Pd/C (5%, 1.03 g) was added. The suspension was placed under a H_2_ atmosphere using a balloon and stirred overnight at rt. The mixture was filtered over Celite which was washed with EtOH (50 mL). The filtrate was concentrated​ *in vacuo* to obtain an oil, which was diluted with EtOAc and satd. aq. Na_2_CO_3_. After extraction, the organic phase was dried (Na_2_SO_4_) and concentrated to obtain a yellow solid, which was triturated with EtOAc (2 × 20 mL). This gave the title compound as a white solid (2.3 g, 66%). ^1^H NMR (500 MHz, DMSO-*d*_6_): δ 7.00–6.94 (m, 2H), 6.54–6.47 (m, 2H), 4.90 (s, 2H), 4.86 (s, 1H), 4.31 (dd, *J* = 8.2, 4.5 Hz, 1H), 2.53 (dd, *J* = 11.1, 8.2 Hz, 1H), 2.50 (dd, *J* = 11.1, 4.5 Hz, 1H), 1.22 (s, 1H), 1.00 (s, 9H). ^13^C NMR (126 MHz, DMSO-*d*_6_): δ 147.5, 131.8, 126.6, 113.4, 72.4, 50.7, 49.4, 29.0. LC–MS: *t*_R_ = 0.78 min, purity: 99.2%, *m*/*z* [M + H]^+^: 209.

### (*E*)-2-(*tert*-Butylamino)-1-(4-(phenyldiazenyl)­phenyl)­ethan-1-ol (12a, VUF26044)

To a solution of aniline **4** (300 mg, 1.44 mmol) in DCM (30 mL) was added PhNO (309 mg, 2.88 mmol). After stirring at rt for 15 min, 1-adamantanecarboxylic acid (2.60 g, 14.40 mmol) was added. The solution was heated at 40 °C for 3 d. The mixture was diluted with EtOAc (20 mL) and water (20 mL). After extraction, the organic phase was washed with satd. aq. Na_2_CO_3_ (3 × 30 mL), water and brine. The organic phase was dried with anhydrous Na_2_SO_4_, filtered, and purified by flash column chromatography using DCM:MeOH 8:1 as eluent to yield the title compound as a yellow solid (0.14 g, 33%). ^1^H NMR (500 MHz, DMSO-*d*_6_): δ 7.90–7.87 (m, 2H), 7.87–7.84 (m, 2H), 7.62–7.54 (m, 5H), 5.39 (br s, 1H), 4.62 (dd, *J* = 8.1, 4.4 Hz, 1H), 2.67 (dd, *J* = 11.3, 4.4 Hz, 1H), 2.63 (dd, *J* = 11.3, 8.1 Hz, 1H), 1.43 (br s, 1H), 1.02 (s, 9H). ^13^C NMR (126 MHz, DMSO-*d*_6_): δ 152.0, 150.9, 148.6, 131.3, 129.4, 126.9, 122.5, 122.3, 72.2, 50.5, 49.6, 28.9. LC–MS: λ_max_: 322 nm, *t*_R_ = 3.39 min, purity: 99.8%, *m*/*z* [M + H]^+^: 298; HRMS calcd for C_18_H_24_N_3_O, [M + H]^+^ = 298.1914; found, 298.1898.

### 2-(*tert*-Butylamino)-1-(3,5-dichloro-4-nitrosophenyl)­ethan-1-ol (**5**)

To a suspension of clenbuterol hydrochloride (300 mg, 0.96 mmol) in DCM (5 mL) was added a solution of Oxone (0.59 g, 1.92 mmol) in water (20 mL). The reaction mixture was stirred at rt for 2 h. EtOAc (15 mL), water (20 mL) and satd. aq. Na_2_CO_3_ (10 mL) were added. The organic layer was separated, and the aqueous phase was extracted with EtOAc (2 × 20 mL). The combined organic phases were washed with brine, dried over anhydrous Na_2_SO_4_, filtered, and concentrated *in vacuo*. This yielded a green oil (0.25 g), which was used in the next step without further purification.

### (*E*)-2-(*tert*-Butylamino)-1-(3,5-dichloro-4-(phenyldiazenyl) phenyl)­ethan-1-ol (12b, VUF26034)

To a solution of PhNH_2_ (0.12 g, 1.29 mmol) in DCM (30 mL) was added nitroso-compound **5** (used directly from the previous step, 0.25 g). After stirring at rt for 15 min, 1-adamantanecarboxylic acid (1.55 g, 8.59 mmol) was added. The reaction mixture was heated at 40 °C for overnight. The mixture was added to EtOAc (30 mL) and water (30 mL). After extraction, the organic phase was washed with satd. aq. Na_2_CO_3_ (3 × 30 mL), water and brine. The organic phase was dried with anhydrous Na_2_SO_4_, filtered, and purified by flash column chromatography using DCM:MeOH 13:1 as eluent to yield the title compound as a yellow solid (75 mg, 24% for two steps from clenbuterol hydrochloride). ^1^H NMR (600 MHz, DMSO-*d*_6_): δ 7.93–7.90 (m, 2H), 7.70–7.66 (m, 2H), 7.66–7.64 (m, 1H), 7.61 (s, 2H), 5.58 (br s, 1H), 4.61 (dd, *J* = 7.4, 4.7 Hz, 1H), 2.70 (dd, *J* = 11.4, 4.7 Hz, 1H), 2.66 (dd, *J* = 11.4, 7.4 Hz, 1H), 1.02 (s, 9H). The peak at 5.58 ppm disappears upon a D_2_O shake. ^13^C NMR (126 MHz, CD_3_OD): δ 153.8, 148.7, 146.9, 133.7, 130.5, 127.8, 127.6, 123.9, 72.6, 51.7, 51.0, 28.7. LC–MS: λ_max_: 302 nm, *t*_R_ = 3.70 min, purity: 98.9%, *m*/*z* [M + H]^+^: 366. HRMS calcd for C_18_H_22_Cl_2_N_3_O, [M + H]^+^ = 366.1134; found, 366.1132.

### Methyl 4-amino-3-bromo-5-chlorobenzoate (**6f**)

Methyl 4-amino-3-chlorobenzoate (**6c**, 3.02 g, 16.16 mmol) and PhMe (60 mL) were added to a round-bottom flask equipped with a reflux condenser. NBS (2.88 g, 16.16 mmol) was added. Stirring was continued for 10 min at 35 °C. The solution was washed with 5% aq. NaHCO_3_ (50 mL) and water (50 mL). The organic phase was washed with brine, dried over anhydrous Na_2_SO_4_, filtered, and concentrated *in vacuo.* Removal of the solvent under vacuum yielded a dark brown oil, which was purified by flash column chromatography using *c*-Hex:EtOAc 13:1 as eluent to yield the title compound as a white solid (3.8 g, 89%). ^1^H NMR (600 MHz, DMSO-*d*_6_): δ 7.87 (d, *J* = 1.9 Hz, 1H), 7.75 (d, *J* = 1.9 Hz, 1H), 6.32 (s, 2H), 3.78 (s, 3H). ^13^C NMR (151 MHz, DMSO-*d*_6_): δ 164.3, 146.4, 132.4, 129.6, 117.9, 116.9, 106.7, 52.0.

### Methyl 3-bromo-5-chloro-4-nitrosobenzoate (**7f**)

To a solution of aniline **6f** (2.03 g, 7.56 mmol) in DCM (50 mL) in an ice bath was slowly added *m*-CPBA (5.93 g, 26.47 mmol). The ice bath was removed, and the reaction mixture was allowed to warm to rt. After 36 h, the reaction mixture was diluted with DCM (40 mL), and satd. aq. Na_2_S_2_O_3_ (20 mL) and satd. aq. NaHCO_3_ (20 mL) were added. The resulting suspension was filtered. The organic phase was separated and washed with water and brine. The organic phase was dried over Na_2_SO_4_, filtered, and concentrated *in vacuo* to yield the title compound as a green oil (1.90 g), which was used in the next step without further purification.

### Methyl (*E*)-3-bromo-5-chloro-4-(phenyldiazenyl)­benzoate (**8f**)

To a solution of nitroso-compound **7f** (used directly from the previous step, 1.90 g) in DCM (60 mL) was added PhNH_2_ (0.62 mL, 6.81 mmol) and AcOH (5.0 mL, 12.85 mmol). The reaction mixture was stirred at rt overnight. Extraction was performed with EtOAc (30 mL) and water (40 mL). The aqueous phase was extracted with EtOAc (2 × 30 mL). The combined organic phases were washed with satd. aq. NaHCO_3_ (3 × 30 mL), water (3 × 30 mL) and brine. The organic phase was dried with anhydrous Na_2_SO_4_, filtered, and purified by flash column chromatography using *c*-Hex:EtOAc 19:1 as eluent to yield the title compound as a red solid (0.28 g, 12% for two steps from **6f**). ^1^H NMR (500 MHz, DMSO-*d*_6_): δ 8.23 (d, *J* = 1.6 Hz, 1H), 8.12 (d, *J* = 1.6 Hz, 1H), 7.98–7.96 (m, 1H), 7.96–7.94 (m, 1H), 7.74–7.66 (m, 3H), 3.91 (s, 3H). ^13^C NMR (151 MHz, DMSO-*d*_6_): δ 163.6, 152.1, 151.5, 133.7, 132.7, 130.8, 130.4, 129.9, 125.0, 123.0, 115.0, 53.0. LC–MS: *t*_R_ = 5.87 min, purity: 99.9%, *m*/*z* not found.

### (*E*)-(3-Bromo-5-chloro-4-(phenyldiazenyl)­phenyl)­methanol (**9f**)

A mixture of ester **8f** (1.78 g, 5.03 mmol) and THF (80 mL) was cooled in an ice bath, after which a DIBAL-H solution (1.0 M in *c-*Hex, 15.1 mL, 15.1 mmol) was added slowly. The reaction mixture was warmed slowly to rt and stirred for 2 h. The reaction mixture was diluted with EtOAc (20 mL) and quenched with satd. aq. potassium sodium tartrate (30 mL) while cooling in an ice bath. Additional water (20 mL) was added, and the resulting mixture was stirred until it became clear (typically: 1–2 h). The layers were separated and the organic phase was washed with brine, dried over Na_2_SO_4_, and filtered. The solvent was removed *in vacuo*. The residue was purified by flash column chromatography using *c*-Hex:EtOAc 8:1 as eluent to yield the title compound as a red solid (1.45 g, 88%). ^1^H NMR (600 MHz, DMSO-*d*_6_): δ 7.94–7.91 (m, 2H), 7.72 (d, *J* = 1.7 Hz, 1H), 7.70–7.64 (m, 3H), 7.59 (d, *J* = 1.7 Hz, 1H), 5.55 (t, *J* = 5.8 Hz, 1H), 4.57 (d, *J* = 5.8 Hz, 2H). ^13^C NMR (151 MHz, DMSO-*d*_6_): δ 151.6, 146.9, 145.4, 133.0, 129.8, 129.7, 127.5, 124.6, 122.7, 114.9, 61.2. LC–MS: *t*_R_ = 4.91 min, purity: 98.9%, *m*/*z* [M + H]^+^: 325.

### (*E*)-3-Chloro-5-(hydroxymethyl)-2-(phenyldiazenyl)­benzonitrile (**9e**)

This procedure was based on two literature description.^27,28^ Zn­(CN)_2_ (0.16 g, 1.38 mmol) and Pd­(PPh_3_)_4_ (0.32 g, 0.28 mmol) were added to a round-bottomed flask. The flask was evacuated and backfilled with N_2_. A solution of bromide **9f** (0.90 g, 2.76 mmol) in DMF (30 mL) was added. The reaction mixture was stirred at 100 °C for 3 h. The reaction mixture was cooled to rt, diluted with EtOAc (30 mL) and filtered. The residue was washed with EtOAc (20 mL). To the filtrate was added water (30 mL) and extraction was performed with EtOAc (2 × 30 mL). The combined organic phases were washed with water and brine, dried over anhydrous Na_2_SO_4_, concentrated under reduced pressure and purified by flash column chromatography using *c*-Hex:EtOAc 3:1 as eluent to yield the title compound as a pink solid (0.35 g, 47%). ^1^H NMR (600 MHz, DMSO-*d*_6_): δ 7.97–7.96 (m, 1H), 7.96–7.94 (m, 2H), 7.89–7.87 (m, 1H), 7.72–7.66 (m, 3H), 5.67 (t, *J* = 4.9 Hz, 1H), 4.63 (d, *J* = 4.9 Hz, 2H). ^13^C NMR (151 MHz, DMSO-*d*_6_): δ 151.4, 148.4, 147.1, 133.5, 132.6, 131.7, 131.3, 129.9, 123.1, 116.6, 102.5, 61.1. LC–MS: *t*_R_ = 4.55 min, purity: 96.2%, *m*/*z* [M + H]^+^: 272.

### (*E*)-3-Chloro-5-formyl-2-(phenyldiazenyl)­benzonitrile (**10e**)

To a solution of alcohol **9e** (0.37 g, 1.36 mmol) in DCM (30 mL) in an ice bath was added Dess–Martin reagent (0.87 g, 2.04 mmol). The reaction mixture was stirred at rt for 1 h. The reaction mixture was quenched with satd. aq. Na_2_SO_3_ (20 mL) and satd. aq. NaHCO_3_ (15 mL). The aqueous phase was extracted with DCM (2 × 30 mL). The combined organic phases were washed with water and brine, dried over anhydrous Na_2_SO_4_, filtered, concentrated, and purified by flash column chromatography using *c*-Hex:EtOAc 5.6:1 as eluent to obtain a red solid (0.33 g, 90%). ^1^H NMR (600 MHz, DMSO-*d*_6_): δ 10.06 (s, 1H), 8.50 (d, *J* = 1.6 Hz, 1H), 8.48 (d, *J* = 1.6 Hz, 1H), 8.03–7.99 (m, 2H), 7.78–7.74 (m, 1H), 7.73–7.69 (m, 2H). ^13^C NMR (151 MHz, DMSO-*d*_6_): δ 190.2, 153.4, 151.5, 137.2, 135.2, 134.6, 134.4, 131.6, 130.1, 123.4, 115.5, 103.8. LC–MS: *t*_R_ = 5.01 min, purity: 95.5%, *m*/*z* [M + H]^+^: 270.

### (*E*)-3-Chloro-5-(oxiran-2-yl)-2-(phenyldiazenyl)­benzonitrile (**11e**)

A mixture of NaH (23 mg, 0.58 mmol) and DMSO (30 mL) was cooled to 0 °C. Trimethylsulfonium iodide (0.12 g, 0.58 mmol) was added. The mixture was warmed to rt and stirred for 1 h. THF (10 mL) was added. After stirring for 1 h, the mixture was cooled in an ice bath, and aldehyde **10e** (0.13 g, 0.48 mmol) was added slowly. The mixture was stirred at rt for 1 h. The reaction mixture was diluted with EtOAc (30 mL) and water (40 mL). Extraction was performed. The organic phase was washed with brine, dried over anhydrous Na_2_SO_4_, filtered, concentrated, and purified by flash column chromatography using *c*-Hex:EtOAc 5:1 as eluent to yield the title compound as a red solid (11 mg, 8%). ^1^H NMR (600 MHz, CD_3_OD): δ 8.02–7.99 (m, 2H), 7.81 (d, *J* = 1.8 Hz, 1H), 7.74 (d, *J* = 1.8 Hz, 1H), 7.65–7.59 (m, 3H), 4.02 (dd, *J* = 4.1, 2.5 Hz, 1H), 3.21 (dd, *J* = 5.4, 4.1 Hz, 1H), 2.86 (dd, *J* = 5.4, 2.5 Hz, 1H). ^13^C NMR (151 MHz, CD_3_OD): δ 153.4, 151.4, 143.6, 134.4, 134.4, 133.1, 131.8, 130.6, 124.5, 117.4, 104.8, 52.2, 51.4. LC–MS: *t*_R_ = 5.13 min, purity: 96.7%, *m*/*z* [M + H]^+^: 284.

### (*E*)-5-(2-(*tert*-Butylamino)-1-hydroxyethyl)-3-chloro-2-(phenyldiazenyl)­benzonitrile (**12e**, VUF26202)

To a mixture of epoxide **11e** (11 mg, 0.04 mmol) in EtOH (20 mL) was added *tert*-butylamine (0.14 mL, 1.3 mmol). The reaction mixture was stirred at 75 °C overnight. The solvent was removed *in vacuo*. Extraction was performed with water (20 mL) and EtOAc (10 mL). The aqueous phase was extracted with EtOAc (2 × 15 mL). The combined organic phases were washed with water and brine, dried over Na_2_SO_4_, filtered, concentrated, and purified by flash column chromatography using DCM:MeOH 5:1 as eluent to yield the title compound as a red solid (8 mg, 58%). ^1^H NMR (600 MHz, CD_3_OD): δ 8.03–8.00 (m, 2H), 7.99 (d, *J* = 1.8 Hz, 1H), 7.90 (d, *J* = 1.8 Hz, 1H), 7.66–7.60 (m, 3H), 4.92 (dd, *J* = 9.5, 3.4 Hz, 1H), 3.04 (dd, *J* = 12.0, 3.4 Hz, 1H), 2.93 (dd, *J* = 12.0, 9.5 Hz, 1H), 1.27 (s, 9H). ^13^C NMR (151 MHz, CD_3_OD): δ 153.5, 151.3, 147.7, 134.4, 134.2, 133.8, 132.3, 130.6, 124.4, 117.5, 104.6, 70.9, 53.5*, 49.9, 27.3. *HMBC analysis indicates the presence of an additional quaternary carbon at 53.5 ppm. LC–MS: λ_max_: 325 nm, *t*_R_ = 3.55 min, purity: 98.9%, *m*/*z* [M + H]^+^: 357. HRMS calcd for C_19_H_22_ClN_4_O, [M + H]^+^ = 357.1477; found, 357.1470.

### Methyl 3-chloro-4-nitrosobenzoate (**7c**)

To a solution of methyl 4-amino-3-chlorobenzoate **6c** (1.01 g, 5.39 mmol) in DCM (5 mL) was added Oxone (6.62 g, 10.78 mmol) in water (20 mL). The reaction mixture was stirred at rt for 36 h. Water was added and the layers were separated. The aqueous phase was extracted with DCM (2 × 20 mL). The combined organic phases were washed with 1 M HCl, water and brine, dried over anhydrous Na_2_SO_4_, filtered, and concentrated *in vacuo* to yield a green oil (1.1 g), which was used in the next step without further purification.

### Methyl (*E*)-3-chloro-4-(phenyldiazenyl)­benzoate (**8c**)

To a solution of nitroso-compound **7c** (used directly from the previous step, 1.1 g) in DCM (40 mL) were added PhNH_2_ (0.50 g, 5.39 mmol) and AcOH (6.2 mL, 107.76 mmol). The reaction mixture was stirred at rt for 2 h. To the mixture was added water (30 mL) and the layers were separated. The aqueous phase was extracted with EtOAc (2 × 30 mL). The combined organic phases were washed with satd. aq. Na_2_CO_3_ (3 × 30 mL), water and brine, dried with anhydrous Na_2_SO_4_, filtered, and purified by flash column chromatography using *c*-Hex:EtOAc 9:1 as eluent to yield the title compound as a yellow solid (0.58 g, 39% for two steps from **6c**). ^1^H NMR (600 MHz, DMSO-*d*_6_): δ 8.17 (d, *J* = 1.8 Hz, 1H), 8.03 (dd, *J* = 8.4, 1.8 Hz, 1H), 7.97–7.94 (m, 2H), 7.74 (d, *J* = 8.4 Hz, 1H), 7.67–7.63 (m, 3H), 3.91 (s, 3H). ^13^C NMR (151 MHz, DMSO-*d*_6_): δ 164.6, 152.1, 150.6, 133.6, 133.0, 132.7, 131.3, 129.8, 129.0, 123.3, 118.2, 52.8. LC–MS: *t*_R_ = 5.78 min, purity: 99.6%, *m*/*z* [M + H]^+^: 275.

### (*E*)-(3-Chloro-4-(phenyldiazenyl)­phenyl)­methanol (**9c**)

Ester **8c** (0.36 g, 1.31 mmol) was dissolved in THF (40 mL) after which a DIBAL-H solution (1.0 M in *c*-Hex, 6.60 mL, 6.60 mmol) was added slowly in an ice bath. The reaction mixture was warmed slowly to rt and stirred for 2 h. The reaction mixture was diluted with EtOAc (20 mL) and quenched with satd. aq. potassium sodium tartrate (30 mL) whiling cooling in an ice bath. Additional water (10 mL) was added, and the resulting mixture was stirred at rt until it became clear (typically: 1–2 h). The layers were separated. The aqueous phase was extracted with EtOAc (2 × 30 mL). The combined organic phases were washed with brine, dried over Na_2_SO_4_, filtered, and the solvent was removed *in vacuo*. The residue was triturated with *c*-Hex (15 mL) to obtain the title compound as a yellow solid (0.45 g, 86%). ^1^H NMR (600 MHz, DMSO-*d*_6_): δ 7.94–7.90 (m, 2H), 7.69–7.67 (m, 1H), 7.65–7.59 (m, 4H), 7.44–7.41 (m, 1H), 5.51 (t, *J* = 5.5 Hz, 1H), 4.60 (d, *J* = 5.5 Hz, 2H). ^13^C NMR (151 MHz, DMSO-*d*_6_): δ 152.1, 148.3, 146.5, 134.2, 132.1, 129.6, 128.0, 125.7, 122.9, 117.2, 61.8. LC–MS: *t*_R_ = 4.77 min, purity: 91.9%, *m*/*z* [M + H]^+^: 247.

### (*E*)-3-Chloro-4-(phenyldiazenyl)­benzaldehyde (**10c**)

To a solution of alcohol **9c** (100 mg, 0.41 mmol) in DCM (20 mL) in an ice bath was added Dess–Martin reagent (0.26 g, 0.61 mmol). The reaction mixture was stirred at rt for 1 h. After completion, the reaction mixture was quenched with saturated aqueous Na_2_SO_3_ (10 mL) and satd. aq. NaHCO_3_ (10 mL). The aqueous phase was extracted with EtOAc (2 × 20 mL). The combined organic phases were washed with water and brine, dried over anhydrous Na_2_SO_4_, filtered, and concentrated *in vacuo* to obtain a red solid (85 mg, 86%). ^1^H NMR (600 MHz, DMSO-*d*_6_): δ 10.08 (s, 1H), 8.25–8.23 (m, 1H), 8.02–7.96 (m, 3H), 7.82–7.79 (m, 1H), 7.68–7.65 (m, 3H). ^13^C NMR (151 MHz, DMSO-*d*_6_): δ 191.8, 152.2, 151.1, 138.5, 134.1, 133.1, 132.0, 129.8, 128.6, 123.3, 118.7. LC–MS: *t*_R_ = 5.39 min, purity: 95.5%, *m*/*z* not found.

### (*E*)-1-(2-Chloro-4-(oxiran-2-yl)­phenyl)-2-phenyldiazene (**11c**)

A mixture of NaH (8.2 mg, 0.20 mmol) in DMSO/THF = 1/1 (30 mL total) was cooled to 0 °C. Trimethylsulfonium iodide (41.7 mg, 0.20 mmol) was added. The mixture was warmed to rt and stirred for 1 h. Benzaldehyde **10c** (50 mg, 0.20 mmol) was added slowly while cooling in an ice bath. The mixture was stirred at rt for 1 h. After completion, the reaction mixture was diluted with EtOAc (10 mL). Water (15 mL) was added and the layers were separated. The aqueous phase was extracted with EtOAc (2 × 20 mL). The combined organic phases were washed with brine, dried over anhydrous Na_2_SO_4_, filtered, concentrated *in vacuo*, and purified by flash column chromatography using *c*-Hex:EtOAc 9:1 as eluent to yield the title compound as a yellow solid (48 mg, 91%). ^1^H NMR (600 MHz, DMSO-*d*_6_): δ 7.94–7.91 (m, 2H), 7.70–7.68 (m, 1H), 7.66–7.61 (m, 4H), 7.43–7.40 (m, 1H), 4.08 (dd, *J* = 4.2, 2.5 Hz, 1H), 3.20 (dd, *J* = 5.3, 4.2 Hz, 1H), 2.96 (dd, *J* = 5.3, 2.5 Hz, 1H). ^13^C NMR (151 MHz, DMSO-*d*_6_): δ 152.1, 147.5, 143.2, 134.3, 132.3, 129.6, 127.9, 125.4, 123.0, 117.7, 50.9, 50.6. LC–MS: *t*_R_ = 5.49 min, purity: 89.2%, *m*/*z* not found.

### (*E*)-2-(*tert*-Butylamino)-1-(3-chloro-4-(phenyldiazenyl) phenyl)­ethan-1-ol (**12c**, VUF26125)

To a mixture of epoxide **11c** (48 mg, 0.19 mmol) in EtOH (10 mL) was added *tert*-butylamine (0.39 mL, 3.71 mmol). The reaction mixture was stirred at 75 °C overnight. After completion, the solvent was removed *in vacuo*. The residue was purified by flash column chromatography using DCM:MeOH 6:1 as eluent to yield the title compound as a yellow solid (29 mg, 47%). ^1^H NMR (500 MHz, CD_3_OD): δ 7.98–7.93 (m, 2H), 7.72 (d, *J* = 8.3 Hz, 1H), 7.67 (d, *J* = 1.7 Hz, 1H), 7.59–7.52 (m, 3H), 7.43 (dd, *J* = 8.3, 1.7 Hz, 1H), 4.79 (dd, *J* = 8.6, 4.3 Hz, 1H), 2.82 (dd, *J* = 11.9, 4.3 Hz, 1H), 2.78 (dd, *J* = 11.9, 8.6 Hz, 1H), 1.18 (s, 9H). ^13^C NMR (151 MHz, CD_3_OD): δ 154.2, 149.6, 149.1, 136.6, 132.9, 130.4, 129.2, 126.3, 124.2, 118.5, 72.8, 52.3, 50.9, 28.4. LC–MS: λ_max_: 327 nm, *t*_R_ = 3.65 min, purity: 98.5%, *m*/*z* [M + H]^+^: 332. HRMS calcd for C_18_H_23_ClN_3_O, [M + H]^+^ = 332.1524; found, 332.1520.

### Methyl 4-nitrosobenzoate (**7a**)

To a solution of methyl 4-aminobenzoate **6a** (4.04 g, 26.46 mmol) in DCM (30 mL) was added Oxone (16.27 g, 52.92 mmol) in water (120 mL). The reaction mixture was stirred at rt overnight. Water (30 mL) was added and the layers were separated. The aqueous phase was extracted with DCM (2 × 40 mL). The combined organic phases were washed with 1.0 M HCl (30 mL), water and brine, dried over anhydrous Na_2_SO_4_, filtered, and concentrated *in vacuo* to yield a green oil (4.32 g), which was used in the next step without further purification.

### Methyl (*E*)-4-(phenyldiazenyl)­benzoate (**8a**)

To a solution of nitroso-compound **7a** (used directly from the previous step, 4.32 g) in DCM (80 mL) were added PhNH_2_ (2.46 g, 26.46 mmol) and AcOH (15.10 mL, 264.61 mmol). The reaction mixture was stirred at rt for 2 h. Water (40 mL) was added and the layers were separated. The aqueous phase was extracted with EtOAc (2 × 40 mL). The combined organic phases were washed with satd. aq. Na_2_CO_3_ (3 × 30 mL), water and brine, dried with anhydrous Na_2_SO_4_, filtered, and purified by flash column chromatography using *c*-Hex:EtOAc 19:1 as eluent to yield the title compound as a red solid (3.48 g, 55% for two steps from **6a**). ^1^H NMR (600 MHz, DMSO-*d*_6_): δ 8.19–8.16 (m, 2H), 8.01–7.98 (m, 2H), 7.96–7.93 (m, 2H), 7.65–7.62 (m, 3H), 3.90 (s, 3H). ^13^C NMR (151 MHz, DMSO-*d*_6_): δ 165.6, 154.4, 151.9, 132.4, 131.6, 130.6, 129.6, 122.9, 122.7, 52.5. LC–MS: *t*_R_ = 5.44 min, purity: 99.1%, *m*/*z* [M + H]^+^: 241.

### (*E*)-(4-(Phenyldiazenyl)­phenyl)­methanol (**9a**)

Ester **8a** (3.48 g, 14.48 mmol) was dissolved in THF (80 mL) after which a DIBAL-H solution (1.0 M in *c*-Hex, 43.5 mL, 43.5 mmol) was added slowly while cooling in an ice bath. The reaction mixture was warmed slowly to rt and stirred for 2 h. The reaction mixture was diluted with EtOAc (60 mL) and quenched with satd. aq. potassium sodium tartrate (30 mL) while cooling in an ice bath. Additional water (20 mL) was added, and the resulting mixture was stirred at rt until it became clear (typically: 1–2 h). The layers were separated. The aqueous phase was extracted with EtOAc (2 × 50 mL). The combined organic phases were washed with brine, dried over Na_2_SO_4_, filtered, and the solvent was removed *in vacuo*. The residue was purified by flash column chromatography using *c*-Hex:EtOAc 4:1 as eluent to yield the title compound as a red solid (2.7 g, 88%). ^1^H NMR (600 MHz, DMSO-*d*_6_): δ 7.90–7.86 (m, 4H), 7.62–7.58 (m, 2H), 7.58–7.56 (m, 1H), 7.55–7.52 (m, 2H), 5.40 (t, *J* = 5.7 Hz, 1H), 4.61 (d, *J* = 5.7 Hz, 2H).^13^C NMR (151 MHz, DMSO-*d*_6_): δ 151.9, 150.8, 146.5, 131.4, 129.5, 127.2, 122.5, 62.5. LC–MS: *t*_R_ = 4.41 min, purity: 94.3%, *m*/*z* [M + H]^+^: 213.

### (*E*)-4-(Phenyldiazenyl)­benzaldehyde (**10a**)

To a solution of alcohol **9a** (2.7 g, 12.72 mmol) in DCM (60 mL) in an ice bath was added Dess-Martin reagent (8.1 g, 19.08 mmol). The reaction mixture was stirred at rt for 1 h. After completion, the reaction mixture was quenched with satd. aq. Na_2_SO_3_ (30 mL) and satd. aq. NaHCO_3_ (30 mL). The aqueous phase was extracted with EtOAc (2 × 50 mL). The combined organic phases were washed with water and brine, dried over anhydrous Na_2_SO_4_, filtered and concentrated. The residue was purified by flash column chromatography using *c*-Hex:EtOAc 13:1 as eluent to obtain a yellow solid (2.28 g, 85%). ^1^H NMR (600 MHz, DMSO-*d*_6_): δ 10.12 (s, 1H), 8.15–8.12 (m, 2H), 8.08–8.05 (m, 2H), 7.97–7.94 (m, 2H), 7.66–7.62 (m, 3H). ^13^C NMR (151 MHz, DMSO-*d*_6_): δ 192.8, 155.0, 151.9, 137.6, 132.5, 130.9, 129.7, 123.1, 123.0. LC–MS: *t*_R_ = 5.06 min, purity: 99.9%, *m*/*z* [M + H]^+^: 211.

### (*E*)-3,5-Dichloro-4-((2,6-dichlorophenyl)­diazenyl)­benzaldehyde (**10d**)

This procedure was based on two literature description.^29,30^ Aldehyde **10a** (1.61 g, 7.61 mmol), NCS (5.08 g, 38.05 mmol) and Pd­(OAc)_2_ (0.17 g, 0.76 mmol) were dissolved in AcOH (100 mL). The reaction mixture was heated at 140 °C for 13 h during which time the color turned dark red. After cooling to rt, the solvent was removed under reduced pressure. Extraction was performed with water (30 mL) and EtOAc (30 mL). The aqueous phase was extracted with EtOAc (2 × 30 mL). The combined organic phases were washed with water (3 × 30 mL) and brine, dried over Na_2_SO_4_, filtered, concentrated, and purified by flash column chromatography using *c*-Hex:EtOAc 19:1 as eluent to obtain a red solid (1.3 g, 49%). ^1^H NMR (600 MHz, DMSO-*d*_6_): δ 10.03 (s, 1H), 8.20 (s, 2H), 7.75 (d, *J* = 8.1 Hz, 2H), 7.58 (t, *J* = 8.1 Hz, 1H). ^13^C NMR (151 MHz, DMSO-*d*_6_): δ 190.5, 150.2, 146.1, 137.2, 132.0, 130.4, 130.2, 126.5, 126.4. LC–MS: *t*_R_ = 5.70 min, purity: 77.4%, *m*/*z* not found.

### (*E*)-1-(2,6-Dichloro-4-(oxiran-2-yl)­phenyl)-2-(2,6-dichlorophenyl)­diazene (**11d**)

A mixture of NaH (0.21 g, 5.17 mmol) in DMSO (30 mL) was cooled to 0 °C. Trimethylsulfonium iodide (1.06 g, 5.17 mmol) was added. The mixture was warmed to rt and stirred for 1 h. THF (10 mL) was added. After stirring for 1 h, benzaldehyde **10d** (1.50 g, 4.31 mmol) was added slowly while cooling in an ice bath. The mixture was stirred at rt for 1 h. After completion, the reaction mixture was diluted with EtOAc (20 mL). Water (30 mL) was added and the layers were separated. The aqueous phase was extracted with EtOAc (2 × 30 mL). The combined organic phases were washed with brine, dried over anhydrous Na_2_SO_4_, filtered, and concentrated. The residue was purified by flash column chromatography using *c*-Hex:EtOAc 19:1 as eluent to yield the title compound as a red solid (0.47 g, 30%). ^1^H NMR (600 MHz, DMSO-*d*_6_): δ 7.71–7.68 (m, 2H), 7.62 (s, 2H), 7.53–7.50 (m, 1H), 4.09 (dd, *J* = 4.2, 2.5 Hz, 1H), 3.20 (dd, *J* = 5.4, 4.2 Hz, 1H), 2.99 (dd, *J* = 5.4, 2.5 Hz, 1H). ^13^C NMR (151 MHz, DMSO-*d*_6_): δ 146.6, 145.8, 142.1, 131.3, 130.0, 127.1, 126.5, 126.0, 50.8, 50.2. LC–MS: *t*_R_ = 5.76 min, purity: 99.1%, *m*/*z* not found.

### (*E*)-2-(*tert*-Butylamino)-1-(3,5-dichloro-4-((2,6-dichlorophenyl)­diazenyl)­phenyl)­ethan-1-ol (**12d**, VUF26175)

To a mixture of epoxide **11d** (0.23 g, 0.64 mmol) in EtOH (30 mL) was added *tert*-butylamine (0.70 mL, 6.66 mmol). The reaction mixture was stirred at 75 °C overnight. After completion, the solvent was removed *in vacuo*. Water (20 mL) and EtOAc (30 mL) were added. The layers were separated. The aqueous phase was extracted with EtOAc (2 × 30 mL). The combined organic phases were washed with water and brine, dried over Na_2_SO_4_, filtered and concentrated. The residue was purified by flash column chromatography using DCM:MeOH 5:1 as eluent to yield the title compound as a red solid (0.13 g, 45%). ^1^H NMR (600 MHz, CD_3_OD): δ 7.63 (s, 2H), 7.58 (d, *J* = 8.1 Hz, 2H), 7.40 (t, *J* = 8.1 Hz, 1H), 4.83–4.79 (m, 1H), 2.96–2.87 (m, 1H), 2.87–2.78 (m, 1H), 1.22 (s, 9H). ^13^C NMR (151 MHz, CD_3_OD): δ 148.8, 148.0, 147.6, 131.3, 130.7, 128.4, 128.2, 128.1, 72.0, 52.9, 50.6, 28.2. LC–MS: λ_max_: 290 nm, *t*_R_ = 3.91 min, purity: 97.7%, *m*/*z* [M + H]^+^: 436. HRMS calcd for C_18_H_20_Cl_4_N_3_O, [M + H]^+^ = 434.0355; found, 434.0357.

### 1-(4-Amino-3-bromophenyl)­ethan-1-one (**14**)

1-(4-aminophenyl)­ethan-1-one (**13,** 3.0 g, 22.1 mmol) and PhMe (60 mL) were added to a round-bottom flask equipped with a reflux condenser. NBS (4.0 g, 22.1 mmol) was added. Stirring was continued for 10 min at 35 °C. EtOAc (20 mL) and water (50 mL) were added. The layers were separated. The aqueous phase was extracted with EtOAc (20 mL). The combined organic layers were successively washed with of aq. 5% NaHCO_3_. Removal of the solvent *in* *vacuo* yielded a dark brown oil, which was purified with flash column chromatography using *c*-Hex:EtOAc 5:1 as eluent to yield the title compound as a yellow oil (3.6 g, 75%). ^1^H NMR (500 MHz, DMSO-*d*_6_): δ 7.93 (d, *J* = 2.0 Hz, 1H), 7.68 (dd, *J* = 8.5, 2.0 Hz, 1H), 6.80 (d, *J* = 8.5 Hz, 1H), 6.21 (s, 2H), 2.41 (s, 3H). ^13^C NMR (126 MHz, DMSO-*d*_6_): δ 194.9, 150.8, 133.8, 129.8, 126.9, 114.4, 106.7, 26.4. LC–MS: *t*_R_ = 3.49 min, purity: 97.2%, *m*/*z* [M + H]^+^: 214.

### 5-Acetyl-2-aminobenzonitrile (**15**)

To a solution of bromide **15** (3.6 g, 16.7 mmol) in DMF (60 mL) was added CuCN (1.8 g, 20.0 mmol). The reaction mixture was stirred at 160 °C for 6 h. The resulting mixture was cooled to rt and treated with a solution of FeCl_3_ (prepared from 6.6 g FeCl_3_, 6.6 mL HCl, and 20 mL water). The mixture was stirred for 30 min at rt and poured into water, followed by extraction with DCM (2 × 30 mL). The combined organic layers were washed with water (3 × 30 mL) and brine. The organic layer was dried over anhydrous Na_2_SO_4_ and filtered. The solvent was removed​ *in* *vacuo* to obtain a brown oil, which was purified by flash column chromatography using *c*-Hex:EtOAc 4:1 as eluent to yield the title compound as a yellow solid (1.5 g, 56%). ^1^H NMR (500 MHz, DMSO-*d*_6_): δ 8.06 (d, *J* = 2.1 Hz, 1H), 7.84 (dd, *J* = 8.9, 2.1 Hz, 1H), 6.91 (s, 2H), 6.81 (d, *J* = 8.9 Hz, 1H), 2.43 (s, 3H). ^13^C NMR (126 MHz, DMSO-*d*_6_): δ 194.4, 154.8, 135.2, 133.5, 125.1, 117.3, 114.7, 92.5, 25.9. LC–MS: *t*_R_ = 2.95 min, purity: 97.8%, *m*/*z* [M + H]^+^:161.

### 5-Acetyl-2-amino-3-chlorobenzonitrile (**16**)

To a stirred solution of aniline **15** (0.2 g, 1.25 mmol) in MeCN (20 mL) was added NCS (0.2 g, 1.5 mmol) at room temperature. Stirring was continued at 60 °C for 3 h. EtOAc (20 mL) and water (30 mL) were added. The layers were separated. The aqueous phase was extracted with EtOAc (20 mL). The combined organic layers were washed with brine, dried over anhydrous Na_2_SO_4_, and filtered. The solvent was removed *in* *vacuo* to obtain a brown oil, which was purified by flash column chromatography using *c*-Hex:EtOAc 4:1 as eluent to yield the title compound as a yellow solid (0.1 g, 41%). ^1^H NMR (500 MHz, DMSO-*d*_6_): δ 8.10 (d, *J* = 2.0 Hz, 1H), 7.99 (d, *J* = 2.0 Hz, 1H), 7.08 (s, 2H), 2.47 (s, 3H). ^13^C NMR (126 MHz, DMSO-*d*_6_): δ 193.9, 150.4, 133.8, 133.1, 125.6, 118.4, 116.5, 94.6, 26.1. LC–MS: *t*_R_ = 3.46 min, purity: 95.6%, *m*/*z* [M + H]^+^: 195.

### 2-Amino-5-(2-bromoacetyl)-3-chlorobenzonitrile (**17**)

Ketone **16** (0.1 g, 0.51 mmol) and THF (20 mL) were added to a round-bottom flask equipped with a reflux condenser. CuBr_2_ (0.23 g, 1.03 mmol) was added and stirring was continued at 70 °C overnight. The mixture was poured into ice water and extracted with EtOAc (3 × 20 mL). The combined organic layers were washed with brine, dried over anhydrous Na_2_SO_4_, and filtered. The solvent was removed *in* *vacuo* to obtain a yellow solid, which was stirred with *c*-Hex (2 × 10 mL) to obtain **17** as a yellow solid. (77 mg, 55%). ^1^H NMR (600 MHz, DMSO-*d*_6_): δ 8.19 (d, *J* = 2.0 Hz, 1H), 8.05 (d, *J* = 2.0 Hz, 1H), 7.29 (s, 2H), 4.81 (s, 2H). ^13^C NMR (151 MHz, DMSO-*d*_6_): δ 187.9, 150.9, 134.6, 133.8, 122.3, 118.5, 116.3, 94.7, 33.3. LC–MS: *t*_R_ = 3.99 min, purity: 93.5%, *m*/*z* [M + H]^+^: 273.

### 2-Amino-5-(2-(*tert*-butylamino)-1-hydroxyethyl)-3-chlorobenzonitrile (**18**)

A mixture of bromide **17** (0.1 g, 0.37 mmol) and EtOH (20 mL) was cooled in an ice bath. *tert*-BuNH_2_ (0.08 mL, 1.83 mmol) was added, maintaining the temperature at 0–10 °C. This was followed by stirring at rt overnight. the The reaction mixture was cooled in an ice bath and NaBH_4_ (14 mg, 0.37 mmol) was added slowly. Stirring was continued in an ice bath for 1 h. The reaction mixture was concentrated *in* vacuo and purified by flash column chromatography using DCM:MeOH 9:1 as eluent to yield the title compound as a yellow solid (24 mg, 25%). ^1^H NMR (600 MHz, CD_3_OD): δ 7.54 (d, *J* = 2.0 Hz, 1H), 7.39 (d, *J* = 2.0 Hz, 1H), 4.60 (t, *J* = 6.7 Hz, 1H), 2.75 (d, *J* = 6.7 Hz, 2H), 1.18 (s, 9H). ^13^C NMR (151 MHz, CD_3_OD): δ 148.0, 133.8, 133.0, 130.0, 120.4, 117.8, 97.2, 71.8, 52.9, 50.5, 28.1. LC–MS: *t*_R_ = 2.42 min, purity: 99.2%, *m*/*z* [M + H]^+^: 268. HRMS calcd for C_13_H_19_ClN_3_O, [M + H]^+^ = 268.1211; found, 268.1208.

### Molecular Superpositions

Molecular superpositions were generated using Chemical Computing Group’s Molecular Operating Environment (MOE, version 2022.02). The PDB structure of the β_2_ adrenoceptor in complex with BI-167107 (3P0G.pdb) was downloaded from the RCSB Protein Data Bank and subsequently protonated using MOE’s Protonate 3D module (using the default settings, except for the pH that was adjusted to a value of 7.4). Next, all protein and solvent atoms were deleted, and the atoms of the reference ligand BI-167107 were fixed to retain the bioactive conformation. The structure of *trans*-**12b** and, subsequently, the structure of *cis*-**12b** were superposed using the Flexible Alignment module, using the default settings except for putting more weight on the Volume similarity term (adjusted from 1 to 3) and deselecting the H-bond Acceptor term. The stereochemistry of clenbuterol, *trans*-**12b** and *cis*-**12b** at the chiral carbon atom was kept the same (i.e., *R*) as for BI-167107. The best scoring superpositions were selected (for *trans*-**12b** this resulted in the following scores: average strain energy *U* = 21.8092 kcal/mol and similarity measure *F* = −125.5665 and grand alignment score *S* = −103.7573; For *cis*-**12b** this resulted in the scores: *U* = 23.4297 and *F* = −123.0105 and *S* = −99.5809; for clenbuterol this resulted in the scores: *U* = 13.0166 and *F* = −246.6033 and *S* = −233.5868).

### Photochemistry

UV–vis spectra were recorded using a Thermo-scientific Evolution 201 PC spectrophotometer equipped with a thermostatted cell holder set at 20 °C. Fits of UV–vis spectroscopy data were generated using Prism 9.4.1. Illumination for almost all photochemical experiments was executed using a Sutter instruments Lambda LS with a 300 W full-spectrum lamp connected to a Sutter instruments Lambda 10–3 optical filter changer equipped with 360 ± 20 nm, 434 ± 9 nm and 520 ± 12 nm. The light intensity used in all illuminations is 0.93 mW/mm^2^ using the 360 ± 20 nm filter, 0.79 mW/mm^2^ for the 434 ± 9 nm filter, 0.20 mW/mm^2^ for the 520 ± 12 nm filter and 0.15 mW/mm^2^ for the 560 ± 5 nm filter as measured using a Thorlabs PM16–401 power meter. For photochemical analyses, illuminations were performed in Hellma Suprasil quartz 114QS cuvettes. For the dynamic illuminations (e.g., Figure 2D), a 4-way cuvette holder (Avantes) was used. The left outlet of the holder is connected to an Avantes AvaLight-DH-S-BAL Deuterium lamp. The right outlet is connected through a fiber to an AvaSpec UV/vis spectrometer. The lower outlet is connected to a Lambda 721 optical beam combiner system equipped with 365 ± 11 nm and 440 ± 25 nm illumination means. The light intensity used in all illuminations is 200 mW using the 365 ± 11 nm filter, and 560 mW for the 440 ± 25 nm filter. The upper outlet is covered with a black cap. The temperature of the solution in the cuvette (20 °C) is controlled by Q-blue Wireless Temperature Control software. Thermal relaxation experiments and Arrhenius extrapolations were performed according to Priimagi et al.,^37^ using a compound concentration of 25 μM in HBSS +1% DMSO and temperatures of 50, 60, and 70 °C (or 65, 70, 75 °C). Illuminations for pharmacological experiments were performed in cylindrical clear glass vials with a volume of 4.5 mL. The typical distance between light source and vial or cuvette was 2 cm.

### Materials and Construct

All cell culture reagents were purchased from Thermo Fisher Scientific (Breda, The Netherlands). Fetal bovine serum (FBS, #S00E110004) was purchased from BODINCO BV (Alkmaar, Netherlands). Zeocin (#ant-an-1) were obtained from Invivogen (Toulouse, France). Linear 25 kDa polyethylenimine (PEI, #23966-5) was obtained from Polysciences (Warrington, United States). Poly­(ethylenimine) solution (branched PEI, #P3141) and Dulbecco’s phosphate-buffered saline (PBS, #D8662-6 × 500 mL) were obtained from Sigma-Aldrich (Missiouri, United States). Bovine Serum Albumin (BSA, #A6588,0100) was purchased from PanReac AppliChem (Monza, Italy). [^3^H]­Dihydroalprenolol ([^3^H]­DHA, #NET720) was obtained from PerkinElmer (USA). Isoprenaline (I6504–100MG) was purchased from Sigma-Aldrich (The Netherlands). Black–black 96-well plates (#655086) were obtained from Greiner (Alphen aan den Rijn, Netherlands).

### Cell Culture

Human embryonic kidney (HEK) 293T cells (ATCC, CRL-1573) were cultured in Dulbecco’s modified eagles medium (DMEM, #41966-029) supplemented with 10% FBS and 1% penicillin/streptomycin (PenStrep, #15140) and cultured in a humidified incubator at 37 °C with 5% CO_2_. HEK293 cells stably expressing a cAMP FRET biosensor consisting of EPAC fused to the FRET pair mCerulean and mCitrine were kindly provided by Dr. M Zimmermann (InterAx Biotech, Switzerland),^38^ and maintained in DMEM supplemented with 10% FBS and 1% penicillin/streptomycin (PenStrep, #15140), and 0.06 mg/mL Zeocin in a humidified incubator at 37 °C with 5% CO_2_. Human β_2_AR (GenBank: NP_000015.1) in pcDNA3.1+ expression plasmid and mammalian expression plasmid pcDEF3 have been previously described.^15,39^

### Radioligand binding Assay

Two million HEK293T cells were seeded in a 10 cm^2^ tissue-culture dish with DMEM supplemented with 10% FBS and 1% penicillin/streptomycin (PenStrep, #15140) 24 h before transfection. Cells were transiently transfected with 1 μg β_2_AR plasmid and 4 μg empty pcDEF3 plasmid using 30 μg 25 kDa linear PEI. After 48 h, cells were collected in ice-cold PBS and centrifuged at 1500 g at 4 °C for 10 min. The cell pellets were thereafter resuspended in ice-cold Tris–HCl buffer (15 mM, supplemented with 0.3 mM EDTA and 2 mM MgCl_2_ at pH 7.4) and subsequently homogenized by plunging a pestle 10 times (Tamson, The Netherlands). The homogenates were twice frozen–thawed with liquid-nitrogen and membranes were collected with ultracentrifugation in 40,000 g at 4 °C. The protein content was determined by using a BCA protein assay kit (Thermo Scientific, The Netherlands).

Radioligand displacement experiments were conducted on 0.1–15 μg membranes expressing β_2_AR using 1.8–3 nM [^3^H]­DHA in combination with increasing concentrations of clenbuterol or photoswitchable ligands (*trans*-isomer without preillumination, and PSS_*cis*_ after preillumination with 360 or 520 nm for 10 min) in 100 μL binding buffer (HBSS containing 0.1% BSA) for 1 h at 25 °C. Incubations were terminated by rapid filtration over a 0.5% PEI (branched PEI)-coated 96-well GF/C filter plate followed by three rapid wash steps with ice-cold wash buffer (50 mM Tris–HCl and 500 mM NaCl, pH 7.4) using a Perkin-Elmer 96-well harvester (Perkin-Elmer, Groningen, The Netherlands). The GF-C filter plates were dried at 52 °C for 45 min and 25 μL Microscint-O scintillation liquid was applied per well. Filter-bound radioactivity was measured using a Microbeta Wallac Trilux scintillation counter (Perkin-Elmer, Groningen, The Netherlands) after a 120 min delay.

### FRET-Based EPAC cAMP Assay

HEK293 cells stably expressing EPAC cAMP biosensor were seeded (50,000 cells/well) in a black–black 96-well plate 24 h before the assay. Next, the culture medium was replaced with HBSS, and cells were incubated with 10^–5^ M to 10^–11^ M reference compounds (isoprenaline and clenbuterol) or photoswitchable ligands (*trans*-isomer without preillumination and PSS_*cis*_ after preillumination with 360 ± 20 nm for 10–20 min or 520 ± 12 nm for 50 min at 25 °C. For the antagonist curves, cells were incubated with 10^–5^ M to 10^–11^ M reference compounds (propranolol, clenbuterol) or photoswitchable ligands in the presence of 13–20 nM isoprenaline (EC_80_ value) for 15 min at 25 °C. Fluorescent intensity was measured using the CLARIOstar Plus plate reader (BMG Labtech, Germany) with 430/15 nm excitation, 480/20 and 530/20 nm emission wavelengths. The FRET ratio was calculated as acceptor (530 nm) divided by donor (480 nm) signals and subsequently normalized to the vehicle (0%) and the maximum response induced by isoprenaline (100%).

### Data Analysis

Data were analyzed by nonlinear regression analysis using GraphPad Prism 10.1.0. The EC_50_ or IC_50_ values were obtained by nonlinear regression using global fitting with a shared bottom value. *K*_i_ or *K*_b_ values of ligands were calculated using the Cheng–Prusoff equation.^40^ Each graph represented the pooled data from three independent experiments performed in duplicates.

## Supplementary Material





## Notes

Excel file with molecular formula strings (CSV)
Photochemical characterization of compounds **12a–e**, pharmacological studies of compounds **12a**, **12c–e,** and **18**, copies of NMR spectra of compounds **12a–e**, and copies of LCMS and HRMS traces for compounds **12a–e** (PDF)

## Abbreviations

ADR: Adrenergic receptor
β_2_-AR: β_2_-adrenergic receptor
[^3^H]-DHA: dihydroalprenolol
EPAC: exchange protein activated by 3′-5′-cyclic adenosine monophosphate [cAMP]
HBSS: Hank’s Balanced Salt Solution
HEK293: human embryonic kidney 293
PSS: photostationary state.
