# Supplementary material for: Photo-clenbuterol: Optical Control of β2‑Adrenergic Receptor Signaling by Photoswitchable Ligand Efficacy
Source: J Med Chem. 2025 Jun 10;68(12):12911–24. doi: 10.1021/acs.jmedchem.5c00792 (PMC12225454; doi:10.1021/acs.jmedchem.5c00792)
Supplement: Supplementary file 2 [file jm5c00792_si_002.pdf]

SUPPORTING INFORMATION

Photo-clenbuterol: optical control of β$_2$-adrenergic receptor signaling by photoswitchable ligand efficacy

Yangzhi Cao,[1] Shuang Shi,[1] Simone A. H. Does,[1] Christian M. L. Buzink,[1] Meichun Gao,[1] Iwan J. P. de Esch,[1] Henry F. Vischer,[1] Maikel Wijtmans,[1,*] Rob Leurs[1,*]

[1]Division of Medicinal Chemistry, Amsterdam Institute of Molecular and Life Sciences (AIMMS), Vrije Universiteit Amsterdam, 1081 HZ Amsterdam, The Netherlands.

Corresponding Author

Maikel Wijtmans – Email: m.wijtmans@vu.nl

Rob Leurs – Email: r.leurs@vu.nl

Table of Contents

SUPPLEMENTARY FIGURES S3

Figure S1. Photochemical characterization of **12a** (VUF26044) S3

Figure S2. Photochemical characterization of **12c** (VUF26125). S4

Figure S3. Photochemical characterization of **12d** (VUF26175). S5

Figure S4. Photochemical characterization of **12e** (VUF26202). S6

Figure S5. LC-MS studies on the photostationary states (PSS) of **12b** (VUF26034). S7

Figure S6. ^{1}H NMR studies on the photostationary states (PSS) of **12a** (VUF26044) S8

Figure S7. ^{1}H NMR studies on the photostationary states (PSS) of **12b** (VUF26034). S9

Figure S8. ^{1}H NMR studies on the photostationary states (PSS) of **12c** (VUF26125). S10

Figure S9. ^{1}H NMR studies on the photostationary states (PSS) of **12d** (VUF26175). S11

Figure S10. ^{1}H NMR studies on the photostationary states (PSS) of **12e** (VUF26202). S12

Figure S11. Thermal (dark) relaxation of *cis*-**12b** (VUF26034) S13

Figure S12. LC-MS studies on the photostationary states (PSS) of **12b** (VUF26034) using 560 nm light. S13

Figure S13. Pharmacology properties of **18** (VUF26211) for β_2-AR. S14

Figure S14. Competition binding curves of photoswitchable clenbuterol analogs for β_2-AR. S14

Figure S15. cAMP generation induced by photoswitchable clenbuterol analogs. S15

Figure S16. Inhibition of cAMP generation by photoswitchable clenbuterol analogs. S15

^{1}H-, ^{13}C-NMR spectra and LC-MS, HRMS figures of final compounds S16

SUPPLEMENTARY FIGURES

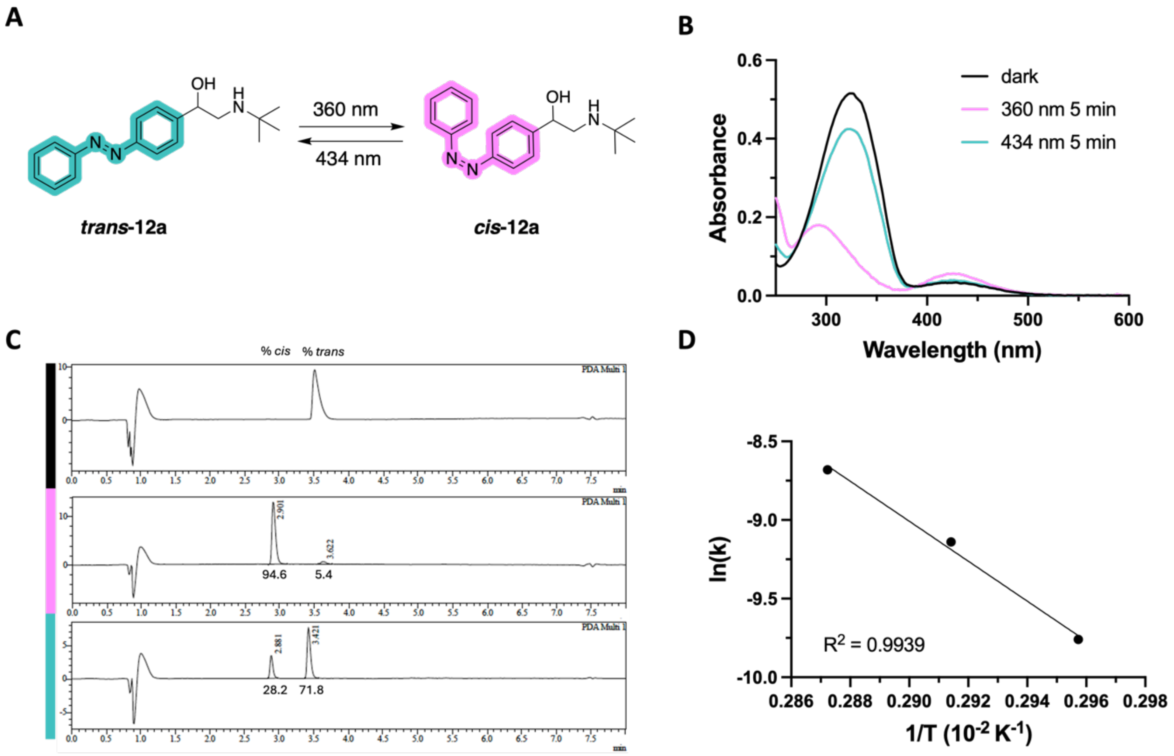

Figure S1. Photochemical characterization of **12a** (VUF26044). (A) Chemical structures of the isomers of **12a**. (B) UV-vis spectra of 25 μM of **12a** in HBSS buffer containing 1% DMSO as the *trans* isomer (black), after illumination with 360 ± 20 nm for 5 min to PSS$_{cis}$ (magenta) and after subsequent illumination with 434 ± 9 nm for 5 min to PSS$_{trans}$ (cyan). (C) *Trans*-**12a** (upper panel) and photostationary state (PSS) area percentages after illumination with 360 ± 20 nm for 10 min to reach PSS$_{cis}$ (middle panel) and after subsequent illumination with 434 ± 9 nm for 10 min to reach PSS$_{trans}$ (lower panel) at 10 mM in DMSO as determined by LC-MS analysis at the isosbestic point (390 nm). The injection peak is a result of the DMSO present. Some drifting of retention times was observed in these same-day experiments, but MS and UV analyses ensured the right assignment of peaks and therefore the drifting was deemed non-interfering. (D) Arrhenius fit for the thermal relaxation of *cis*-**12a** at three different temperatures (65 °C, 70 °C, 75 °C), as measured in 25 μM HBSS buffer containing 1% DMSO.

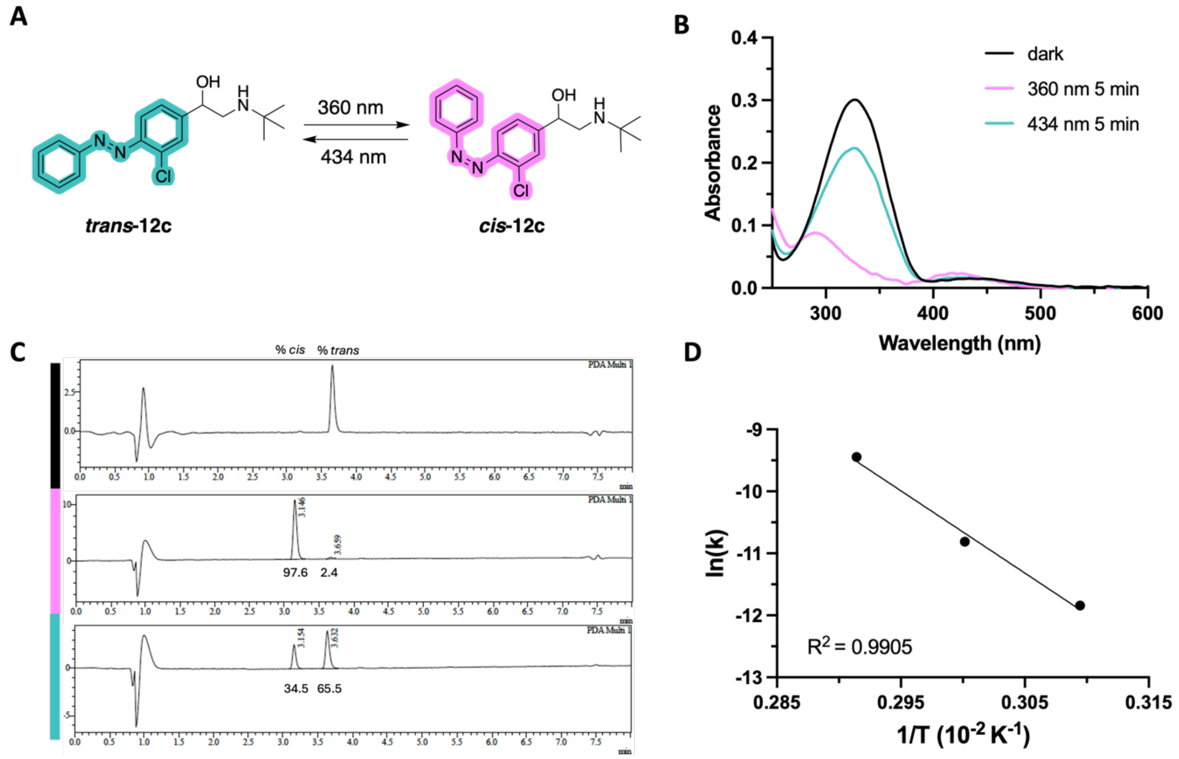

Figure S2. Photochemical characterization of **12c** (VUF26125). (A) Chemical structures of the isomers of **12c**. (B) UV-vis spectra of 25 μM of **12c** in HBSS buffer containing 1% DMSO as the *trans* isomer (black), after illumination with 360 ± 20 nm for 5 min to PSS_{cis} (magenta) and after subsequent illumination with 434 ± 9 nm for 5 min to PSS_{trans} (cyan). (C) *Trans*-**12c** (upper panel) and Photostationary state (PSS) area percentages after illumination with 360 ± 20 nm for 20 min to reach PSS_{cis} (middle panel) and after subsequent illumination with 434 ± 9 nm for 10 min to reach PSS_{trans} (lower panel) at 10 mM in DMSO as determined by LC-MS analysis at the isosbestic point (393 nm). The injection peak is a result of the DMSO present. Some drifting of retention times was observed in these same-day experiments, but MS and UV analysis ensured the right assignment of peaks and therefore the drifting was deemed non-interfering. (D) Arrhenius fit for the thermal relaxation of *cis*-**12c** at three different temperatures (50 °C, 60 °C, 70 °C), as measured in 25 μM HBSS buffer containing 1% DMSO.

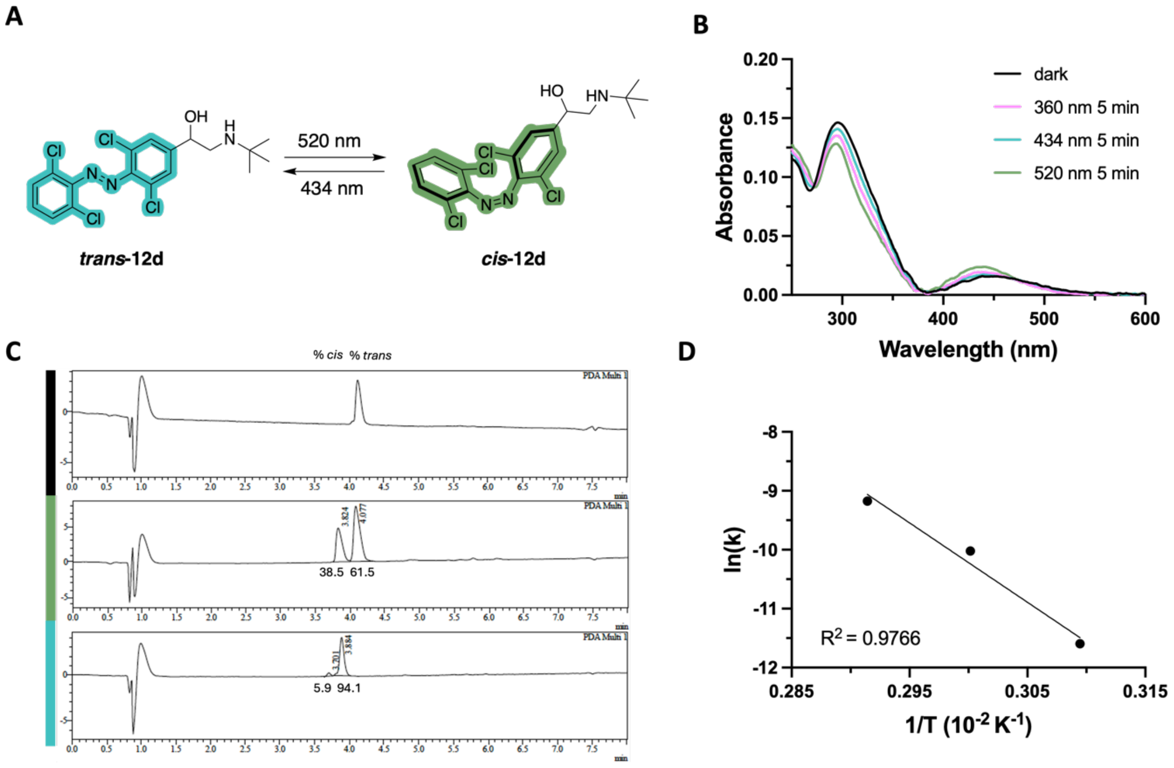

Figure S3. Photochemical characterization of **12d** (VUF26175). (A) Chemical structures of the isomers of **12d**. (B) UV-vis spectra of 25 μM of **12d** in HBSS buffer containing 10% DMSO (to aid in solubilizing **12d**) as the *trans* isomer (black), after illumination with 360 ± 20 nm (magenta) for 5 min or 520 ± 12 nm (green) for 5 min to PSS_{cis} and after subsequent illumination with 434 ± 9 nm (cyan) for 5 min to PSS_{trans}. (C) *Trans*-**12d** (upper panel) and photostationary state (PSS) area percentages after illumination with 520 ± 12 nm for 50 min to reach PSS_{cis} (middle panel) and after subsequent illumination with 434 ± 9 nm for 10 min to reach PSS_{trans} (lower panel) at 10 mM in DMSO as determined by LC-MS analysis at the isosbestic point (370 nm). The injection peak is a result of the DMSO present. Some drifting of retention times was observed in these same-day experiments, but MS and UV analysis ensured the right assignment of peaks and therefore the drifting was deemed non-interfering. (D) Arrhenius fit for the thermal relaxation of *cis*-**12d** at three different temperatures (50 °C, 60 °C, 70 °C), as measured in 25 μM HBSS buffer containing 1% DMSO.

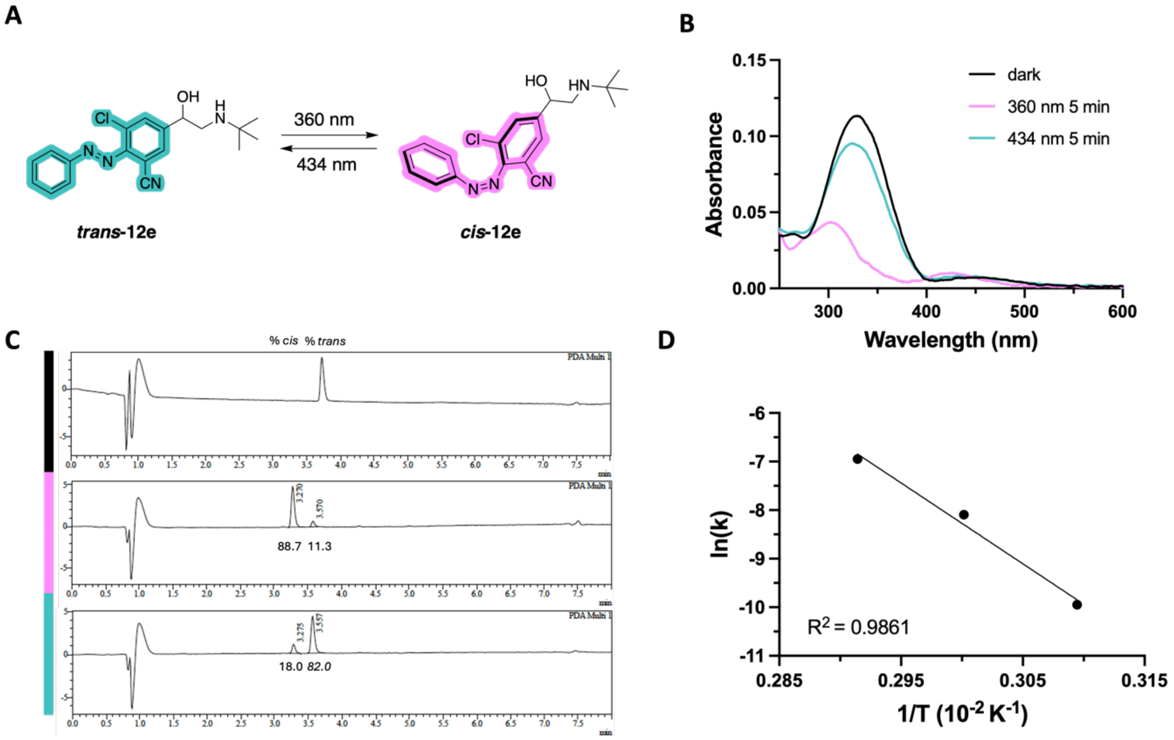

Figure S4. Photochemical characterization of **12e** (VUF26202). (A) Chemical structures of the isomers of **12e**. (B) UV-vis spectra of 25 μM of **12e** in HBSS buffer containing 1% DMSO as the *trans* isomer (black), after illumination with 360 ± 20 nm for 5 min to PSS$_{cis}$ (magenta) and after subsequent illumination with 434 ± 9 nm for 5 min to PSS$_{trans}$ (cyan). (C) *Trans*-**12e** (upper panel) and photostationary state (PSS) area percentages after illumination with 360 ± 20 nm for 10 min to reach PSS$_{cis}$ (middle panel) and after subsequent illumination with 434 ± 9 nm for 5 min to reach PSS$_{trans}$ (lower panel) at 10 mM in DMSO as determined by LC-MS analysis at the isosbestic point (396 nm). The injection peak is a result of the DMSO present. Some drifting of retention times was observed in these same-day experiments, but MS and UV analysis ensured the right assignment of peaks and therefore the drifting was deemed non-interfering. (D) Arrhenius fit for the thermal relaxation of *cis*-**12e** at three different temperatures (50 °C, 60 °C, 70 °C), as measured in 25 μM HBSS buffer containing 1% DMSO.

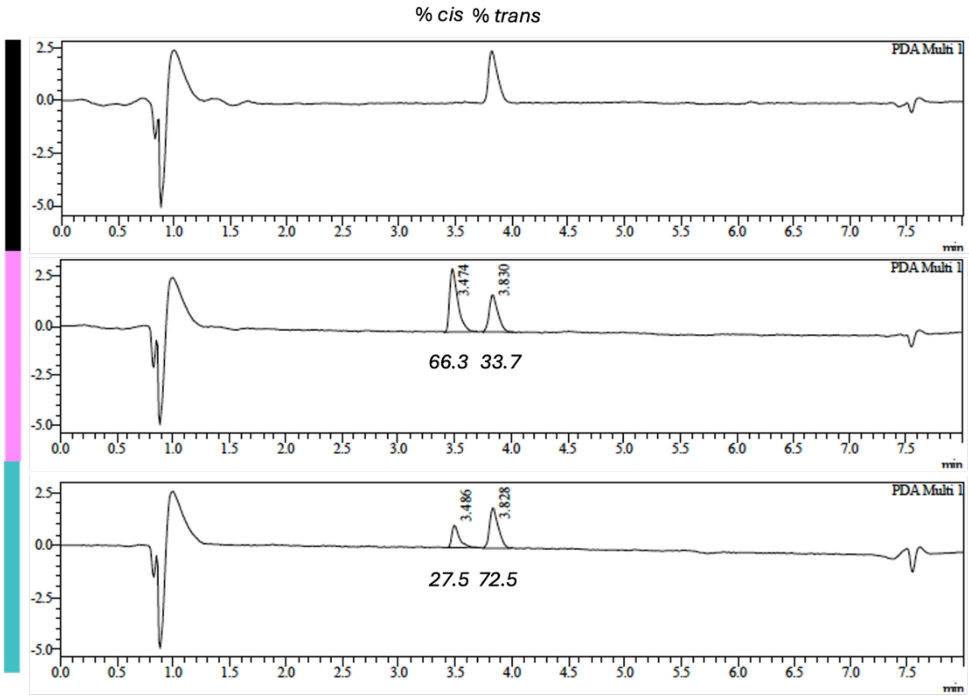

Figure S5. LC-MS studies on the photostationary states (PSS) of **12b** (VUF26034). *Trans*-**12b** (upper panel) and photostationary state (PSS) area percentages after illumination with 360 ± 20 nm for 20 min to reach PSS_{cis} (middle panel) and after subsequent illumination with 434 ± 9 nm for 20 min to reach PSS_{trans} (lower panel) at 10 mM in DMSO as determined by LC-MS analysis at the isosbestic point (376 nm). The injection peak is a result of the DMSO present.

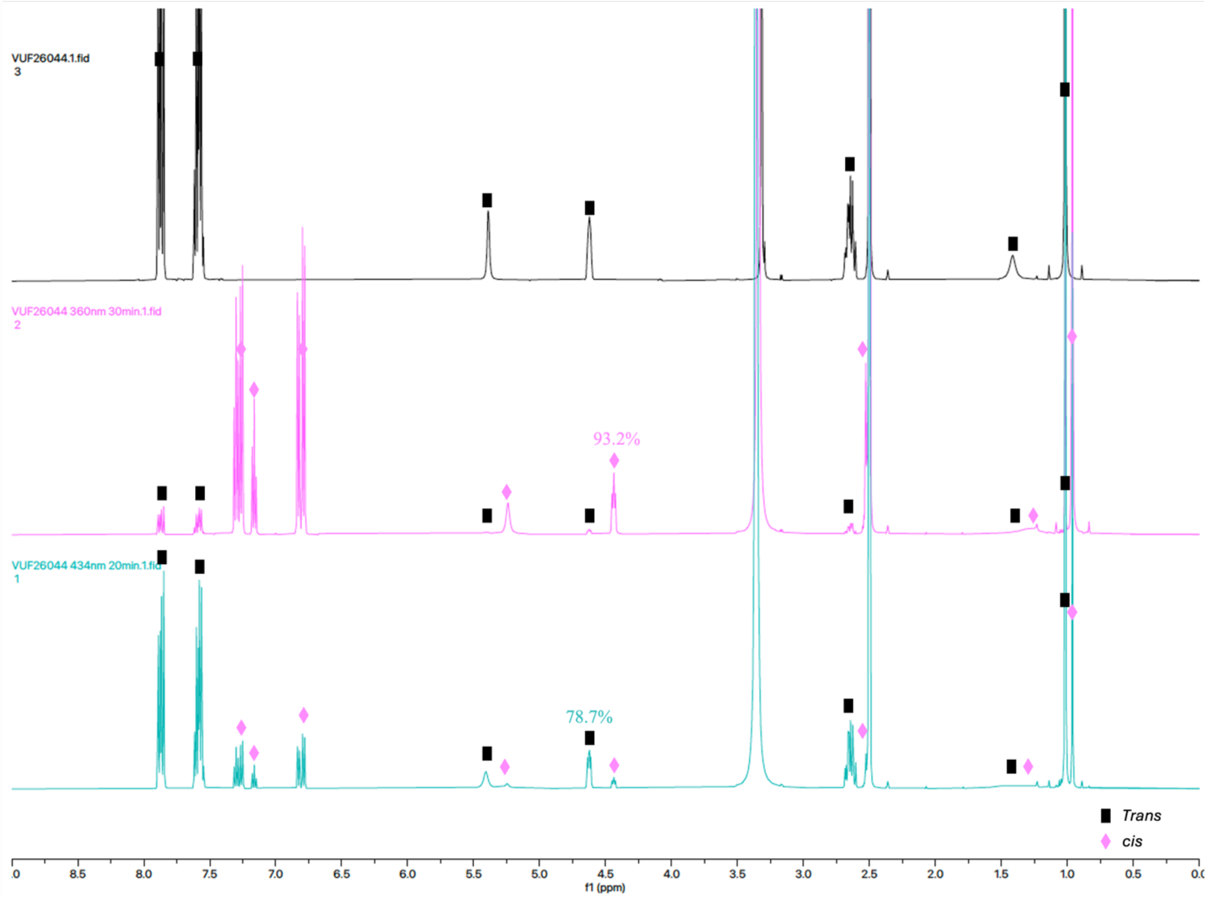

Figure S6. ^{1}H NMR studies on the photostationary states (PSS) of **12a** (VUF26044). A sample containing 10 mM **12a** in DMSO-d_6 was analyzed in dark conditions (black) and after illumination with 360 ± 20 nm for 1800 s to reach PSS$_{cis}$ (magenta) and subsequently with 434 ± 9 nm for 1200 s to reach PSS$_{trans}$ (cyan). Black squares indicate the NMR signals corresponding to the *trans* isomer and the magenta diamonds represent the NMR signals corresponding to the *cis* isomer.

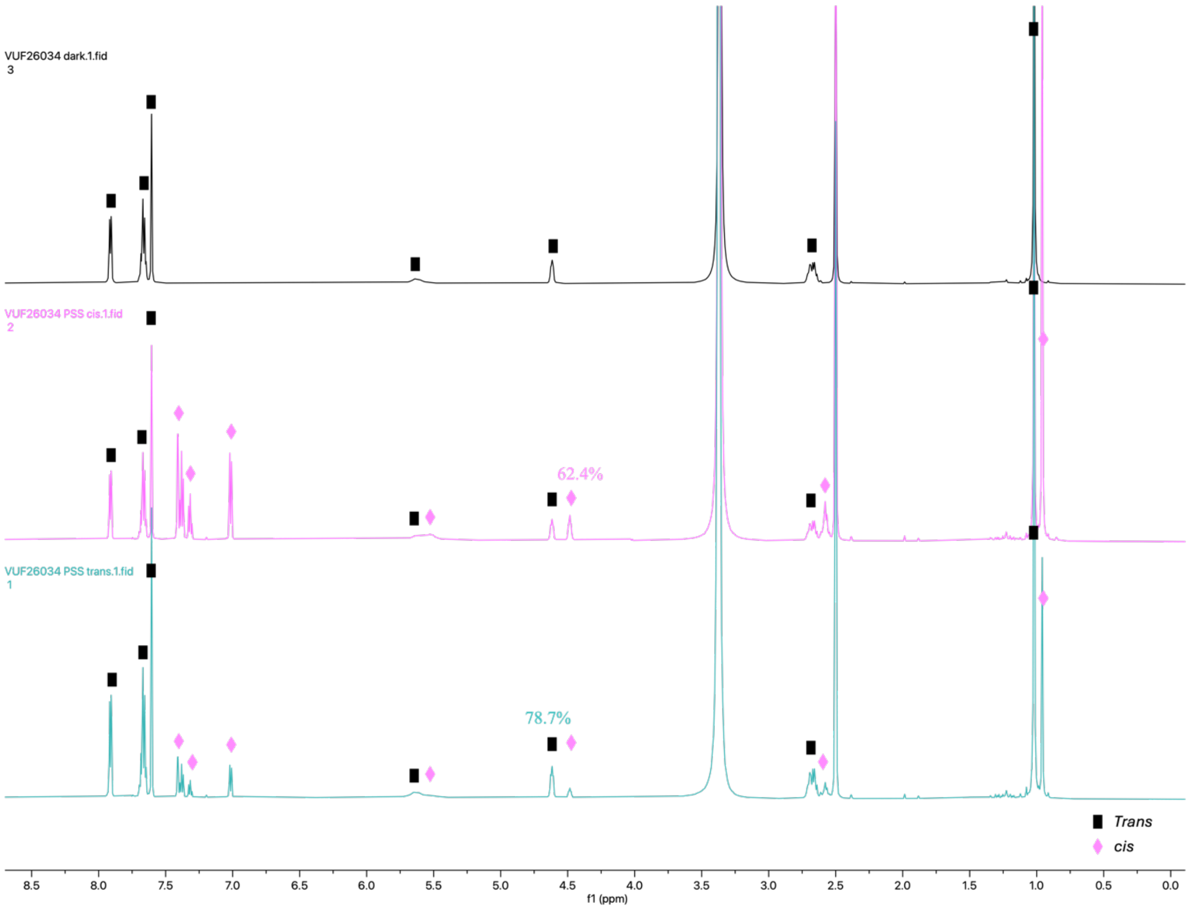

Figure S7. ^{1}H NMR studies on the photostationary states (PSS) of **12b** (VUF26034). A sample containing 10 mM **12b** in DMSO-d_6 was analyzed in dark conditions (black) and after illumination with 360 ± 20 nm for 1200 s to reach PSS_{cis} (magenta) and subsequently with 434 ± 9 nm for 1200 s to reach PSS_{trans} (cyan). Black squares indicate the NMR signals corresponding to the *trans* isomer and the magenta diamonds represent the NMR signals corresponding to the *cis* isomer.

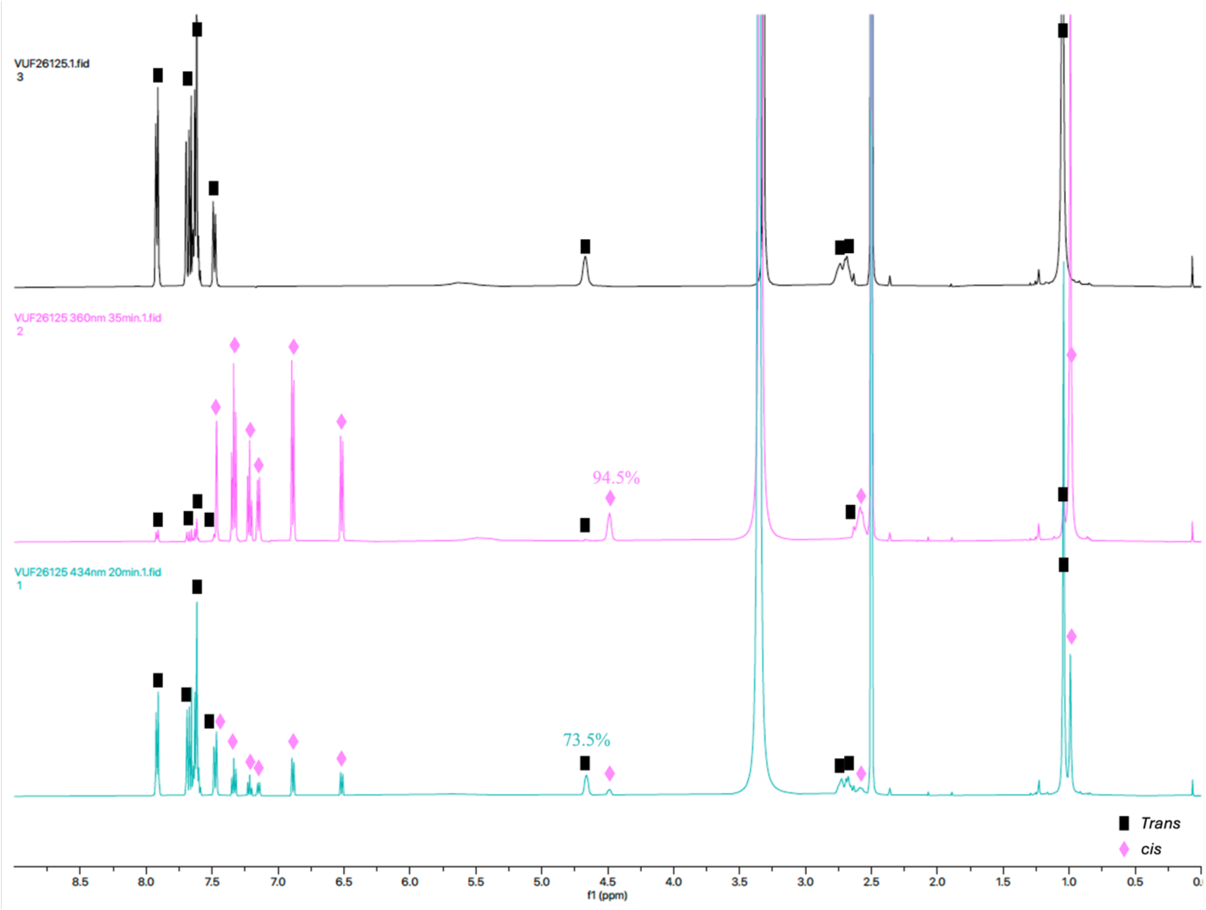

Figure S8. ^{1}H NMR studies on the photostationary states (PSS) of **12c** (VUF26125). A sample containing 10 mM **12c** in DMSO-d_6 was analyzed in dark conditions (black) and after illumination with 360 ± 20 nm for 2100 s to reach PSS$_{cis}$ (magenta) and subsequently with 434 ± 9 nm for 1200 s to reach PSS$_{trans}$ (cyan). Black squares indicate the NMR signals corresponding to the *trans* isomer and the magenta diamonds represent the NMR signals corresponding to the *cis* isomer.

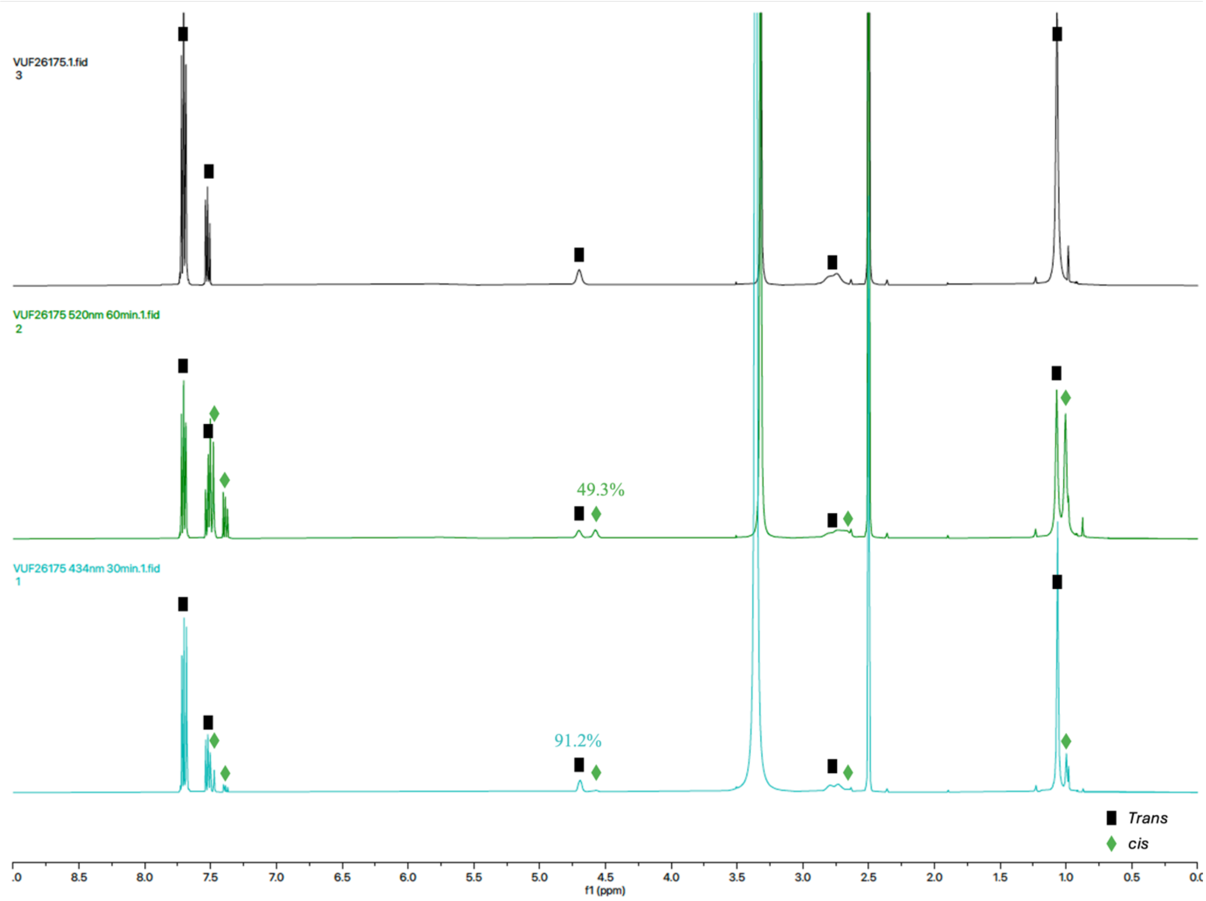

Figure S9. ^{1}H NMR studies on the photostationary states (PSS) of **12d** (VUF26175). A sample containing 10 mM **12d** in DMSO-d_6 was analyzed in dark conditions (black) and after illumination with 520 ± 12 nm for 3600 s to reach PSS_{cis} (magenta) and subsequently with 434 ± 9 nm for 1800 s to reach PSS_{trans} (cyan). Black squares indicate the NMR signals corresponding to the *trans* isomer and the magenta diamonds represent the NMR signals corresponding to the *cis* isomer.

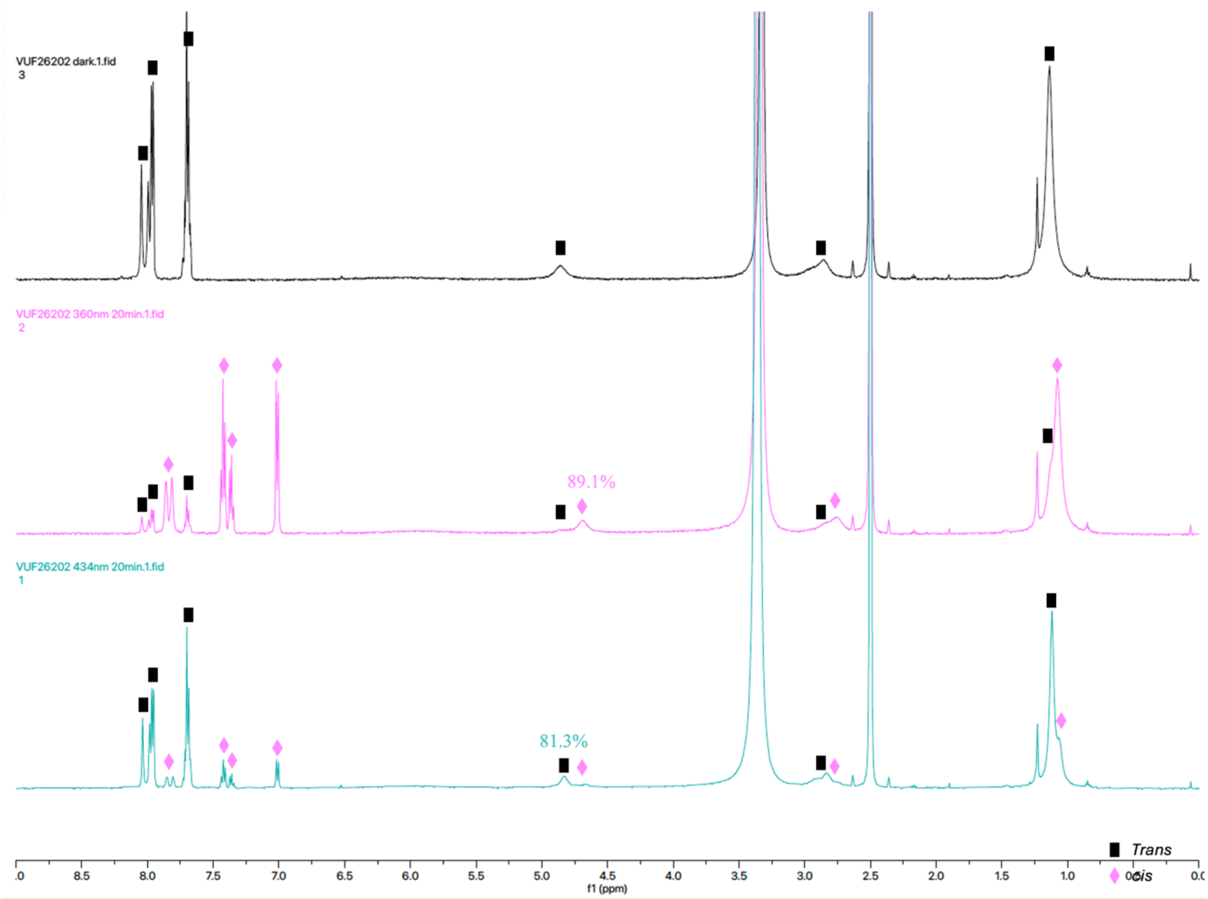

Figure S10. ^{1}H NMR studies on the photostationary states (PSS) of **12e** (VUF26202). A sample containing 10 mM **12e** in DMSO-d_6 was analyzed in dark conditions (black) and after illumination with 360 ± 20 nm for 1200 s to reach PSS_{cis} (magenta) and subsequently with 434 ± 9 nm for 1200 s to reach PSS_{trans} (cyan). Black squares indicate the NMR signals corresponding to the *trans* isomer and the magenta diamonds represent the NMR signals corresponding to the *cis* isomer.

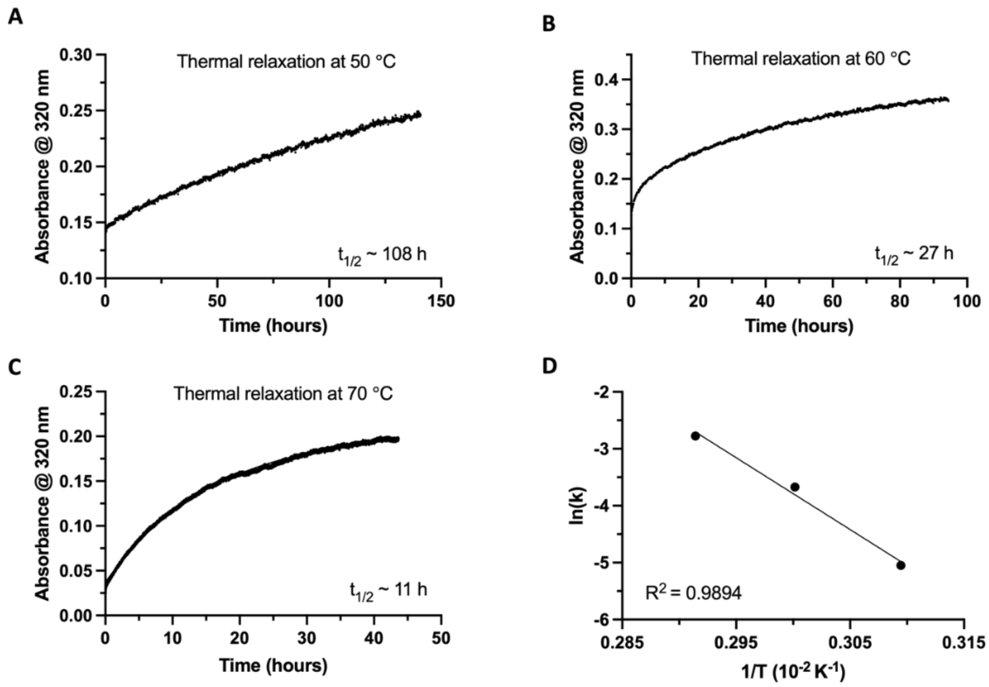

Figure S11. Thermal (dark) relaxation of *cis*-**12b** (VUF26034). (A, B, C) 25 μM of **12b** in HBSS buffer containing 1% DMSO was illuminated for 20 min at 360 ± 20 nm to PSS_{cis} prior to thermal relaxation in the dark at 50 °C, 60 °C, and 70 °C respectively, as monitored by absorbance at 320 nm at regular intervals. (D) Arrhenius fit for the thermal relaxation of *cis*-**12b** at three different temperatures (50 °C, 60 °C, 70 °C), as measured in 25 μM HBSS buffer containing 1% DMSO.

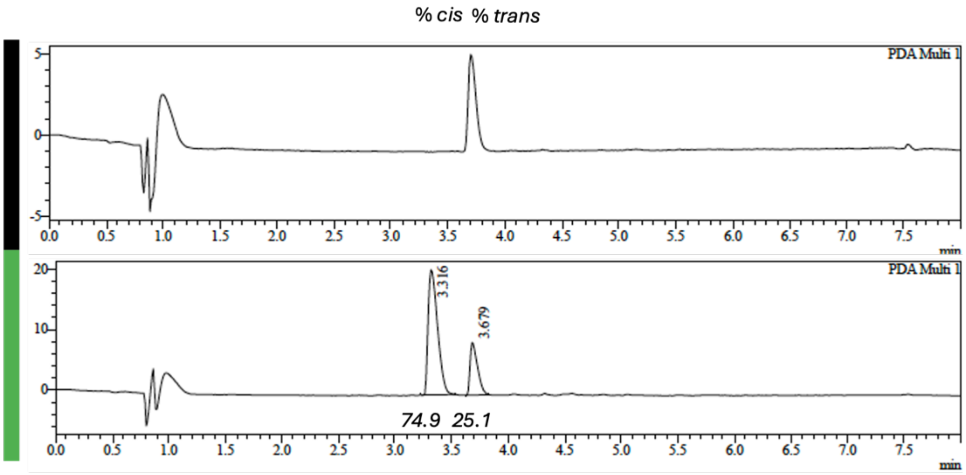

Figure S12. LC-MS studies on the photostationary states (PSS) of **12b** (VUF26034) using 560 nm light. *Trans*-**12b** (upper panel) and photostationary state (PSS) area percentages after illumination with 560 ± 5 nm for 260 min to reach PSS_{cis} (lower panel) at 10 mM in DMSO as determined by LC-MS analysis at the isosbestic point (376 nm). The injection peak is a result of the DMSO present.

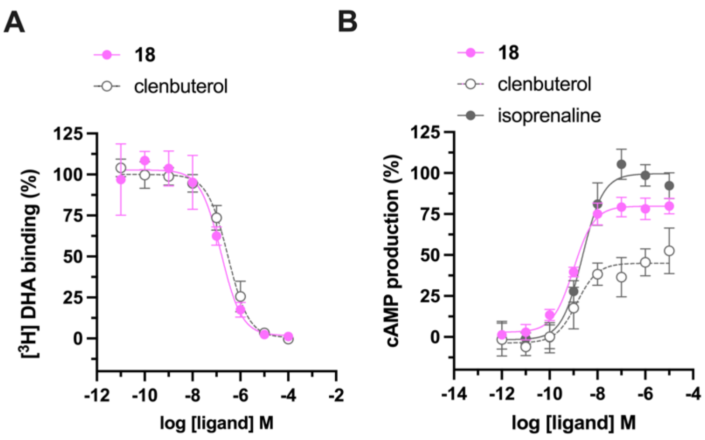

Figure S13. Pharmacology properties of **18** (VUF26211) for β2-AR. (A) Competition binding of 1.8-3 nM [^{3}H]DHA and **18**. Clenbuterol is used as the reference compound. (B) cAMP generation was detected using a FRET-based EPAC cAMP sensor post the stimulation of agonists for 15 mins. Isoprenaline and clenbuterol are shown as the reference full and partial agonist, respectively. Data are shown as mean ± SD of three independent experiments in duplicate.

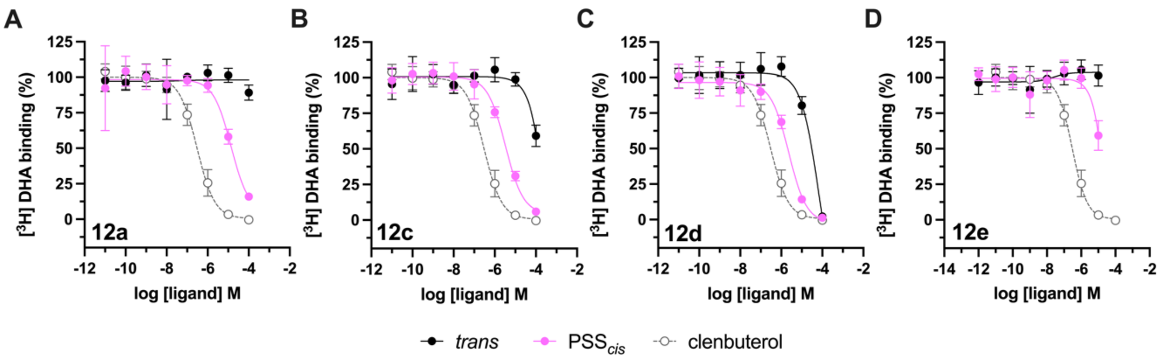

Figure S14. Competition binding curves of photoswitchable clenbuterol analogs for β2-AR. (A-D) Competition binding of 1.8-3 nM [^{3}H]DHA and increasing concentrations unlabeled (photoswitchable) ligands to HEK293T membranes expressing human β$_2$-AR. Data are shown as mean ± SD of three independent experiments in duplicate.

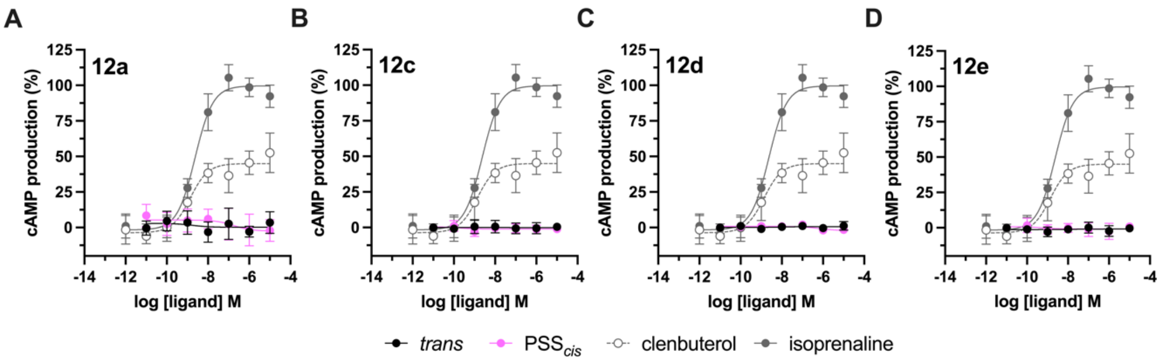

Figure S15. cAMP generation induced by photoswitchable clenbuterol analogs. (A-D) Endogenous β_2-AR-mediated cAMP production in HEK293 cells expressing the FRET-based EPAC cAMP sensor in response to ligand stimulation. Clenbuterol and isoprenaline are shown as the β_2-AR reference partial and full agonists respectively. Data are shown as mean ± SD of three independent experiments in duplicate.

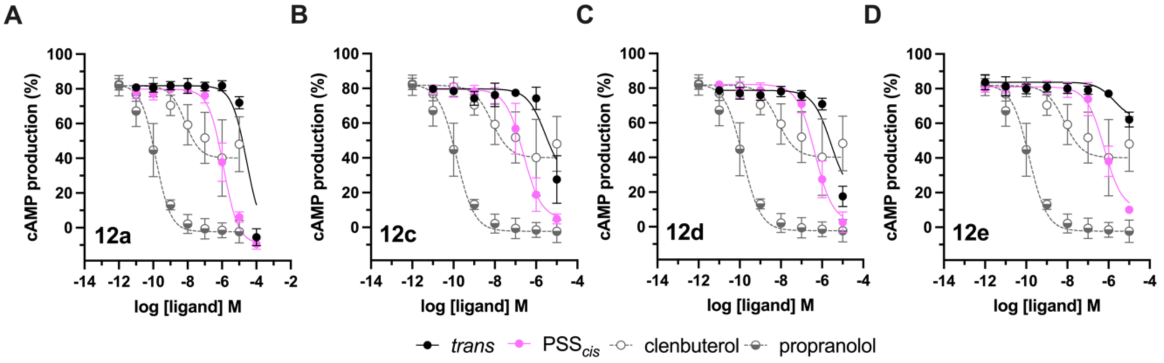

Figure S16. Inhibition of cAMP generation by photoswitchable clenbuterol analogs. (A-D) Endogenous β_2-AR-mediated cAMP production in HEK293 cells expressing the FRET-based EPAC in response to 20 nM isoprenaline (EC_{80} concentration) is antagonized by indicated ligands. Clenbuterol and propranolol are shown as the β_2-AR reference partial agonist and antagonist respectively. Data are shown as mean ± SD of three independent experiments in duplicate.

^{1}H-, ^{13}C-NMR spectra and LC-MS, HRMS figures of final compounds

^{1}H-NMR spectrum of **12a (VUF26044).**

^{1}H NMR (500 MHz, DMSO-d_6) δ 7.90 – 7.87 (m, 2H), 7.87 – 7.84 (m, 2H), 7.62 – 7.54 (m, 5H), 5.39 (br s, 1H), 4.62 (dd, J = 8.1, 4.4 Hz, 1H), 2.67 (dd, J = 11.3, 4.4 Hz, 1H), 2.63 (dd, J = 11.3, 8.1 Hz, 1H), 1.43 (br s, 1H), 1.02 (s, 9H).

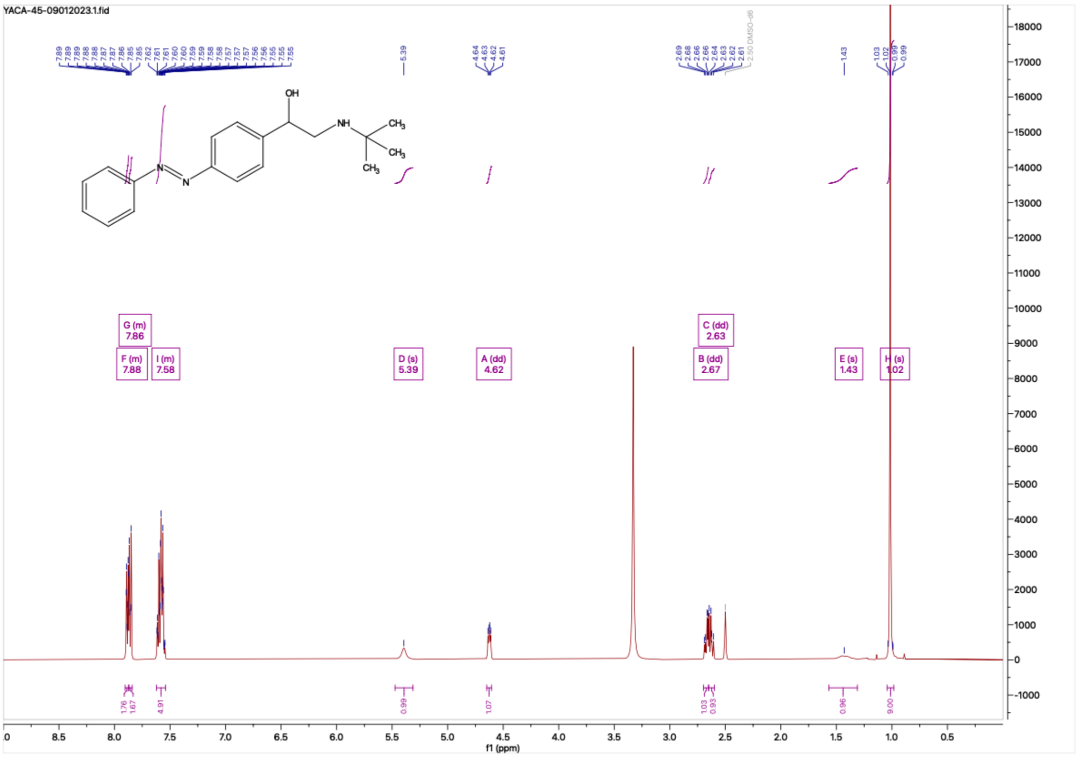

^{13}C-NMR spectrum of **12a (VUF26044)**

^{13}C NMR (126 MHz, DMSO-d_6) δ 152.0, 150.9, 148.6, 131.3, 129.4, 126.9, 122.5, 122.3, 72.2, 50.5, 49.6, 28.9.

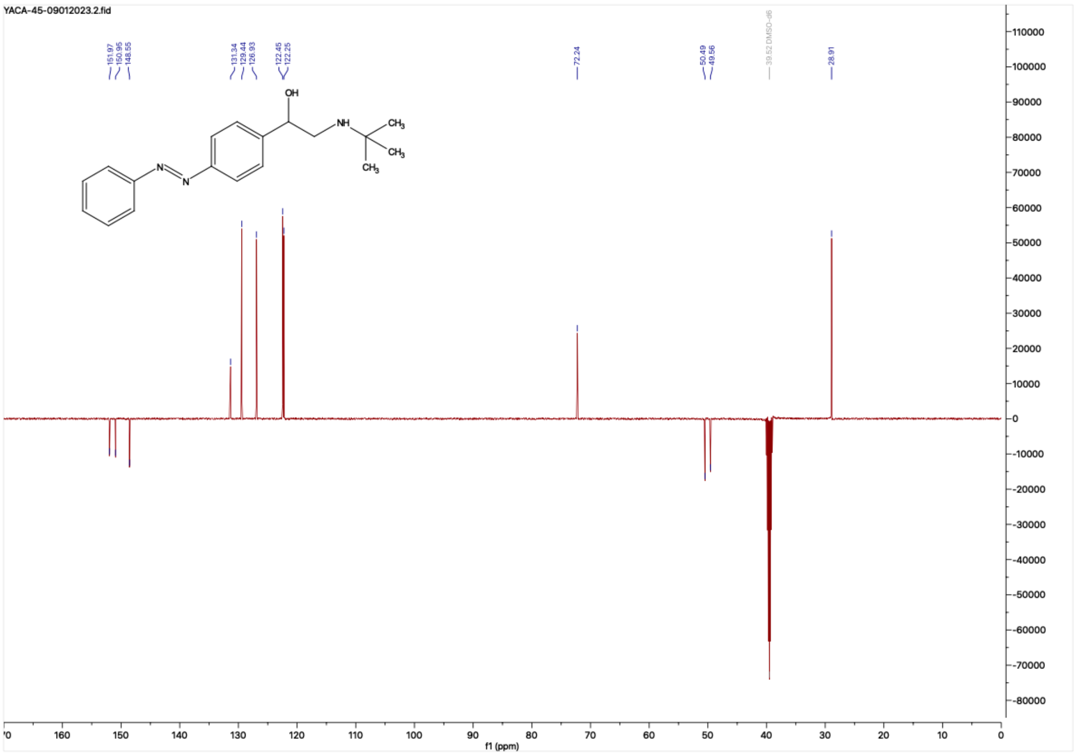

LCMS data of **12a (VUF26044)**

LC-MS: λ_{max}: 322 nm, t_R= 3.39 min, purity: 99.8% (254 nm), M/z $[M+H]^+$: 298.

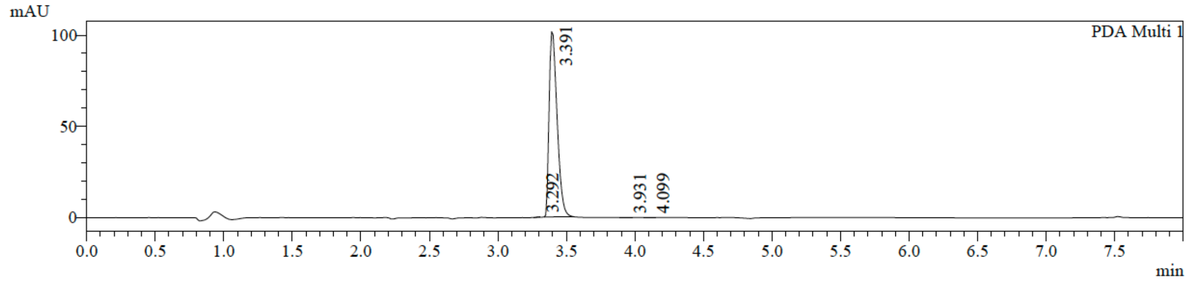

PeakTable

PDA Ch1 254nm 4nm

Peak#	Ret. Time	Area	Height	Area %
1	3.292	264	120	0.064
2	3.391	413282	101487	99.816
3	3.931	273	93	0.066
4	4.099	226	81	0.055
Total		414045	101782	100.000

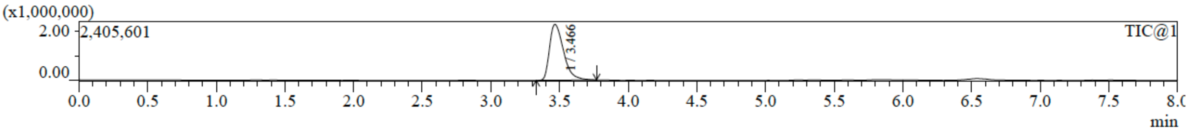

MS Spectrum Graph

#:1 Ret.Time:Averaged 3.460-3.480(Scan#:347-349)
BG Mode:Calc 3.330<->3.770(334<->378)
Mass Peaks:8 Base Peak:298.20(1631772) Polarity:Pos Segment1 - Event1

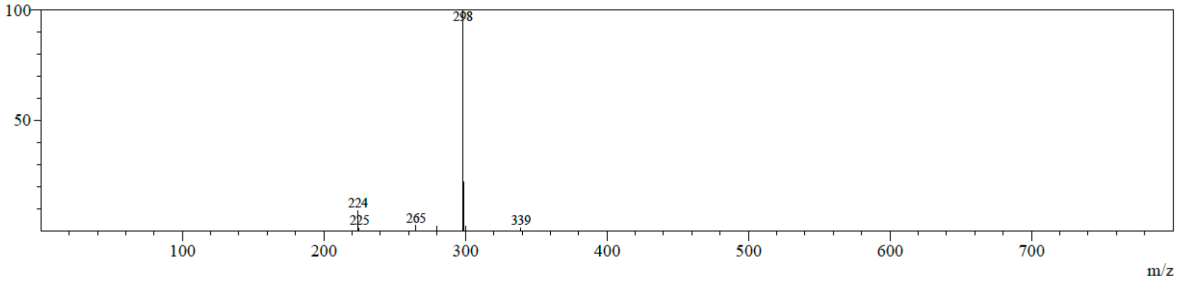

Retention Time : 3.391
Compound Name :
Spectrum Operation : None

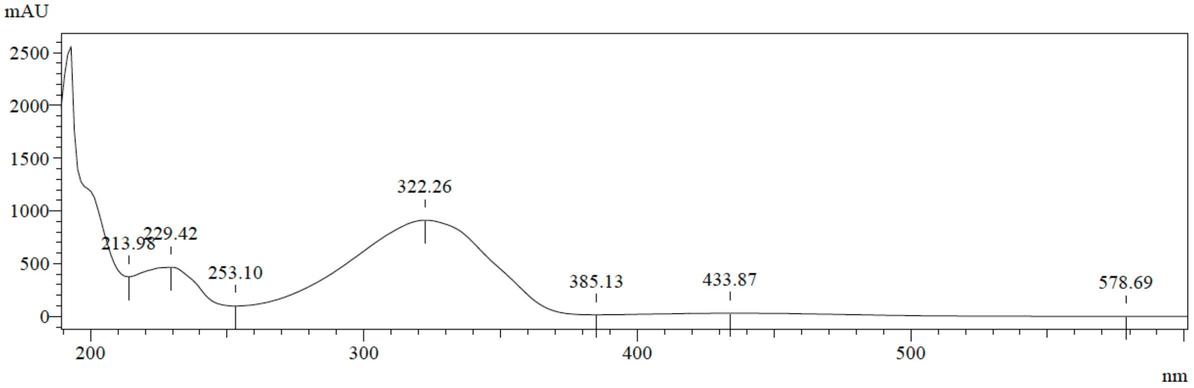

HRMS data of **12a (VUF26044)**

HRMS calcd. for $C_{18}H_{24}N_3O$ $[M+H]^+$ = 298.1914, found 298.1898.

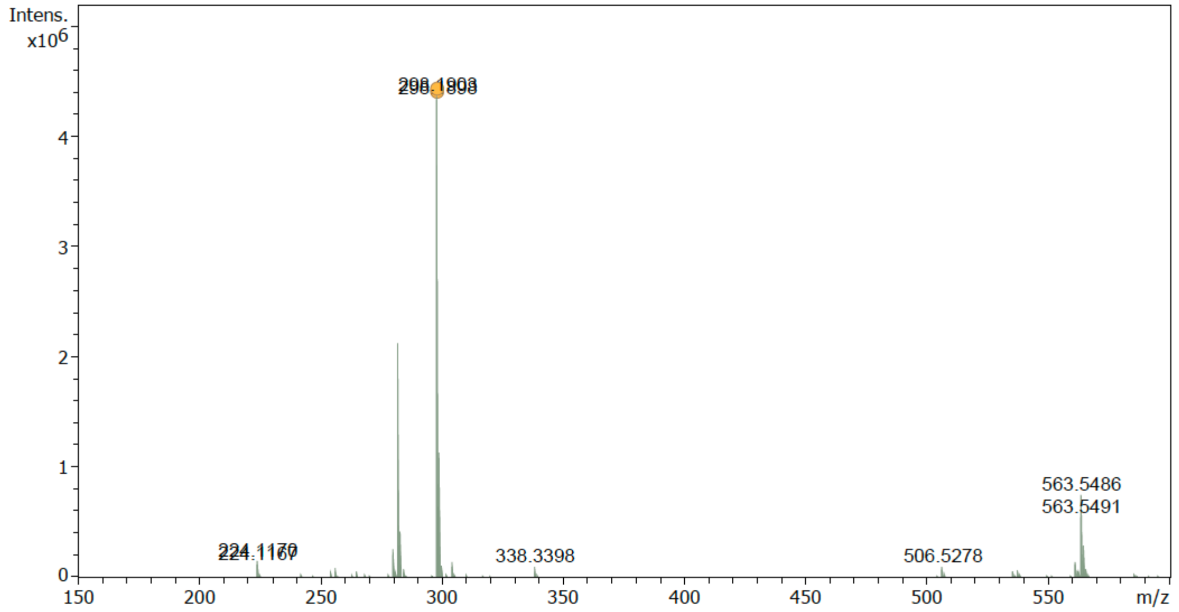

Meas. m/z	#	Ion Formula	m/z	err [ppm]	mSigma	# mSigma	Score	rdb	e⁻ Conf	N-Rule
298.1898	1	C18H24N3O	298.1914	5.3	7.9	1	100.00	9.0	even	ok
	2	C13H24N5O3	298.1874	-8.2	18.9	2	41.54	5.0	even	ok
	3	C12H28NO7	298.1860	-12.7	32.4	3	8.04	0.0	even	ok

^{1}H-NMR spectrum of **12b (VUF26034)**

^{1}H NMR (600 MHz, DMSO-d_6) δ 7.93 – 7.90 (m, 2H), 7.70 – 7.66 (m, 2H), 7.66 – 7.64 (m, 1H), 7.61 (s, 2H), 5.58 (br s, 1H), 4.61 (dd, *J* = 7.4, 4.7 Hz, 1H), 2.70 (dd, *J* = 11.4, 4.7 Hz, 1H), 2.66 (dd, *J* = 11.4, 7.4 Hz, 1H), 1.02 (s, 9H).

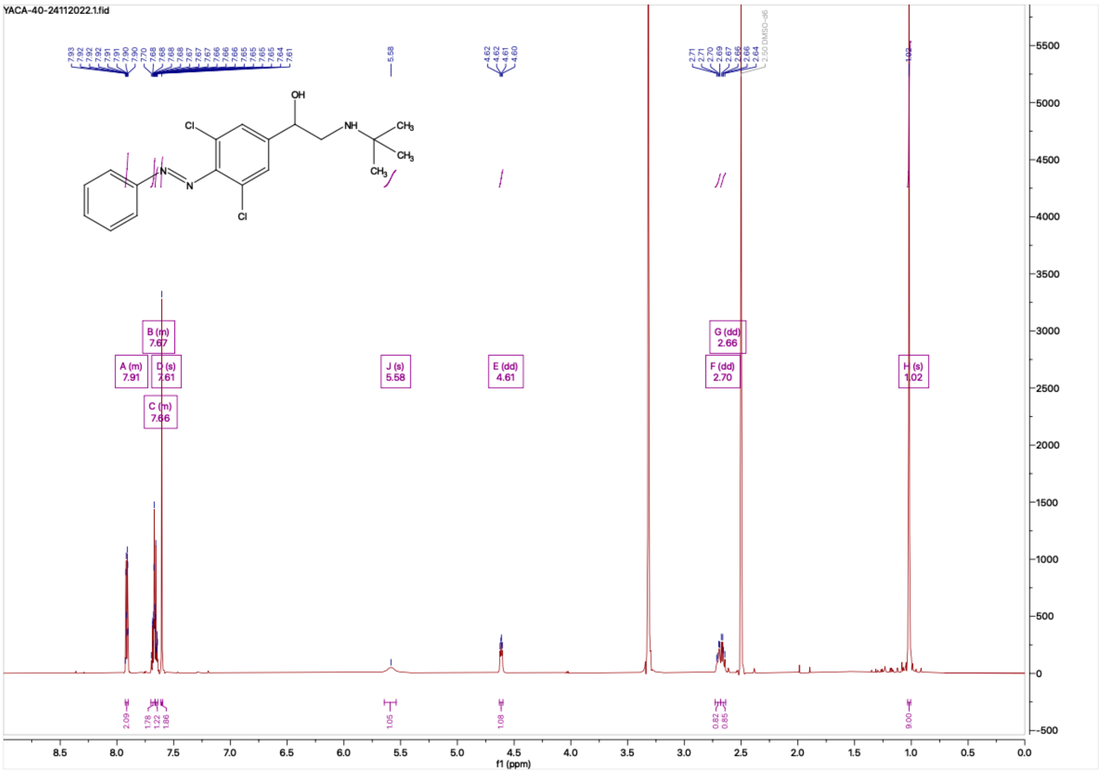

The peak at 5.58 ppm disappears upon addition of D_2O (see spectrum below).

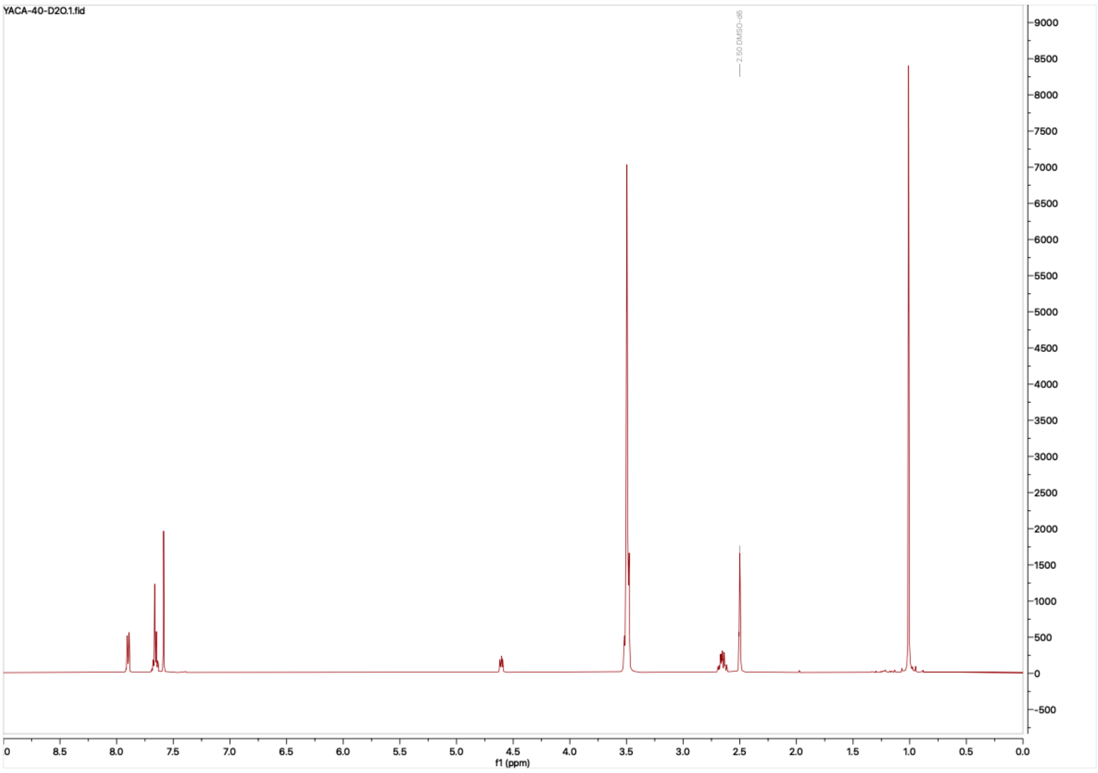

^{13}C-NMR spectrum of **12b (VUF26034)**

^{13}C NMR (126 MHz, CD_3OD) δ 153.8, 148.7, 146.9, 133.7, 130.5, 127.8, 127.6, 123.9, 72.6, 51.7, 51.0, 28.7.

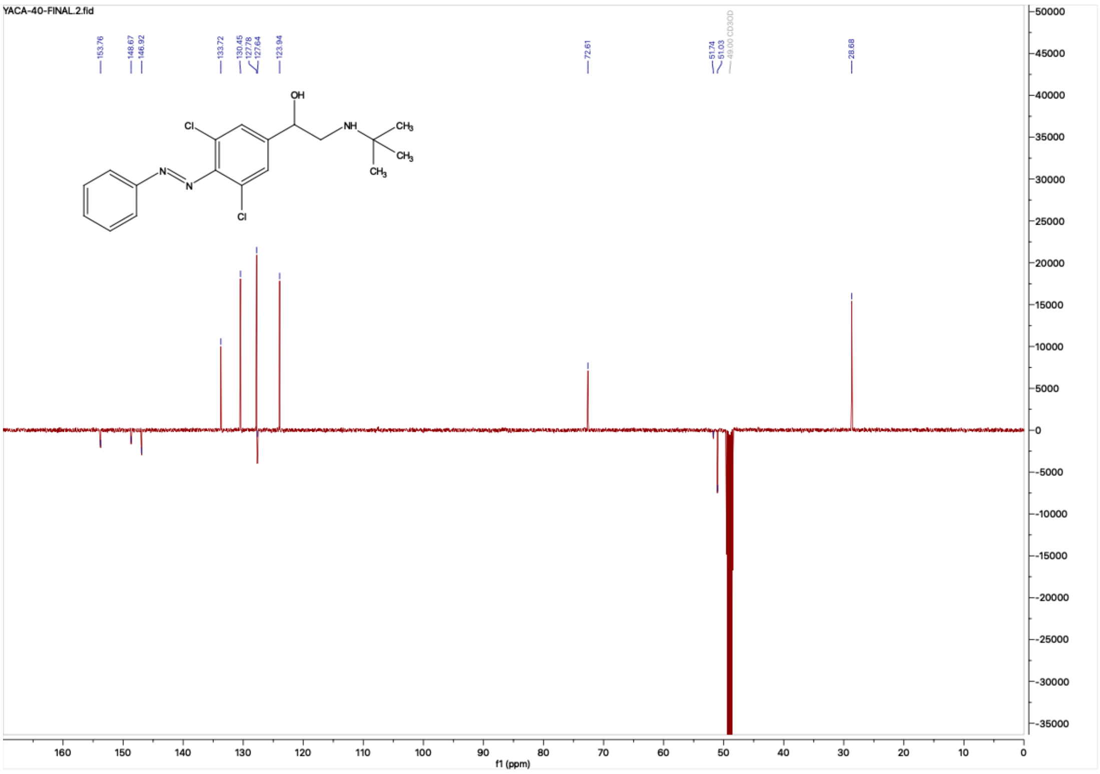

LCMS data of **12b (VUF26034)**

LC-MS: λ_{max}: 302 nm, t_R= 3.70 min, purity: 98.9%, M/z $[M+H]^+$: 366.

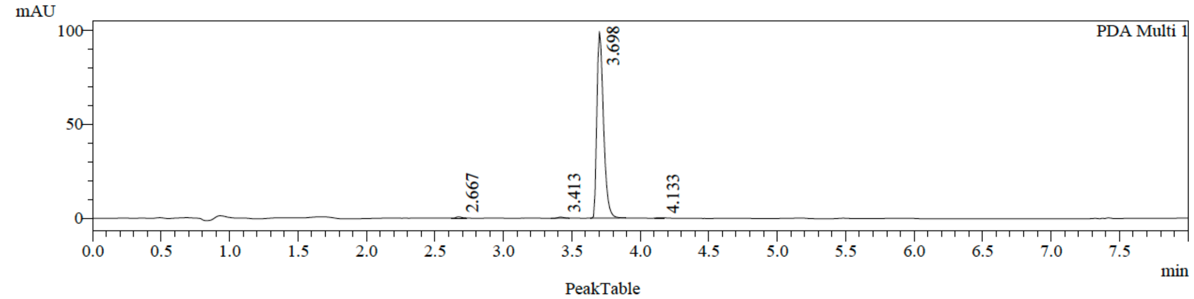

PeakTable

PDA Ch1 254nm 4nm

Peak#	Ret. Time	Area	Height	Area %
1	2.667	1779	644	0.528
2	3.413	1563	557	0.464
3	3.698	332870	99218	98.881
4	4.133	426	199	0.126
Total		336637	100619	100.000

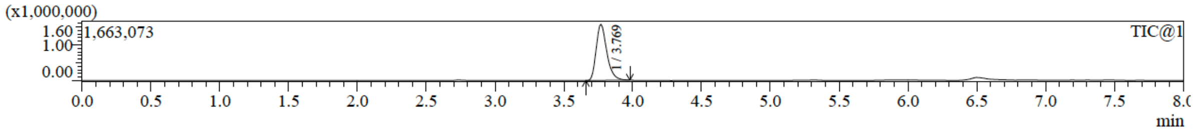

MS Spectrum Graph

#:1 Ret.Time:Averaged 3.760-3.780(Scan#:377-379)
BG Mode:Calc 3.660<->3.980(367<->399)
Mass Peaks:18 Base Peak:366.10(644786) Polarity:Pos Segment1 - Event1

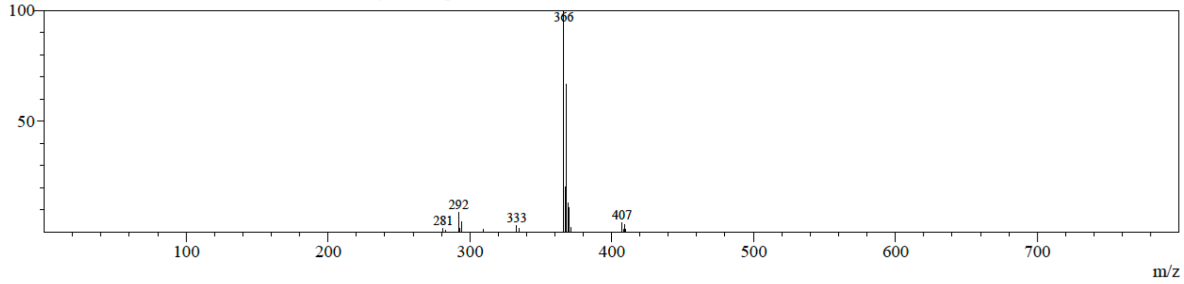

Retention Time : 3.698
Compound Name :
Spectrum Operation : None

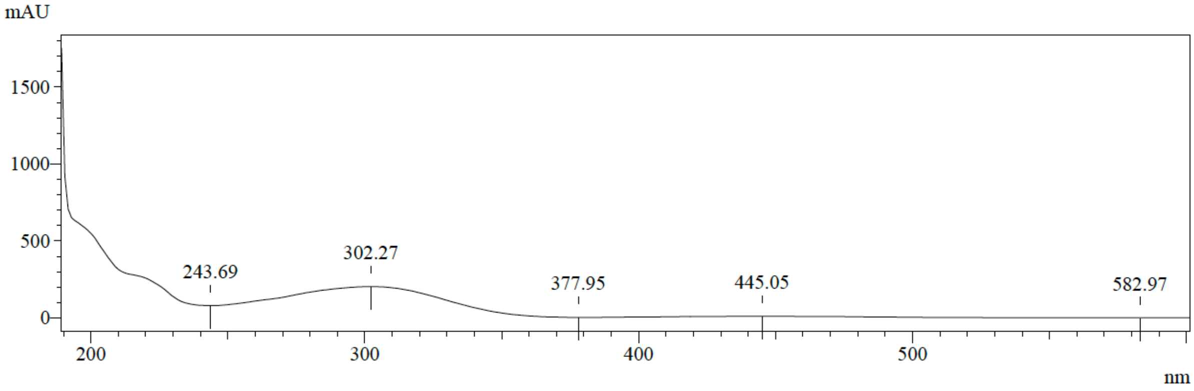

HRMS data of **12b (VUF26034)**

HRMS calcd. for $C_{18}H_{22}Cl_2N_3O$ [M+H]$^+$ = 366.1134, found 366.1132.

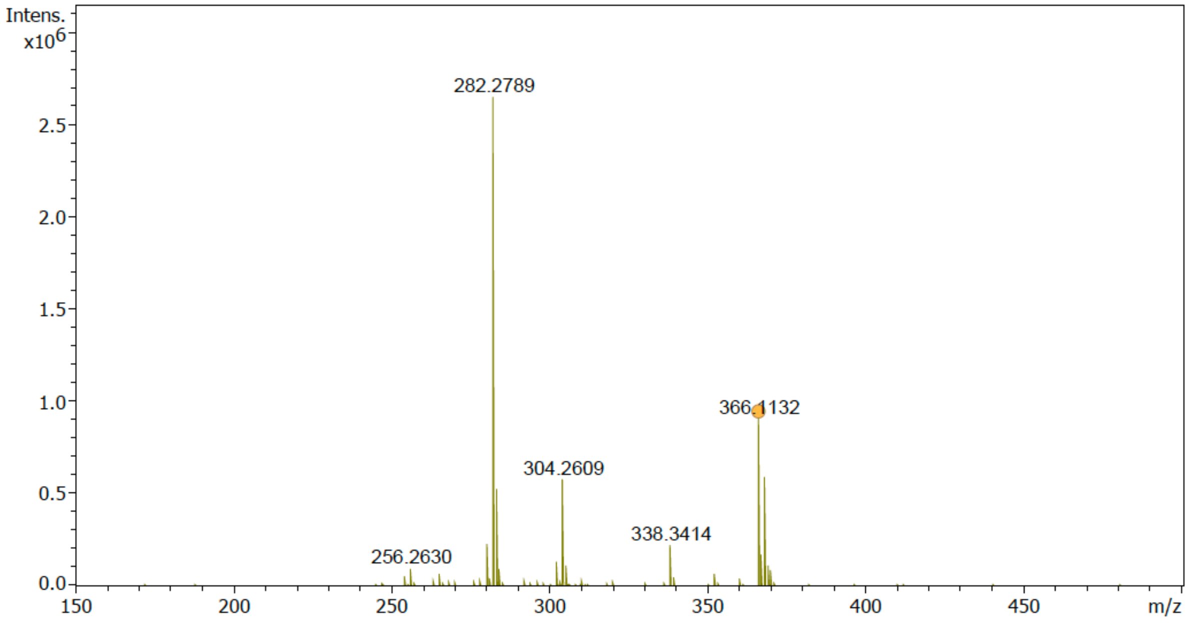

Meas. m/z	#	Ion Formula	m/z	err [ppm]	mSigma	# mSigma	Score	rdb	e⁻ Conf	N-Rule
366.1132	1	C18H22Cl2N3O	366.1134	0.6	11.6	1	100.00	15.0	even	ok

^{1}H-NMR spectrum of **12c (VUF26125)**

^{1}H NMR (500 MHz, CD_3OD) δ 7.98 – 7.93 (m, 2H), 7.72 (d, J = 8.3 Hz, 1H), 7.67 (d, J = 1.7 Hz, 1H), 7.59 – 7.52 (m, 3H), 7.43 (dd, J = 8.3, 1.7 Hz, 1H), 4.79 (dd, J = 8.6, 4.3 Hz, 1H), 2.82 (dd, J = 11.9, 4.3 Hz, 1H), 2.78 (dd, J = 11.9, 8.6 Hz, 1H), 1.18 (s, 9H).

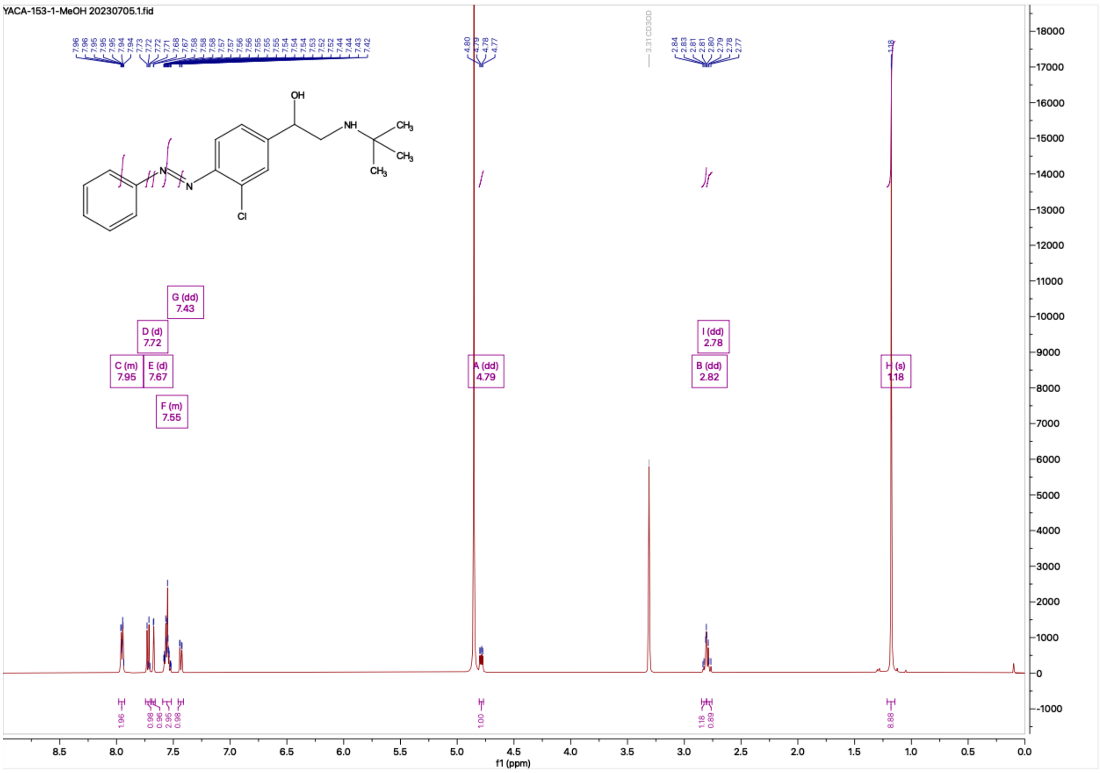

^{13}C-NMR spectrum of **12c (VUF26125)**

^{13}C NMR (151 MHz, CD_3OD) δ 154.2, 149.6, 149.1, 136.6, 132.9, 130.4, 129.2, 126.3, 124.2, 118.5, 72.8, 52.3, 50.9, 28.4.

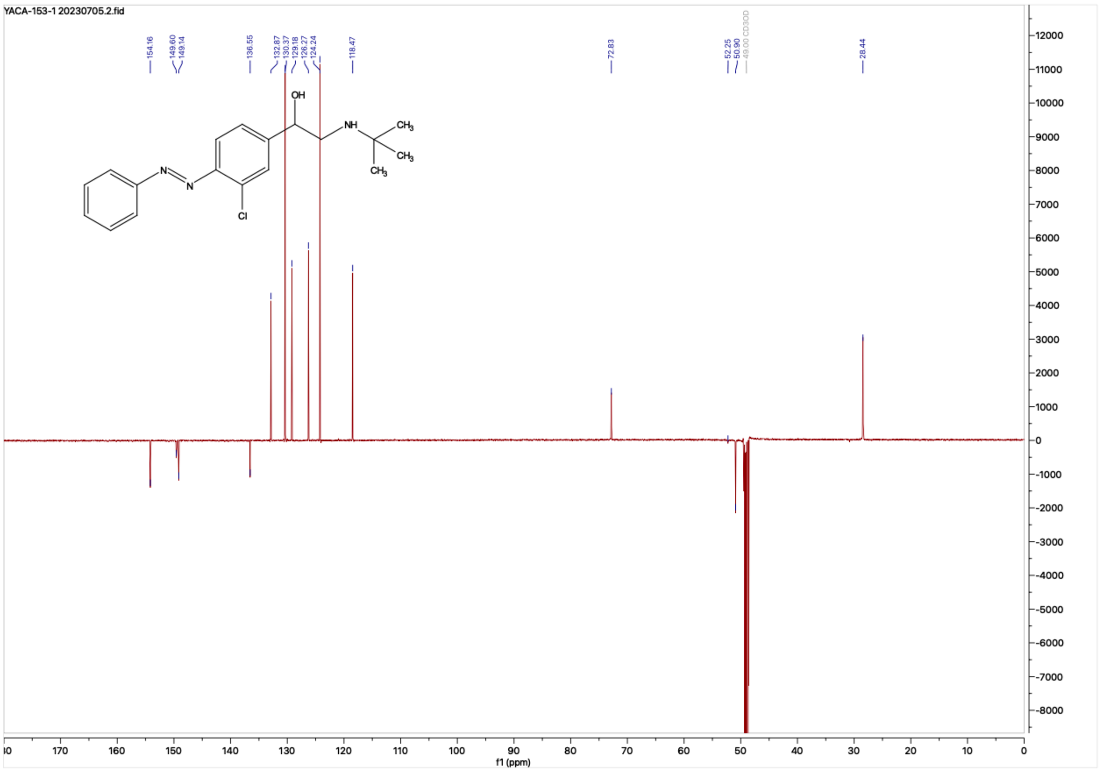

LCMS data of **12c (VUF26125)**

LC-MS: λ_{max}: 327 nm, t_R= 3.65 min, purity: 98.5%, M/z $[M+H]^+$: 332.

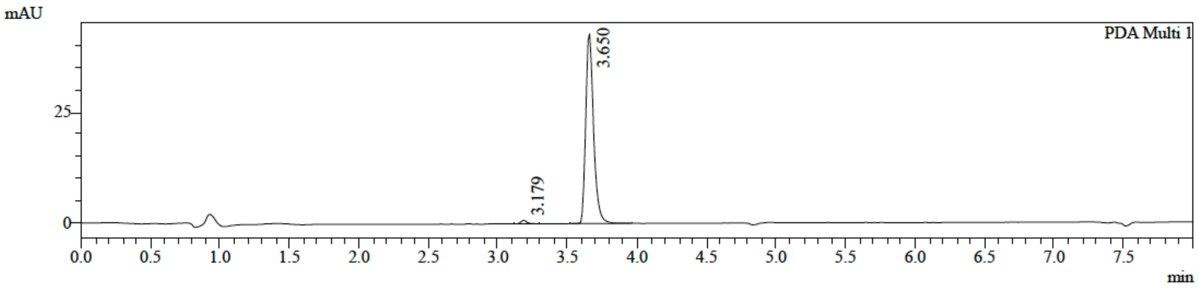

1 PDA Multi 1 / 254nm 4nm

PeakTable

PDA Ch1 254nm 4nm

Peak#	Ret. Time	Area	Height	Name	Area %
1	3.179	2569	771		1.507
2	3.650	167892	42599		98.493
Total		170461	43370		100.000

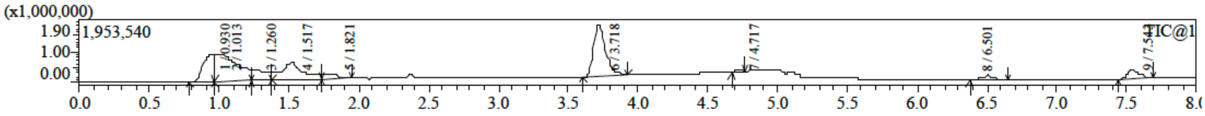

MS Spectrum Graph

#:1 Ret.Time:Averaged 3.710-3.730(Scan#:372-374)
BG Mode:Calc 3.610<->3.930(362<->394)
Mass Peaks:11 Base Peak:332.15(943354) Polarity:Pos Segment1 - Event1

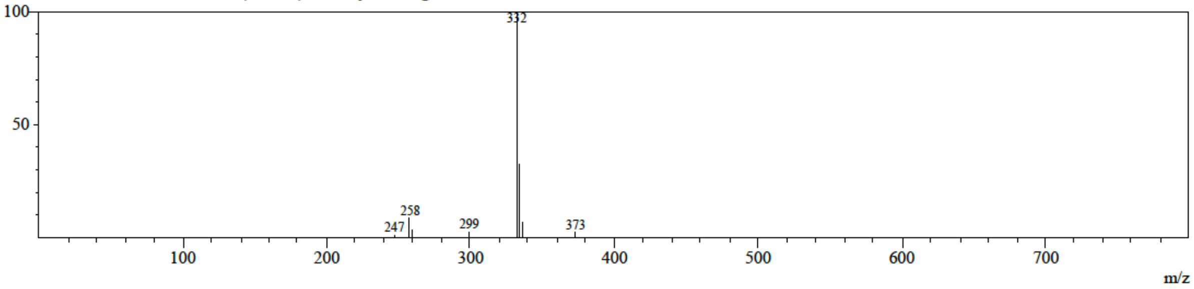

Retention Time : 3.650
Compound Name :
Spectrum Operation : None

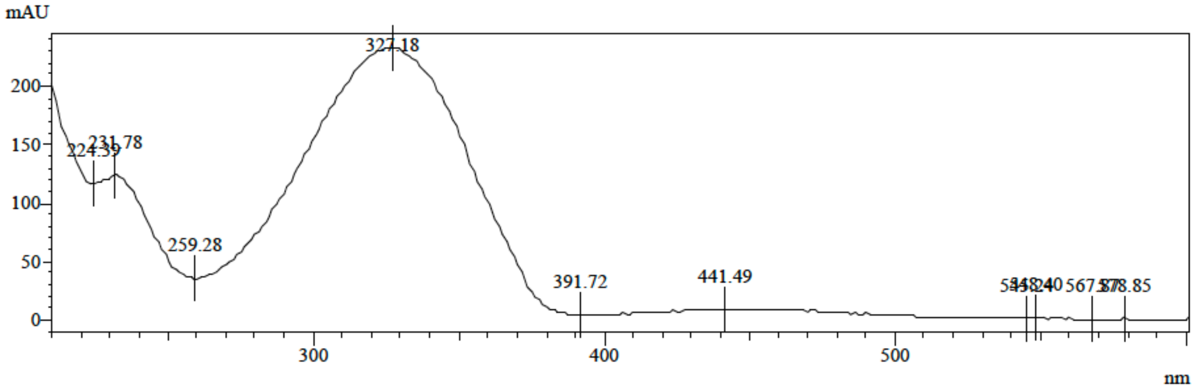

HRMS data of **12c (VUF26125)**

HRMS calcd. for $C_{18}H_{23}ClN_3O$ $[M+H]^+$ = 332.1524, found 332.1520.

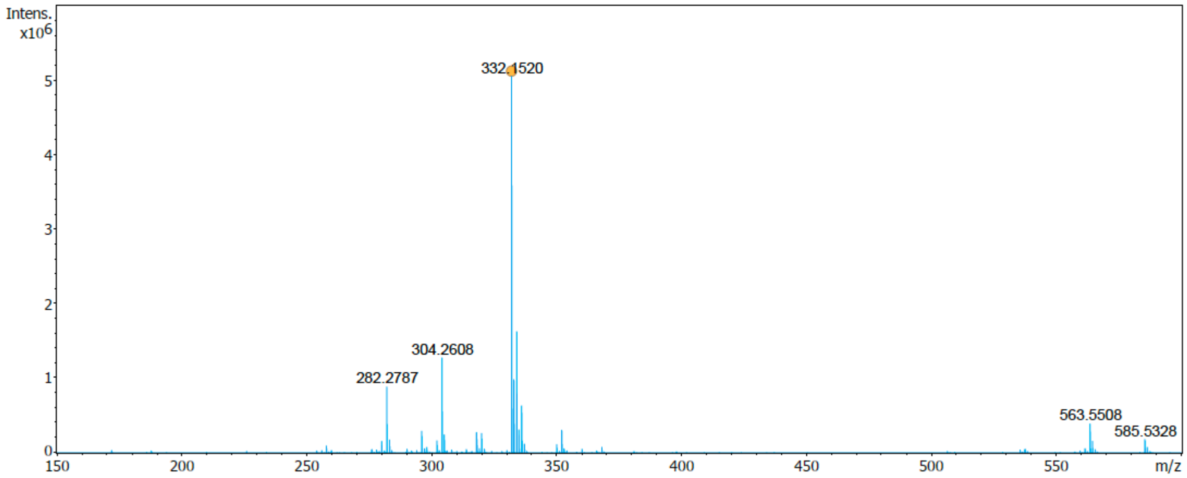

Meas. m/z	#	Ion Formula	m/z	err [ppm]	mSigma	#mSigma	Score	rdb	e⁻ Conf	N-Rule
332.1520	1	C18H23ClN3O	332.1524	1.3	12.8	1	100.00	12.0	even	ok

^{1}H-NMR spectrum of **12d (VUF26175)**

^{1}H NMR (600 MHz, CD_3OD) δ 7.63 (s, 2H), 7.58 (d, *J* = 8.1 Hz, 2H), 7.40 (t, *J* = 8.1 Hz, 1H), 4.83 – 4.79 (m, 1H), 2.96 – 2.87 (m, 1H), 2.87 – 2.78 (m, 1H), 1.22 (s, 9H).

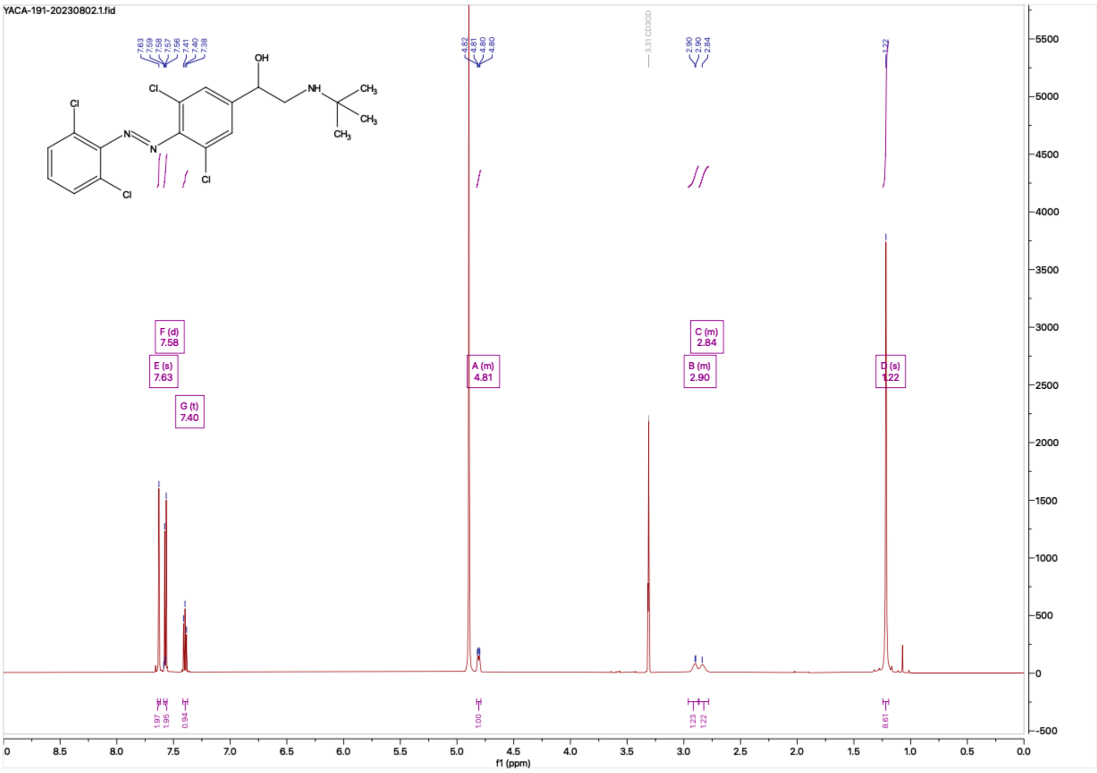

^{13}C-NMR spectrum of **12d (VUF26175)**

^{13}C NMR (151 MHz, CD_3OD) δ 148.8, 148.0, 147.6, 131.3, 130.7, 128.4, 128.2, 128.1, 72.0, 52.9, 50.6, 28.2.

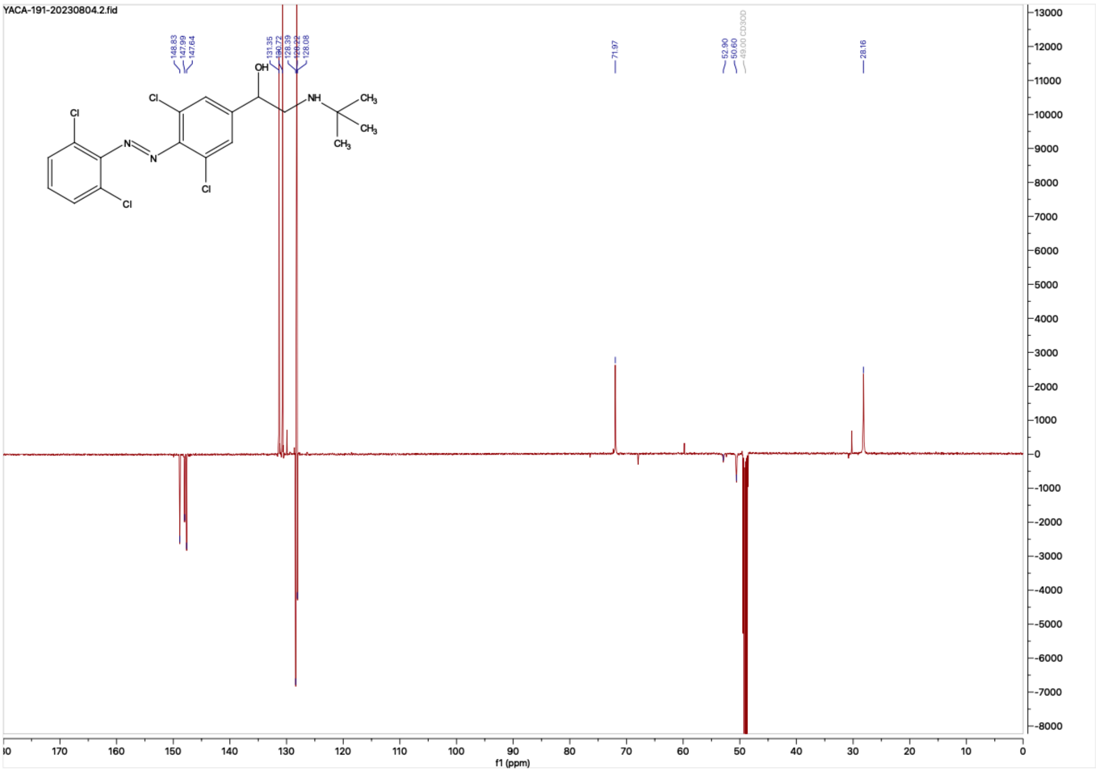

LCMS data of **12d (VUF26175)**

LC-MS: λ_{max}: 290 nm, t_R= 3.91 min, purity: 97.7%, M/z $[M+H]^+$: 436.

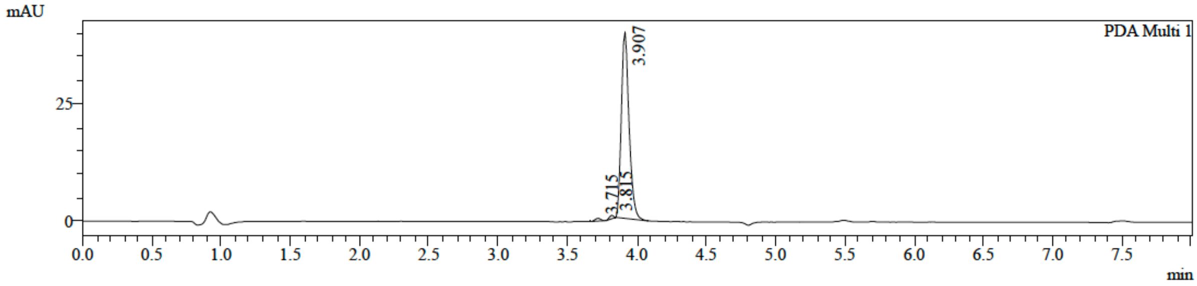

1 PDA Multi 1 / 254nm 4nm

PeakTable

PDA Ch1 254nm 4nm

Peak#	Ret. Time	Area	Height	Name	Area %
1	3.715	1770	621		1.115
2	3.815	1840	786		1.159
3	3.907	155135	39656		97.726
Total		158744	41064		100.000

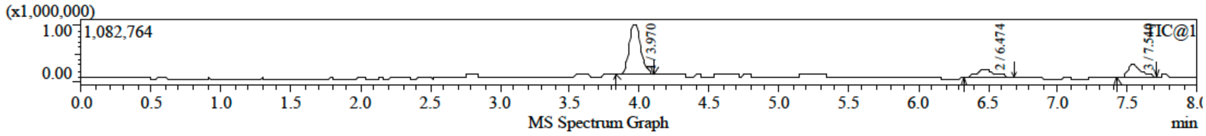

MS Spectrum Graph

#:1 Ret.Time:Averaged 3.960-3.980(Scan#:397-399)
BG Mode:Calc 3.840<->4.110(385<->412)
Mass Peaks:29 Base Peak:436.10(232798) Polarity:Pos Segment1 - Event1

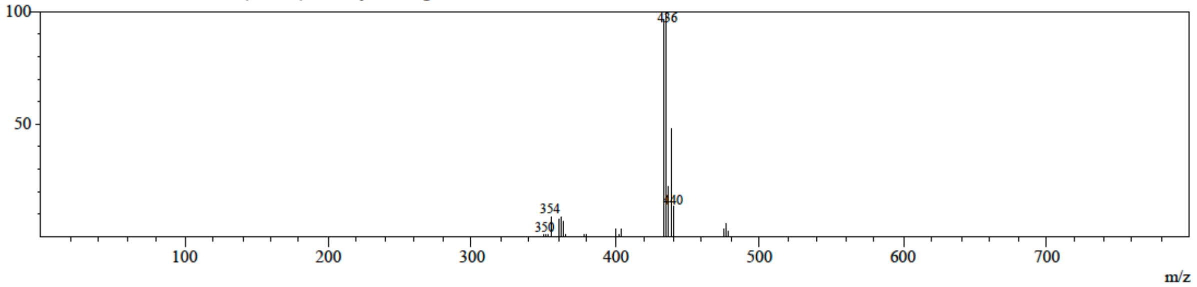

Retention Time : 3.907
Compound Name :
Spectrum Operation : None

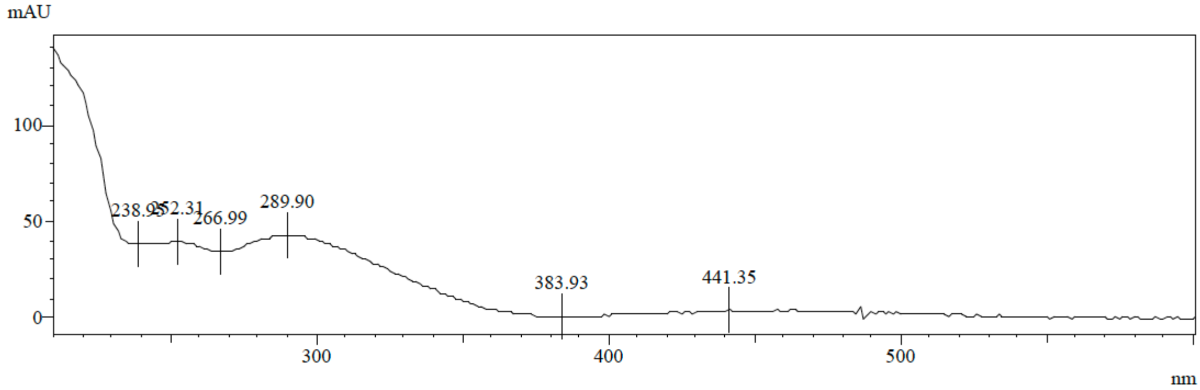

HRMS data of **12d (VUF26175)**

HRMS calcd. for $C_{18}H_{20}Cl_4N_3O$ [M+H]$^+$ = 434.0355, found 434.0357.

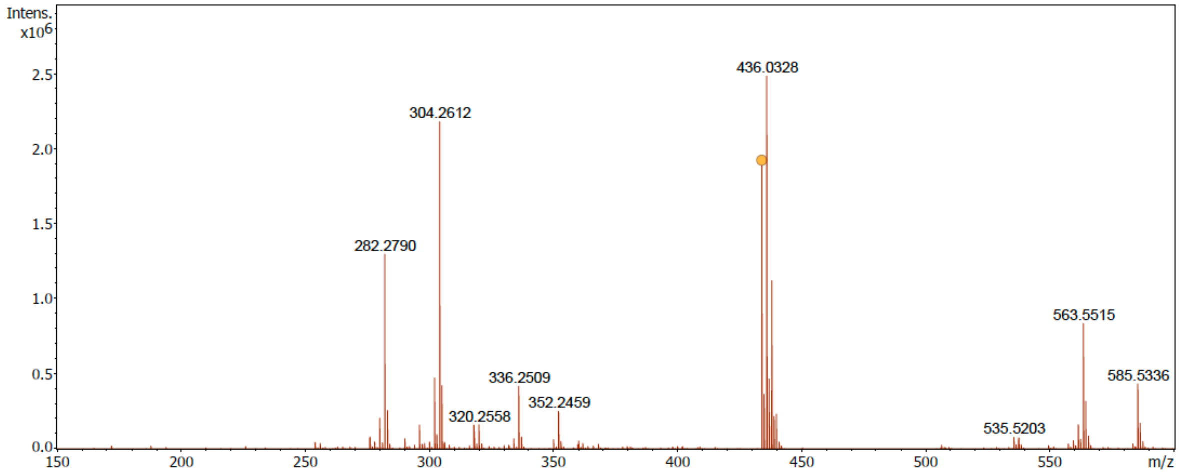

Meas. m/z	#	Ion Formula	m/z	err [ppm]	mSigma	#mSigma	Score	rdb	e⁻ Conf	N-Rule
434.0357	1	C18H20Cl4N3O	434.0355	-0.5	18.8	1	100.00	21.0	even	ok

^{1}H-NMR spectrum of **12e (VUF26202)**

^{1}H NMR (600 MHz, CD_3OD) δ 8.03 – 8.00 (m, 2H), 7.99 (d, *J* = 1.8 Hz, 1H), 7.90 (d, *J* = 1.8 Hz, 1H), 7.66 – 7.60 (m, 3H), 4.92 (dd, *J* = 9.5, 3.4 Hz, 1H), 3.04 (dd, *J* = 12.0, 3.4 Hz, 1H), 2.93 (dd, *J* = 12.0, 9.5 Hz, 1H), 1.27 (s, 9H).

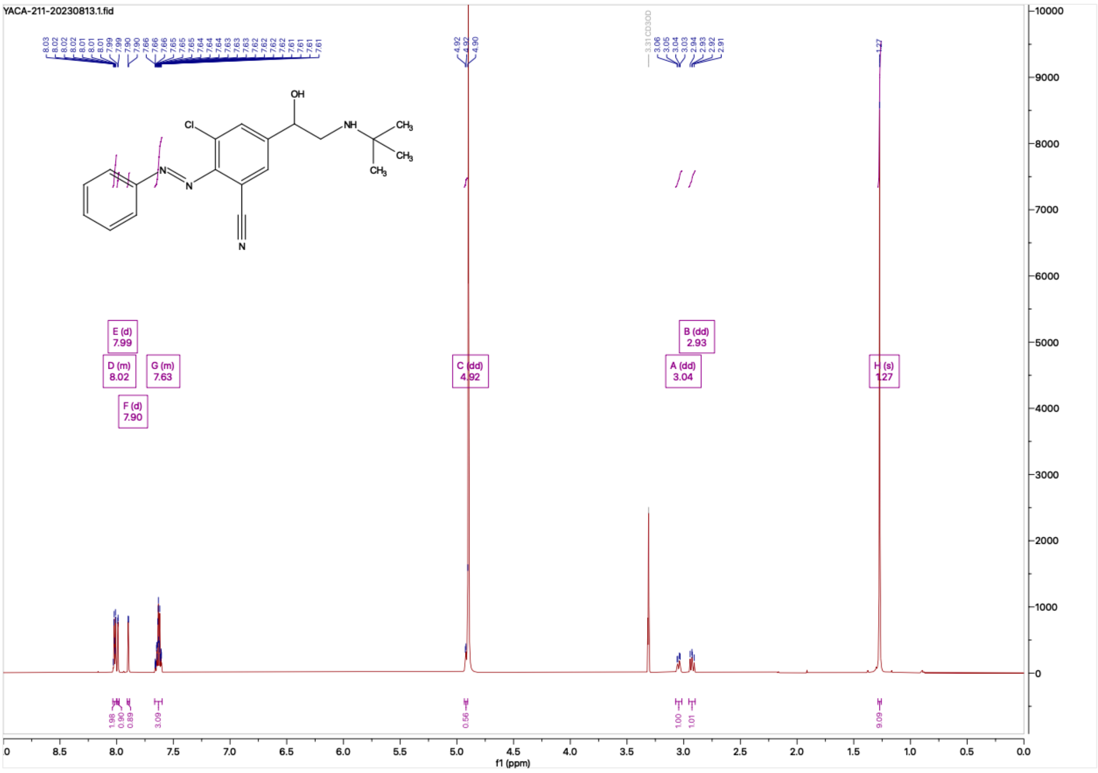

^{13}C-NMR spectrum of **12e (VUF26202)**

^{13}C NMR (151 MHz, CD_3OD) δ 153.5, 151.3, 147.7, 134.4, 134.2, 133.8, 132.3, 130.6, 124.4, 117.5, 104.6, 70.9, 53.5*, 49.9, 27.3.

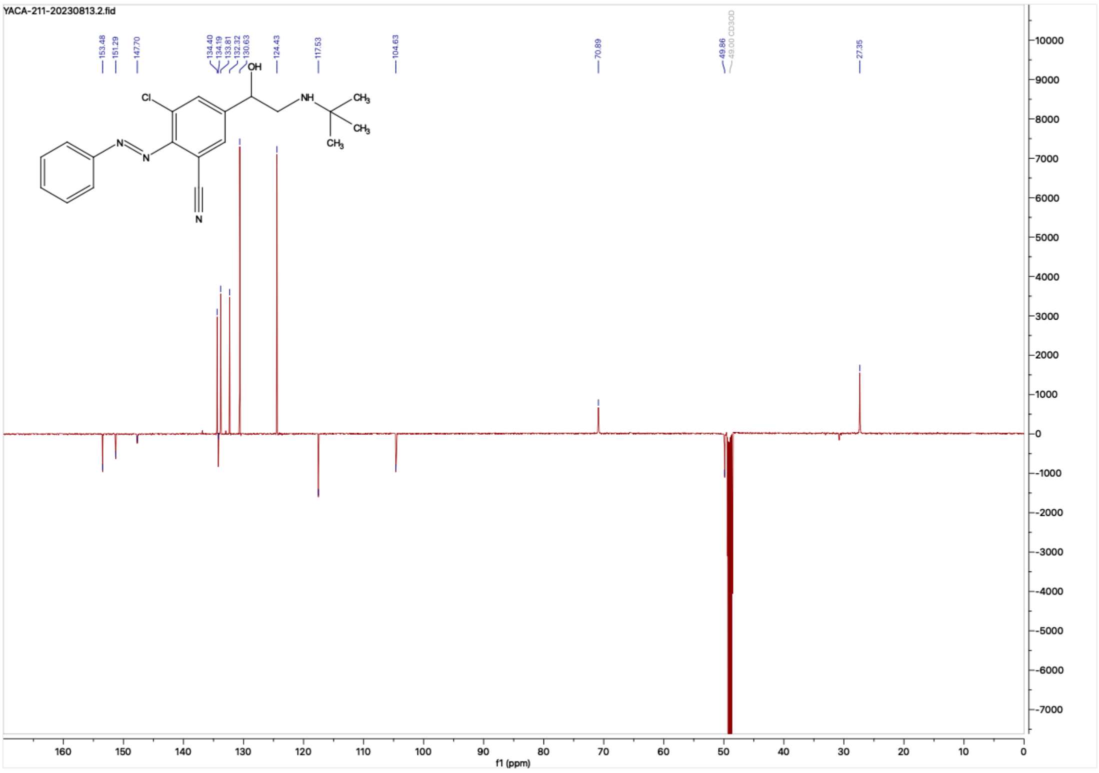

LCMS data of **12e (VUF26202)**

LC-MS: λ_{max}: 325 nm, t_R= 3.55 min, purity: 98.9%, M/z $[M+H]^+$: 357.

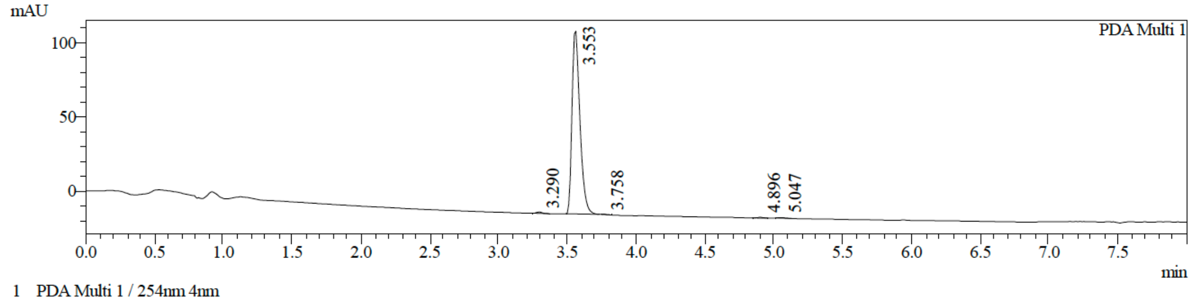

PeakTable

PDA Ch1 254nm 4nm

Peak#	Ret. Time	Area	Height	Name	Area %
1	3.290	2357	884		0.471
2	3.553	495104	124014		98.878
3	3.758	416	191		0.083
4	4.896	1551	439		0.310
5	5.047	1294	409		0.259
Total		500722	125937		100.000

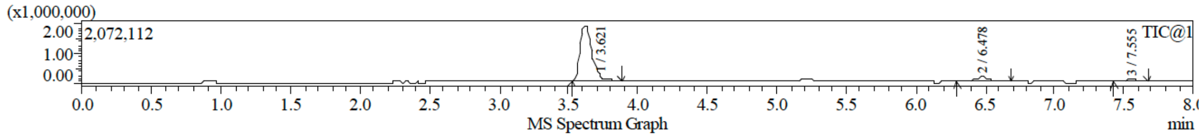

MS Spectrum Graph

#:1 Ret.Time:Averaged 3.610-3.630(Scan#:362-364)
BG Mode:Calc 3.520<->3.880(353<->389)
Mass Peaks:17 Base Peak:357.15(890305) Polarity:Pos Segment1 - Event1

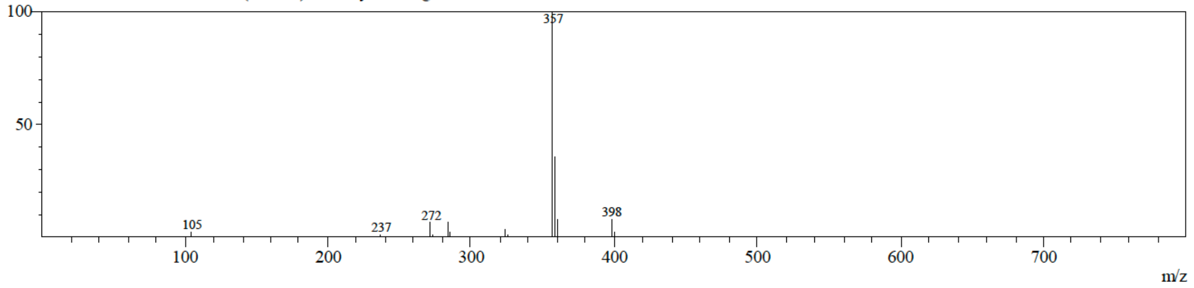

Retention Time : 3.553
Compound Name :
Spectrum Operation : None

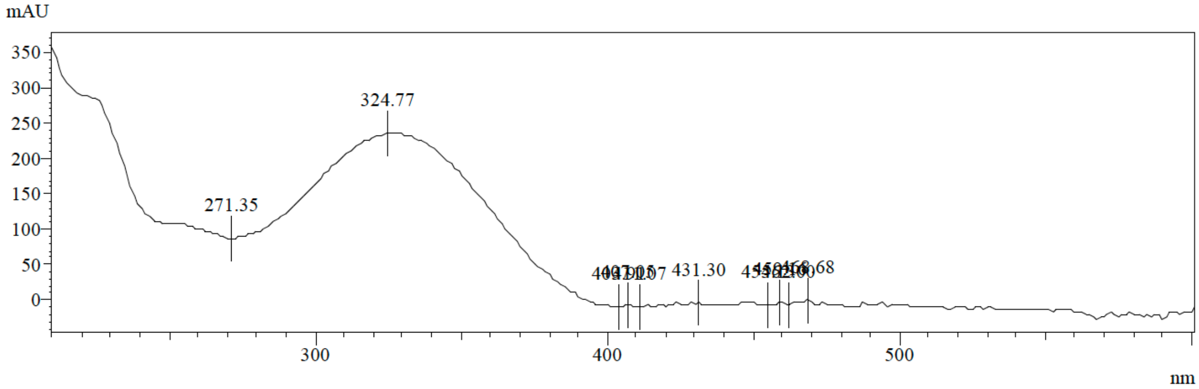

HRMS data of **12e (VUF26202)**

HRMS calcd. for $C_{19}H_{22}ClN_4O$ $[M+H]^+$ = 336.2511, found 336.2503.

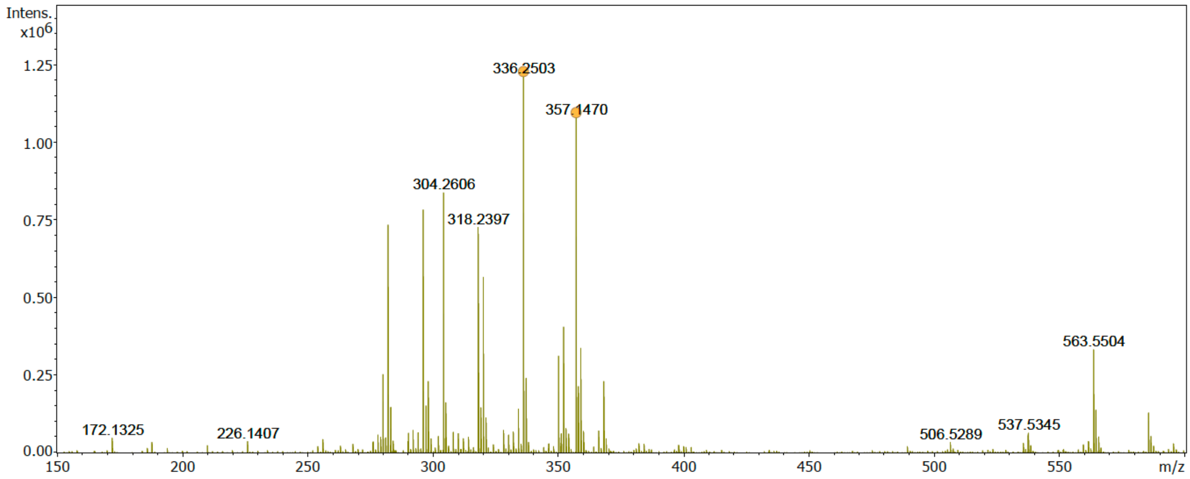

Meas. m/z	#	Ion Formula	m/z	err [ppm]	mSigma	#mSigma	Score	rdb	e⁻ Conf	N-Rule
336.2503	1	C13H33ClN8	336.2511	2.6	169.9	1	100.00	4.5	odd	ok

^{1}H-NMR spectrum of **18 (VUF26211)**

^{1}H NMR (600 MHz, CD_3OD) δ 7.54 (d, J = 2.0 Hz, 1H), 7.39 (d, J = 2.0 Hz, 1H), 4.60 (t, J = 6.7 Hz, 1H), 2.75 (d, J = 6.7 Hz, 2H), 1.18 (s, 9H).

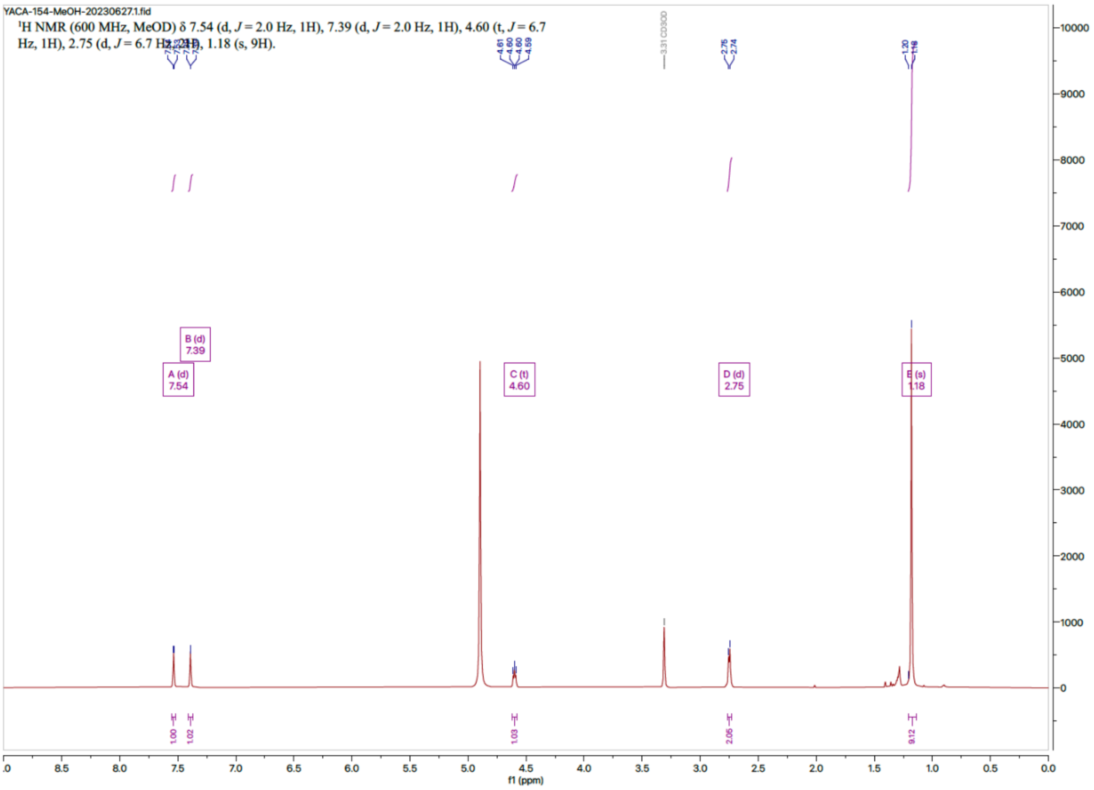

^{13}C-NMR spectrum of **18 (VUF26211)**

^{13}C NMR (151 MHz, CD_3OD) δ 147.97, 133.82, 133.00, 129.97, 120.41, 117.84, 97.20, 71.84, 52.91, 50.53, 28.08.

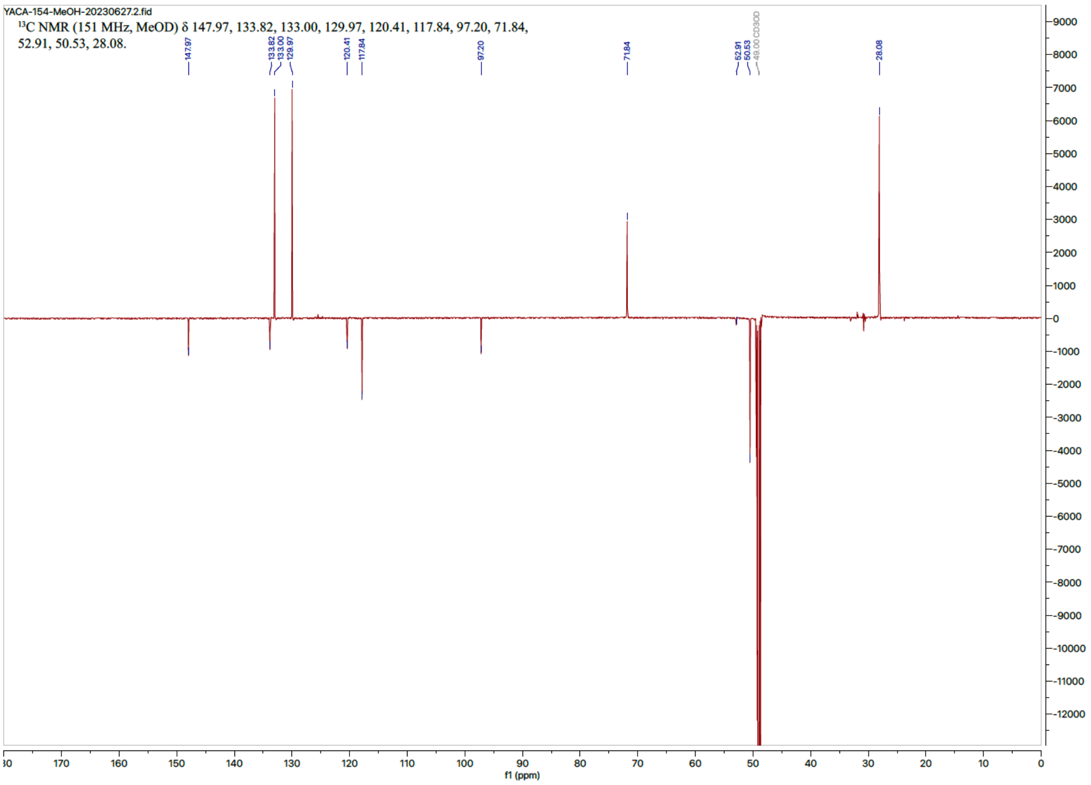

LCMS data of **18 (VUF26211)**

LC-MS: t_R= 2.42 min, purity: 99.2%, M/z [M+H]$^+$: 268.

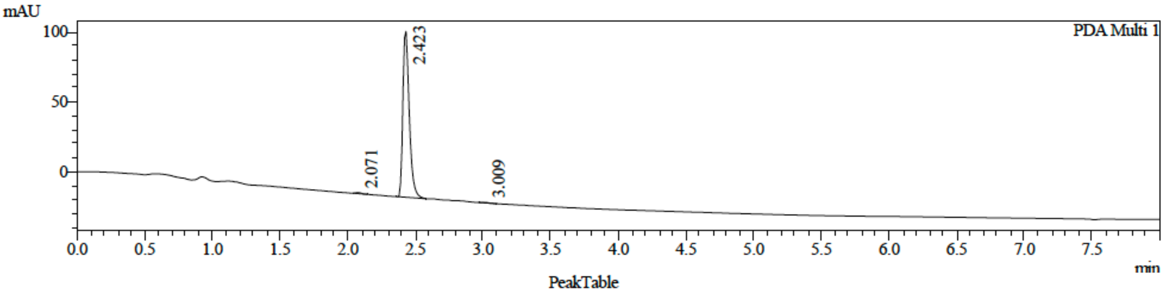

PeakTable

PDA Ch1 254nm 4nm

Peak#	Ret. Time	Area	Height	Name	Area %
1	2.071	2032	725		0.514
2	2.423	392245	118523		99.158
3	3.009	1300	407		0.329
Total		395577	119655		100.000

MS Chromatogram

YACA-154-P C:\LabSolutions\Data\2023\2023-wk25\YACA-154-P.lcd

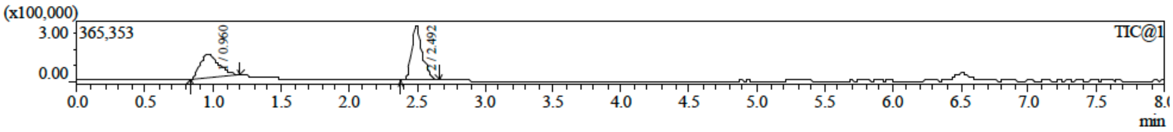

MS Spectrum Graph

#:1 Ret.Time:Averaged 2.480-2.500(Scan#:249-251)

BG Mode:Calc 2.380<->2.660(239<->267)

Mass Peaks:15 Base Peak:268.15(176984) Polarity:Pos Segment1 - Event1

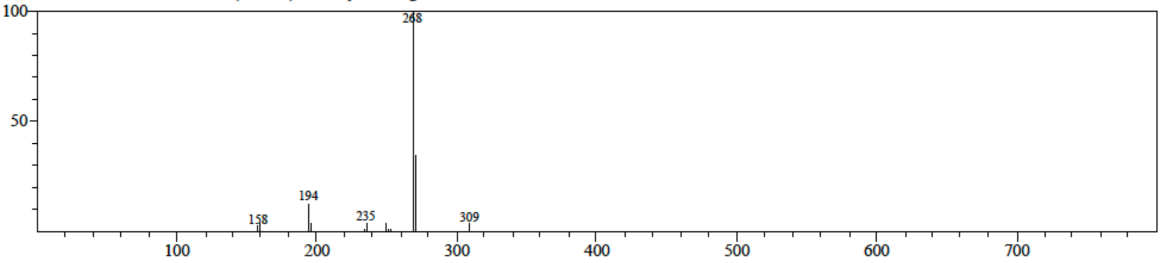

HRMS data of **18** (**VUF26211**)

HRMS calcd. for $C_{13}H_{19}ClN_3O$ $[M+H]^+$ = 268.1211, found 268.1208.

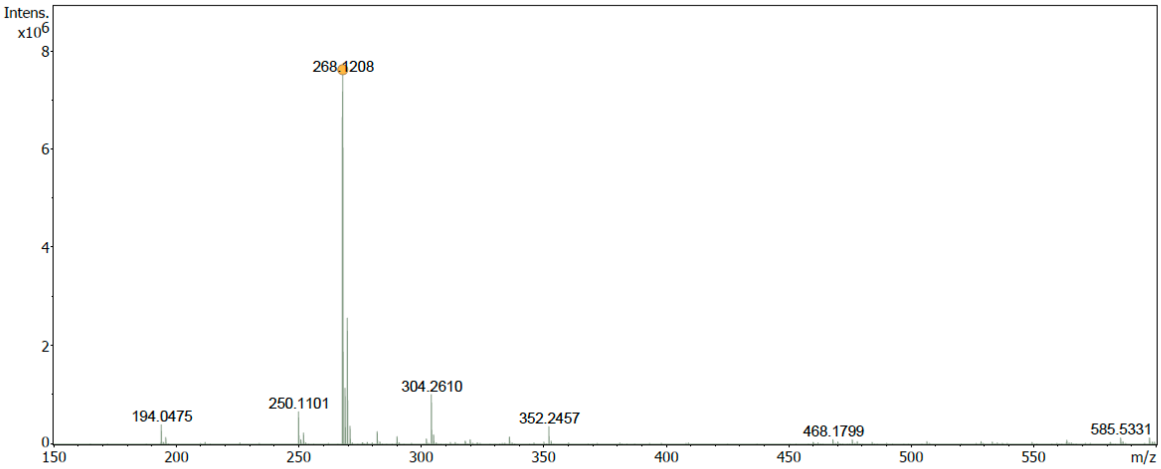

Meas. m/z	#	Ion Formula	m/z	err [ppm]	mSigma	#mSigma	Score	rdb	e⁻ Conf	N-Rule
268.1208	1	C13H19ClN3O	268.1211	1.0	4.8	1	100.00	9.0	even	ok
